# Supplementary material for: Tradeoffs in alignment and assembly-based methods for structural variant detection with long-read sequencing data
Source: Nat Commun. 2024 Mar 19;15:2447. doi: 10.1038/s41467-024-46614-z (PMC10951360; doi:10.1038/s41467-024-46614-z)
Supplement: Supplementary file 1 — Supplementary Information [file 41467_2024_46614_MOESM1_ESM.pdf]

Tradeoffs in alignment and assembly-based methods for structural variant detection with long-read sequencing data

Contents

1 Supplementary Methods **4**
1.1 **Highlights for SV calling pipelines** 4
1.1.1 **Installation and input configuration** 4
1.1.2 **Post-processing** 4
1.2 **Repeat annotation for SV calls** 5
1.3 **Commands used in SV calling pipelines** 5
1.3.1 **Aligners** 5
1.3.2 **Assemblers and auxiliary tools** 6
1.3.3 **Read alignment-based SV callers** 7
1.3.4 **Assembly-based SV callers** 12

2 Supplementary Notes **13**
2.1 **SV calling performance under a set of moderate tolerance parameters** 13
2.2 **Computation cost for different aligners and assemblers** . 13
2.3 **Repeat Annotation for SV calls** 14
2.4 **Evaluation of SV calls across parameters with hap-eval** . 14
2.5 **Orthogonal SV validation with overlapping calls among different tools** 15
2.6 **SV merging by multiple tools to improve SV calling** . . . 16
2.7 **The effects of different aligners for read alignment-based tools on SV calling** 16
2.8 **The effects of different assemblers for assembly-based tools on SV calling** 17
2.9 **Benchmarking most recent deep learning-based SV calling methods** 17

3 Supplementary Tables **19**
1 User recommendation table 20
2 Total number of SV calls and autosomal DELs and INSs 21
3 Read alignment-based SV callers evaluation on Hifi_L1 22

4 Read alignment-based SV callers evaluation on Nano_L1 23
5 Assembly-based SV callers evaluation on Hifi_L1 and Nano_L1 . . 24
6 Computing resource consumption of SV callers 25
7 Computing resource consumption of aligners and assemblers . . . 26
8 Read read alignment-based SV callers evaluation on subsampled Hifi_L1 27
9 Read read alignment-based SV callers evaluation on subsampled Nano_L1 28
10 Assembly-based SV callers evaluation on subsampled Hifi_L1 . . 29
11 Assembly-based SV callers evaluation on subsampled Nano_L1 . 30
12 SVision and INSnet evaluation on Hifi_L1 and Nano_L1 31
13 SVision and INSnet evaluation on subsampled Hifi_L1 and Nano_L1 32

4 Supplementary Figures **33**
1 RepeatMasker annotation for SV calls 33
2 DEL F1 heatmap of read alignment-based tools by tuning P-r on Hifi_L1 34
3 DEL F1 heatmap of read alignment-based tools by tuning O-r on Hifi_L1 35
4 DEL F1 heatmap of read alignment-based tools by tuning p-P on Hifi_L1 36
5 DEL F1 heatmap of read alignment-based tools by tuning p-r on Hifi_L1 37
6 DEL F1 heatmap of read alignment-based tools by tuning P-O on Hifi_L1 38
7 INS F1 heatmap of read alignment-based tools by tuning p-P on Hifi_L1 39
8 INS F1 heatmap of read alignment-based tools by tuning P-r on Hifi_L1 40
9 DEL F1 heatmap, breakpoint shift and sequence similarity by tuning p-O on Nano_L1 41
10 DEL F1 heatmap of read alignment-based tools by tuning P-r on Nano_L1 42
11 DEL F1 heatmap of read alignment-based tools by tuning O-r on Nano_L1 43
12 DEL F1 heatmap of read alignment-based tools by tuning p-P on Nano_L1 44
13 DEL F1 heatmap of read alignment-based tools by tuning p-r on Nano_L1 45
14 DEL F1 heatmap of read alignment-based tools by tuning P-O on Nano_L1 46
15 INS F1 heatmap, breakpoint shift and sequence similarity by tuning p-r on Nano_L1 47
16 INS F1 heatmap of read alignment-based tools by tuning P-p on Nano_L1 48

17 INS F1 heatmap of read alignment-based tools by tuning P-r on Nano_L1 49
18 Definition of *maxdist* and *maxdiff* in hap-eval 50
19 DEL and INS F1 heatmap of hap-eval evaluation by tuning *maxdist* and *maxdiff* on Hifi_L1 51
20 DEL and INS F1 heatmap of hap-eval evaluation by tuning *maxdist* and *maxdiff* on Nano_L1 52
21 Orthogonal SV validation with T2T-CHM13 and Verkko assembly 53
22 Orthogonal SV validation with T2T-CHM13 and Verkko assembly on Nano_L1 55
23 Subsampling effect of different SV callers on Nano_L1. 56
24 Benchmark I on SVision and INSnet 57
25 Benchmark II on SVision and INSnet 58

Supplementary References **59**

1 Supplementary Methods

1.1 Highlights for SV calling pipelines

The details for how to install and run each SV caller are included in our GitHub page. We highlight several essential guidelines in the subsections that follow.

1.1.1 Installation and input configuration

Most SV callers are easy to install since they are available in the Bioconda channel of Anaconda, except for Smartie-sv, PBHoney, and PAV. The original project repository of PBHoney is available on Sourceforge, however, the version of the Blasr aligner [1] used by PBHoney is deprecated and PBHoney crashes on current Blasr releases. We integrated existing patches and added in several modifications to fully migrate PBHoney to Python3, make it compatible with the newer Blasr versions, and simplify the process of PBHoney installation. A script to replicate this process is provided in our GitHub. PAV and Smartie-sv could be installed from their corresponding GitHub repositories. Note that PAV and several other SV callers may run into problems with pysam v0.16, therefore pysam v0.15.2 is recommended instead.

Similar to the aligners mentioned, several SV callers also provided options to specify the sequencing technology, either explicitly defined with an execution parameter, such as Sniffles with its flag "*–ccs-reads*" and NanoVar with its flag "-*x*". Alternatively, some SV callers, such as cuteSV, required a change to several settings for each specific sequencing technology. Typically, read alignment-based SV callers take the reads BAM or SAM file as input, and assembly-based SV callers accept the contigs FASTA file. However, there do exist read alignment-based SV callers which accept reads FASTA/FASTQ files as input since they either have their own alignment pipeline (SKSV) or integrate existing aligners (NanoVar). Additionally, SVIM-asm requires the alignment results of the assembled contigs to perform SV calling. For Smartie-sv, we made two modifications. We replaced Blasr with minimap2 in the alignment step for a fair comparison with other SV callers, and we disabled the output for INDEL as it takes up massive storage and we only focus on SV calls in this paper.

1.1.2 Post-processing

Most SV callers provide a VCF file that contains all SV calls, except for PBHoney and Smartie-sv. PBHoney and Smartie-sv, which use spots and bed files to record the calling results respectively, required additional format conversion before the downstream evaluation and analyses. SVIM and DeBreak additionally distinguish between duplications and insertions and require a manual conversion from duplications to insertions to be fully compatible with the benchmark. Additionally, it requires manual filtering of SV calls based on the number of supporting reads, to remove a massive number of false positive calls. Some SV callers such as NanoSV and PAV output VCF files in various formats, and therefore several modifications are required to be fully compatible with

Truvari [2]. We have provided all post-processing scripts that implement these steps in our GitHub.

1.2 Repeat annotation for SV calls

To reveal the effect of different types of repeat sequences and the ratio of repeat regions on SV calling performance, the SV calling results of four read alignment-based tools (Sniffles, pbsv, cuteSV and SKSV) that provide reference and alternate sequences for both insertions and deletions on Hifi_L1 were compared. For each tool, sequences of reference alleles for deletions and sequences of alternate alleles for insertions were extracted to construct a FASTA file as the input of RepeatMasker (version open-4.0.9) [3]. For each SV, RepeatMasker returns the start and end coordinates of each repeat region and annotates its repeat type. We thus merged all of its repeated regions to calculate the total coverage rate of the repeat region and recorded the types of repeat sequences that appeared in the SV sequence. We further calculated these SV callers' recall, precision, F1 score as well as the SV length distribution on different coverage rates of repeat region and repeat annotation types.

1.3 Commands used in SV calling pipelines

More detailed documentation of tools' usage is available on our GitHub repository (`https://github.com/maiziezhoulab/LRSV_combo`)

1.3.1 Aligners

- **NGMLR**

```
ngmlr -t ${threads} -r ${ref} -q ${reads} -o ${
    outname}.sam --bam-fix -x ${preset}
```

- **Minimap2**

```
minimap2 -t ${threads} --MD -Y -L -a -x ${preset}
    ${ref} ${reads} | samtools sort  -o ${
    output_name}.bam
```

- **Winnowmap**

```
meryl count k=15 output merylDB ${ref}
meryl print greater-than distinct=0.9998 merylDB >
     repetitive_k15.txt
winnowmap --MD -W repetitive_k15.txt -ax ${preset}
     ${ref} ${reads} | samtools sort -o ${outname}.
    bam
```

- **LRA**

```
lra index -CCS ${ref}
lra align -CCS ${ref} ${reads} -t ${threads} --
    printMD -p s | samtools sort -@ 10 -o ${outname
    }.sorted.bam
```

-*CCS* can be replaced by -*CLR*, -*ONT* and -*CONTIG* to fit different input types

1.3.2 Assemblers and auxiliary tools

- **Hifiasm**

```
hifiasm -o ${prefix} -t32 ${reads}
awk '/^S/{print ">"$2"\n"$3}' ${prefix}.bp.hap1.
    p_ctg.gfa | fold > ${prefix}.bp.hap1.p_ctg.fa
awk '/^S/{print ">"$2"\n"$3}' ${prefix}.bp.hap2.
    p_ctg.gfa | fold > ${prefix}.bp.hap2.p_ctg.fa
```

- **HiCanu**

```
canu -p ${prefix} -d ${outdir} genomeSize=3100m
    useGrid=false maxThreads=32 -pacbio-hifi ${
    reads}
```

- **Flye**

```
#Hifi
flye --pacbio-hifi ${reads} --out-dir ${outdir} --
    threads ${threads}
#CLR
flye --pacbio-raw ${reads} --out-dir ${outdir} --
    threads ${threads}
#Nanopore
flye --nano-raw ${reads} --out-dir ${outdir} --
    threads ${threads}
```

- **Peregrine**

```
echo -e "yes\n" | singularity run ${peregrine_sif}
     asm ${reads_fofn} 48 48 48 12 48 12 48 48 48
    --with-consensus --with-alt --shimmer-r 3 --
    best_n_ovlp 8 --output ${out_dir}
```

- **wtdbg2**

```
wtdbg2.pl -t ${threads} -x ${preset} -g 2.9g -o ${
    out_prefix} ${reads}
```

- **IPA**

```
ipa local --nthreads ${threads} --njobs 4 -i ${
   reads}
```

- **Shasta**

```
shasta-Linux-0.10.0 --input ${reads} --config
   Nanopore-UL-Dec2019
```

- **HapDup**

```
minimap2 -ax ${preset} -t 30 ${assembly} ${reads}
   | samtools sort -@ 4 -m 4G >
   assembly_lr_mapping.bam
samtools index -@ 4 assembly_lr_mapping.bam
singularity exec --bind ${outdir} ${hapdup_sif}\
        hapdup --assembly ${assembly} --bam ${
           outdir}/assembly_lr_mapping.bam --out-
           dir ${outdir}/hapdup -t 64 --rtype ${
           hapdup_preset}$
```

- **purge_dups**

  Please kindly refer to our github repository

1.3.3 Read alignment-based SV callers

- **PBHoney**

```
Honey.py spots -n 10 -q 10 -m 70 -i 20 -e 2 -E 2
   --spanMax 10000 --consensus None -o ${prefix}.
   INS --reference ${ref} ${bam}
Honey.py spots -n 10 -q 10 -m 10 -i 20 -e 1 -E 1
   --spanMax 100000 --consensus None -o ${prefix}.
   DEL --reference ${ref} ${bam}
python Convert_to_vcf_PBHoney.py --input ${prefix
   }.DEL.spots --output ${prefix}.DEL.vcf
python Convert_to_vcf_PBHoney.py --input ${prefix
   }.INS.spots --output ${prefix}.INS.vcf
mv ${prefix}.DEL.vcf ${prefix}.DEL_temp.vcf
mv ${prefix}.INS.vcf ${prefix}.INS_temp.vcf
cat ${prefix}.DEL_temp.vcf | awk '{if($1 ~ /^#/ ||
    $5 ~ /<DEL>/){print $0}}' > ${prefix}.DEL.vcf
cat ${prefix}.INS_temp.vcf | awk '{if($5 ~ /<INS
   >/){print $0}}' > ${prefix}.INS.vcf
cat ${prefix}.DEL.vcf ${prefix}.INS.vcf > ${prefix
   }.DEL_INS_merge.vcf
```

```
rm ${prefix}.INS_temp.vcf
rm ${prefix}.DEL_temp.vcf
rm ${prefix}.INS.vcf
rm ${prefix}.DEL.vcf
```

- **NanoSV**

```
NanoSV -t ${threads} ${bam} -o ./${vcf} -s
    samtools -b ${bed}
```

- **Smartie-sv_aln**

```
mkdir variants
samtools view -h ${bam} | ${smartie_sv}/bin/
    printgaps ${ref} variants/${prefix}
python Convert_to_vcf_Smartie-sv.py --input
    variants/${prefix}.svs.bed --output variants/${
    prefix}.svs.vcf --support_thresh 4
```

- **Sniffles**

```
#Hifi
sniffles -t ${threads} --ccs_reads --cluster --
    genotype -m ${bam} -v ${prefix}.vcf
#Others
sniffles -t ${threads} --cluster --genotype -m ${
    bam} -v ${prefix}.vcf
```

- **SVIM**

```
svim alignment --sequence_alleles --min_sv_size 30
     ${workdir} ${bam} ${ref}
cat ${workdir}/variants.vcf | grep -v 'SUPPORT
    =1;\|SUPPORT=2;\|SUPPORT=3;\|SUPPORT=4;\|
    SUPPORT=5;\|SUPPORT=6;\|SUPPORT=7;\|SUPPORT
    =8;\|SUPPORT=9;' > ${prefix}.vcf
```

- **pbsv**

```
pbsv discover -s ${sample} ${bam} ${prefix}.svsig.
    gz
#HIfi
pbsv call --ccs ${ref} ${prefix}.svsig.gz ${prefix
    }.vcf
#CLR
pbsv call ${ref} ${prefix}.svsig.gz ${prefix}.vcf
```

- **NanoVar**

```
nanovar -t ${threads} -x ${preset} ${bam} ${ref} $
    {work_dir}
```

- **cuteSV**

```
#Hifi
cuteSV -t ${threads} \
        --max_cluster_bias_INS 1000 \
        --diff_ratio_merging_INS 0.9 \
        --max_cluster_bias_DEL 1000 \
        --diff_ratio_merging_DEL 0.5 \
        --genotype \
        ${bam} ${ref} ${outvcf} ${work_dir}

#CLR
cuteSV -t ${threads} \
        --max_cluster_bias_INS 100 \
        --diff_ratio_merging_INS 0.3 \
        --max_cluster_bias_DEL 200 \
        --diff_ratio_merging_DEL 0.5 \
        --genotype \
        ${bam} ${ref} ${outvcf} ${work_dir}

#ONT
cuteSV -t ${threads} \
        --max_cluster_bias_INS 100 \
        --diff_ratio_merging_INS 0.3 \
        --max_cluster_bias_DEL 100 \
        --diff_ratio_merging_DEL 0.3 \
```

- **SKSV**

```
# build index
SKSV index ${ref} ${out_dir}
# skeleton-alignment
SKSV aln ${out_dir} ${fastq} --output sk.svseg
# call variants using in.svseg file
SKSV call --genotype --print_allele_seq --read ${
    fastq} sk.svseg ${ref} ${out_prefix}.vcf ${
    out_dir}
```

- **Sniffles2**

```
sniffles --input ${bam} --reference ${ref} --vcf $
    {prefix}.vcf --threads ${threads}
```

- **MAMnet**

```
python ${MAMnetPath}/MAMnet.py -bamfilepath ${bam}
     -threads ${threads} -step 50 -INTERVAL 1e7 -
    genotype True -workdir ${work_dir} -
    SV_weightspath ${MAMnetPath}/type -
    genotype_weightspath ${MAMnetPath}/geno -
    outputpath ./variants.vcf
python MAMnet_convert_to_symbolic.py -i variants.
    vcf -o ${prefix}.vcf
#filter by number of supporting reads (here is 10)
cat #{prefix}.vcf | grep -w -v 'RE=1\|RE=2\|RE=3\|
    RE=4\|RE=5\|RE=6\|RE=7\|RE=8\|RE=9' > ${prefix}
    _REover10.vcf
```

- **DeBreak**

```
debreak -t ${threads} -p ${prefix} --bam ${bam} --
    outpath ${out_dir} --rescue_large_ins --
    rescue_dup --poa --ref ${ref}
mv .debreak.vcf DeBreak.vcf
sed -i 's/#CHROM/##FORMAT=<ID=GT,Number=1,Type=
    String,Description="Genotype">\n#CHROM/g'
    DeBreak.vcf
# DUP as INS
cp DeBreak.vcf DeBreak_DUPasINS.vcf
sed -i "s/<DUP>/<INS>/g" DeBreak_DUPasINS.vcf
sed -i "s/SVTYPE=DUP/SVTYPE=INS/g"
    DeBreak_DUPasINS.vcf
```

- **SVision**

```
#run by chromosome
for i in {1..22}
do
        sample_chr=${sample}_chr${i}
        SVision -t ${threads} -o ${out_dir} -b ${
            bam} -g ${ref} -m ${model_path} -n ${
            sample_chr} -c chr${i}
done

# merge to one vcf
cat ${sample}_chr1.svision.s5.vcf | grep "#" > ${
    sample}.svision.s5.vcf
cat ${sample}_chr*.svision.s5.vcf | grep -v "#" >>
     ${sample}.svision.s5.vcf
rm ${sample}_chr*.svision.s5.vcf
```

```
#rm *.log

## post processing
# modify Filter: Covered, Uncovered, Clustered =>
    PASS; remove CSVs, only keep SVs; tDUP and DUP
    to INS; filter by SUPPORT number (optional),
    threshold=10 (>10) (Default SVision built in
    threshold: >5)
cat ${sample}.svision.s5.vcf | grep -v "##FILTER=<
    ID=Uncovered" | grep -v "##FILTER=<ID=Clustered
    " | sed "s/##FILTER=<ID=Covered,Description=\"
    Covered mean the SV is spanned by reads\">/##
    FILTER=<ID=PASS,Description=\"All filters
    passed\">/g" | sed "s/Covered/PASS/g" | sed "s/
    Uncovered/PASS/g" | sed "s/Clustered/PASS/g" |
    grep -v "<CSV>" | sed "s/INS, DEL, DUP, tDUP (
    tandem duplication) and INV/INS, DEL and INV/g"
     | sed "s/tDUP/INS/g" | sed "s/DUP/INS/g" |
    grep -v 'SUPPORT=1;\|SUPPORT=2;\|SUPPORT=3;\|
    SUPPORT=4;\|SUPPORT=5;\|SUPPORT=6;\|SUPPORT
    =7;\|SUPPORT=8;\|SUPPORT=9;'> ${sample}.svision
    .finals10.vcf
```

- **INSnet**

```
#NOTE: remove one indentation before the
    create_data_long_mul(bamfile_long_path =
    bamfilepath_long, outputpath=outputpath, contig
    =includecontig,max_work = max_work) (move it
    out from the "else") in INSnet.py line 41

mkdir ${work_dir}
#generate features
python ${tool_path}/INSnet.py generate_feature ${
    bam} ${work_dir} ${threads}

#call SV (Hifi)
python ${tool_path}/INSnet.py call_sv ${tool_path
    }/ccs_insertion_weights.h5 ${work_dir} ${bam} $
    {vcf} ${threads}
#call SV (ONT or CLR)
#python ${tool_path}/INSnet.py call_sv ${tool_path
    }/insertion_weights.h5 ${work_dir} ${bam} ${vcf
    } ${threads}

#clean up to save storage
```

```
rm -r ${work_dir}

#convert to symbolic vcf
python MAMnet_convert_to_symbolic.py -i ${vcf} -o
    ${vcf%%.vcf}_symbolic.vcf
```

1.3.4 Assembly-based SV callers

- **Dipcall**

```
run-dipcall -t ${threads} -x ${bed} ${prefix} ${
    ref} ${hp1} ${hp2} > ${prefix}.mak
make -j2 -f ${prefix}.mak
```

- **Smartie-sv_asm**

  Please kindly refer to our github repository

- **SVIM-asm**

```
minimap2 -a -x asm5 --cs -r2k -t 12 ${ref} ${hp1}
    > alignments_contig.1.sam
minimap2 -a -x asm5 --cs -r2k -t 12 ${ref} ${hp2}
    > alignments_contig.2.sam
samtools sort -m4G -@4 -o alignments_contig.1.
    sorted.bam alignments_contig.1.sam
samtools sort -m4G -@4 -o alignments_contig.2.
    sorted.bam alignments_contig.2.sam
samtools index alignments_contig.1.sorted.bam
samtools index alignments_contig.2.sorted.bam
svim-asm diploid ${prefix} alignments_contig.1.
    sorted.bam alignments_contig.2.sorted.bam ${ref
    }
rm *.sam*
rm *.bam*
```

- **PAV**

  Please kindly refer to our github repository

2 Supplementary Notes

2.1 SV calling performance under a set of moderate tolerance parameters

We first examined the performance of read alignment-based SV calling methods. Fig. 2a-d shows the general trend of the length distribution of SVs from 12 read alignment-based methods on Hifi_L1. The number of SVs decreased sharply as a function of size, with most SVs clustering in the 50-400bp range. We found that these calling tools had similar length distributions for deletions, while they varied considerably for insertions. NanoSV and Smartie-sv_aln called the most short insertions but far fewer large insertions (Fig. 2c-d). On the contrary, pbsv called more large insertions, but relatively fewer small insertions. Only 5 out of 12 read alignment-based SV callers called any insertions over 10kb. PBHoney and Smartie-sv_aln failed to call any SVs larger than 5kb.

Similarly, we evaluated the performance of assembly-based SV calling methods. The SV length distribution from four callers on Hifi_L1 is shown in Fig. 2e-h. The result indicates that length distribution patterns among assembly-based tools are generally similar except for Smartie-sv_asm, which called more SVs than the other tools across most size ranges. Assembly-based tools showed a similar trend in deletion length distribution as read alignment-based tools, while detecting more large insertions than most alignment-based tools, especially for insertions over 1kb.

We next examined performance in the ONT dataset. The length distribution of SVs on Nano_L1 is shown in Fig. 2i-p. Some tools were not applicable to ONT datasets, we thus used 9 alignment-based and 3 assembly-based SV calling methods in this analysis. As was the case for the PacBio results, the number of SV calls dropped sharply as a function of SV size. Among read alignment-based tools, Smartie-sv_aln called more short deletions (50-200bp) than other tools (Fig. 2i), and NanoSV called three times as many short insertions than other tools (Fig. 2k). NanoVar called fewer short SVs than other tools (Fig. 2i and k), but detected more SVs larger than 10kb (Fig. 2j and l). Among the three assembly-based tools tested (Dipcall, Smartie-sv_asm, and PAV), PAV called the largest number of SVs, and Dipcall detected the least SVs in all size intervals, except for insertions over 7.5kb. Assembly-based SV callers generated fewer deletions in the range of 50-200bp than read alignment-based SV callers.

2.2 Computation cost for different aligners and assemblers

We also evaluated compute time of different aligners. These were generally clustered together, and their order of performance depended on the library. For Hifi_L1, Winnowmap [4] used the fewest CPU hours (174), followed by minimap2 [5] (189), NGMLR [6] (293), and LRA [7] (294) (black bars in Fig. 2u and Supplementary Table 7). The aligner NGMLR used the fewest CPU hours (97), followed by LRA (324), Winnowmap (400), and minimap2 (593) for Nano_L1 (gray bars in Fig. 2u and Supplementary Table 7).

A much greater variance in CPU time was observed for different assemblers. For Hifi_L1 Hifiasm used the fewest CPU hours (440), followed by wtdbg2 (757), Flye (1895), HiCanu (2949), IPA (4717), and Peregrine (5386) (black bars in Fig. 2v and Supplementary Table 7). For Nano_L1 Shasta used the least CPU hours (598), followed by Flye (701) and wtdbg2 (1446) (gray bars in Fig. 2v and Supplementary Table 7).

All four aligners consumed less than 50Gb memory; all assemblers consumed 150-400Gb memory, except for IPA using 88Gb when assembling Hifi_L1 (Supplementary Table 7).

2.3 Repeat Annotation for SV calls

We selected four read alignment-based SV callers, Sniffles, pbsv, cuteSV and SKSV, that provided alternate sequences for both insertions and deletions and evaluated their performance on SVs of different repeat types. We used RepeatMasker to annotate all SV calls, and the detailed method was described in the Methods section. In Supplementary Fig. 1, we demonstrated these four tools' performance on four main repeat annotation types (SINE/Alu, Simple repeat, LINE/L1 and Retroposon/SVA) and those SVs not involving any repeats (marked as "None"). All SV callers showed similar performance across these five classes of SVs. The F1 scores on SINE/Alu and LINE/L1 were relatively higher than the rest of annotation types. Compared to the other three tools, pbsv seemed to be limited in recalling SVs not involving repeats and Retroposon/SVA. These results indicated the SV accuracy of long-read SV callers was not affected by different types of repeats.

2.4 Evaluation of SV calls across parameters with hap-eval

In addition to the SV benchmarking tool Truvari, we also used hap-eval [8] to investigate the robustness of all tools across evaluation parameters using grid search on both Hifi_L1 and Nano_L1. Hap-eval generates pseudo-haplotypes via inserting a set of nearby SVs into the reference genome, to benchmark SVs. Instead of evaluating each SV independently with Truvari, hap-eval evaluates multiple SVs together, using two key parameters: *maxdist* and *maxdiff*. The benchmarking procedure is as follows: Hap-eval first identifies SVs from both the benchmark and VCF being evaluated that are within a certain distance along the reference genome (determined by *maxdist*) and evaluates them as a whole set. Secondly, for each VCF, hap-eval generates all possible pseudo-haplotypes based on the genotypes of SVs by inserting all SVs within the same set into the reference sequence. In the final evaluation step, if the sequence difference between the pseudo-haplotypes from the VCF being evaluated and the benchmark VCF is below a certain threshold (determined by *maxdiff*), all SVs from the same set are considered true positives. A detailed pipeline of hap-eval evaluation and the definition of *maxdist* and *maxdiff* are illustrated in Supplementary Fig. 18. We observed similar patterns and robustness for most

of the tools in grid search experiments using hap-eval as in previous grid search experiments using Truvari. The performance of SV callers gradually dropped as two parameters were set to be more stringent (small *maxdist* and *maxdiff*). Three assembly-based tools (Dipcall, SVIM-asm, and PAV) and one alignment-based tool (pbsv) exhibited significantly higher F1 scores than other SV callers under the most stringent criteria (*maxdist=0* and *maxdiff=0*) (Supplementary Fig. 19 and Supplementary Fig. 20). In general, insertion SVs were more sensitive to changes of parameters. PBHoney, Smartie-sv_aln, and NanoSV were excluded from this analysis since their outputs were not supported by hap-eval. PBHoney and Smartie-sv_aln did not output genotyping information and the VCF file by NanoSV did not contain *PASS* in the *FILTER* field.

2.5 Orthogonal SV validation with overlapping calls among different tools

Although benchmarking against the GIAB SV gold standard is an efficient and precise procedure to evaluate and compare the SV calling performance of different tools, the GIAB gold standard SV callset is not a complete set and could also contain false positives. Relying on the conjecture that SVs supported by more tools are more likely to be true positives than SVs supported by fewer tools, we analyzed overlapping SV calls among 11 read alignment-based or 4 assembly-based SV calling methods, and separated them into three categories by comparing with the benchmark callset: true positives (TPs), false positives (FPs), and false negatives (FNs). NanoSV is not applicable to this analysis. The detailed method is described in the Methods section. To perform this analysis, a set of fixed and moderate tolerance parameters (*p=0, P=0.5, r=500, O=0*) in Truvari was used. As mentioned before, parameter p was set to zero to disable the SV sequence comparison since five alignment-based tools do not provide alternate allele sequences for insertions, and the parameter O was set to zero to allow breakpoint shift for deletions, which was beneficial for most alignment-based tools. To use benchmark SVs, high-confidence SVs (N = 9397) determined in high-confidence regions by GIAB are often utilized by the community. Without these constraints, we can also consider all SVs from the benchmark (N = 28745). This SV overlapping analysis with constraints on Hifi_L1 showed 88.1% TP deletions from the benchmark were detected by all 11 read alignment-based methods, whereas, only 30.9% TP insertions from the benchmark were detected by 11 read alignment-based methods (Fig. 4a, top panels). We then analyzed overlapping SV calls among 4 assembly-based methods (Fig. 4b, top panels). A total of 94.8% TP deletions and 92.2% TP insertions relative to the benchmark were detected by all 4 assembly-based tools. These results indicate that most of TP SV calls are supported by most of the tools. Assembly-based tools are better in detecting TP insertions than read alignment-based tools since half of read alignment-based SV callers, NanoVar, PBHoney, SVIM, pbsv, and Sniffle only discover 58.4% - 79.7% of TP insertions relative to the benchmark.

2.6 SV merging by multiple tools to improve SV calling

Although most of TP SVs were supported by most SV callers, many tools generated a substantial number of exclusive TP SV calls, especially for insertions. The implication of this finding is that the number of TP SV calls could be further increased if we merged SV calls from multiple tools. To investigate the strength of merging results in Hifi_L1, we chose five read alignment-based SV callers that either had good SV calling performance or generated unique TP SV calls, namely Sniffles, SVIM, cuteSV, pbsv, and SKSV. We also selected the three best-performing assembly-based SV callers, Dipcall, SVIM-asm, and PAV. We merged SV calls from all 8 tools and achieved remarkable recall rates for both deletions and insertions of 99.1% and 97.2%, respectively. Recall increased by 0.7% and 1.2% for deletions and insertions, compared to the best tool for each SV type. Our findings provide guidance for designing new SV callers and how to maximize and utilize discordance signals from both read and contig alignments. Although simply merging SV calls from multiple tools could significantly increase the recall, false positives also notably rose, resulting in a decreased F1. For downstream analysis, a SV filtering pipeline is necessary to remove false positives.

2.7 The effects of different aligners for read alignment-based tools on SV calling

Considering that different aligners could affect the performance of read alignment-based SV callers, we selected three read alignment-based tools, Sniffles, SVIM, and cuteSV, which had relatively higher performance on both Hifi_L1 and Nano_L1, and evaluated the effect offour different aligners (NGMLR, minimap2, Winnowmap and LRA) on their SV calling performance. We used radar plots to demonstrate the SV calling performance of three tools across four metrics on Hifi_L1 (Fig. 6a).

All three SV callers showed consistently higher recall when using the minimap2, Winnowmap or LRA aligners compared to the NGMLR aligner, for insertions (Fig. 6a, top panel), though the difference was small in cuteSV. For Nano_L1 dataset (Fig. 6a, bottom panel), we observed a similar trend for the effect of four aligners on insertion recall, except in cuteSV. We also observed that Sniffles and SVIM achieved higher insertion precision when running SV calling from the alignment results of NGMLR and LRA on Nano_L1. Even though we observed a similar pattern for insertion precision on Hifi_L1, this difference was fairly subtle. For both Hifi_L1 and Nano_L1 datasets, performance on deletions for all three SV callers was relatively unaffected by different aligners. Winnowmap and minimap2 showed similar effects on different read alignment-based SV callers across long-read datasets. This result could be partially explained by the fact that Winnowmap is developed based on minimap2. Compared to Sniffles and SVIM, cuteSV was influenced the least by different aligners. In general, although the effect may be mitigated by optimizing SV calling algorithms, using different aligners does affect SV calling performance,

especially for insertions, and the extent of the difference may vary across different datasets.

2.8 The effects of different assemblers for assembly-based tools on SV calling

We similarly evaluated the effects of six different assemblers on Hifi_L1 and three different assemblers on Nano_L1, using three assembly-based SV callers. The radar plot of Fig. 6b, top panel, shows that Dipcall was affected the most by the choice of assembler, followed by SVIM-asm and PAV. For deletion and insertion recall, assembly from Hifiasm and Peregrine + HapDup achieved the best result on Dipcall, followed by wtdbg2 + HapDup, Flye + HapDup, IPA and HiCanu + purge_dups. For deletion and insertion precision, only assembly relying on IPA generated a low rate on Dipcall. In SVIM-asm, only the insertion precision was significantly affected by the six assemblers, and Hifiasm and Peregrine + HapDup achieved the best result. In PAV, both deletion and insertion recall were significantly affected by the six assemblers, and Hifiasm, IPA and Peregrine + HapDup achieved the best result. In all three SV callers, deletion and insertion precision were poor when using assembly from IPA. In general, assembly results from Hifiasm achieved the best SV calling on all three callers, followed by Peregrine + HapDup and wtdbg2 + HapDup. The radar plots for Nano_L1 in Fig. 6b (bottom panel) showed that Flye+HapDup, Shasta+HapDup and wtdbg2+HapDup had similar effects on Dipcall, SVIM-asm and PAV, although all three assembly-based SV callers showed a slightly lower recall in both deletion and insertion when using assembly from wtdbg2+HapDup.

2.9 Benchmarking most recent deep learning-based SV calling methods

Recently, several new deep learning-based SV calling methods for long reads have been proposed. SVision identifies the matched and unmatched bases between each variant-supporting read and its aligned segment on the reference genome to encode the input images. It then applies a convolutional neural network to detect SV breakpoints from these images. INSnet, similar to MAMnet, divides the reference genome into multiple sub-regions and constructs alignment matrices. It then leverages the convolutional block attention module and efficient channel attention module to compute a variant feature vector from each alignment matrix. It finally uses a bidirectional gated recurrent unit network to detect insertions, specifically. We benchmarked SVision and INSnet using our established evaluation framework, which allowed us to do an efficient comparison with previously evaluated methods.

SV length distribution plots demonstrated that SVision had similar length distributions for deletion and insertion SVs as other alignment-based methods but called more large deletion SVs over 10kb on Hifi_L1 (Supplementary Fig. 24a). INSnet, designed specifically for insertion SVs, did call more large insertions over 1kb than most other alignment-based methods on Hifi_L1 (Supple-

mentary Fig. 24b). The F1 performance of SVision and INSnet was robust to changes of SV size, and they were similar to three high-performing alignment-based tools (SKSV, cuteSV, and MAMnet) (Supplementary Fig. 24e-f). The run-time cost of SVision and INSnet was between the tier 1 and tier 2 ranges we defined in the user recommendation Supplementary Table 1 (17 and 43 CPU hours by INSnet for Hifi_L1 and Nano_L1, respectively, and 37 and 82 CPU hours by SVision for Hifi_L1 and Nano_L1, respectively) (Supplementary Fig. 24g). However, INSnet consumed more than 100GB of memory for the ONT datasets. By further investigating the robustness of these tools, we observed that the deletion performance of SVision was also sensitive to changes of parameters, similar to most other alignment-based tools (Supplementary Fig. 24h-i). Neither tool provides alternate allele sequences by default, and for this reason, the sequence comparison was disabled for insertions. Similar to other alignment-based tools, the performance of SVision and INSnet was not robust to changes of evaluation parameters due to a wide range distribution of breakpoint shift (Supplementary Fig. 24h-i).

The benchmarking on the subsampling effect showed that SVision and INSnet demonstrated relatively stable performance across different sequencing coverages until the coverage decreased to 10x or lower (Supplementary Fig. 25a-d). However, their F1 performance was not better than robust tools like Sniffle2. Across 11 PacBio and ONT datasets, we found that SVision achieved fairly stable and high F1 scores for deletion SVs regardless of different coverage or insert sizes in different libraries, but not good as the four alignment-based tools (cuteSV, Sniffles2, MAMnet, and DeBreak). INSnet achieved fairly stable and high F1 scores for insertion SVs, and its performance was comparable to or slightly worse than MAMnet across all datasets (Supplementary Fig. 25e). Finally, for complex SVs, SVision can detect duplications (DUP) and inversions (INV), but INSnet is only designed to detect insertions. The DUP and INV performance of SVision was not better than high-performing tools like pbsv, NanoVar, and DeBreak (Supplementary Fig. 25f).

More deep learning-based approaches (built on read alignment) are expected to be introduced and generate high and robust performance. These methods are promising in detecting SVs compared to traditional alignment-based methods. However, they still suffer from the same problems as traditional alignment-based methods, for example, their performance is sensitive to changes of evaluation parameters. INSnet, designed specifically for insertions, did achieve the second-best result for insertions ranging from 50bp to 1kb under a set of moderate tolerance evaluation parameters (Supplementary Table 1).

3 Supplementary Tables

			tier1	tier2	tier3
**DEL and INS (Evaluation across threshods of different parameters)(Pacbio)**	SV detection accuracy robust to evaluation parameters	DEL	PAV, SVIM-asm, Dipcall, pbsv	smartie-sv_asm, DeBreak	rest of the tools
		INS	PAV, SVIM-asm, Dipcall, pbsv	SKSV, cuteSV	rest of the tools
	Accuracy for breakpoint	DEL	PAV, SVIM-asm, Dipcall, pbsv, DeBreak, Smartie-sv_asm	rest of the tools	PBHoney
		INS	PAV, SVIM-asm, Dipcall, pbsv, DeBreak, Smartie-sv_asm	rest of the tools	PBHoney
	Accuracy for sequence similarity	DEL	PAV, SVIM-asm, Dipcall, pbsv	cuteSV, Sniffles,SVIM, SKSV, Sniffles2	rest of the tools
		INS	PAV, SVIM-asm, Dipcall, pbsv	cuteSV, Sniffles,SVIM, SKSV, Sniffles2	rest of the tools
	Speed (tier1: 0-30h; tier2: 30-100h; tier3: >100h)		cuteSV,MAMnet,SVIM,PBHoney, Dipcall,INSnet,Smartie-sv_aln, Sniffles,Sniffles2	SVIM-asm,SVision,pbsv,SKSV,NanoVar	PAV,Smartie-sv_asm,DeBreak,NanoSV
			**tier1**	**tier2**	**tier3**
**DEL and INS (Evaluation across threshods of different parameters)(ONT)**	SV detection accuracy robust to evaluation parameters	DEL	PAV, Dipcall, SVIM-asm	DeBreak	rest of the tools
		INS	PAV,SVIM-asm	Dipcall	rest of the tools
	Accuracy for breakpoint	DEL	PAV, Dipcall, SVIM-asm, Sniffles, DeBreak, Sniffles2	MAMnet, Smartie-sv_aln	rest of the tools
		INS	PAV, Dipcall, SVIM-asm, Sniffles, DeBreak, Sniffles2	MAMnet, INSnet, Smartie-sv_aln	rest of the tools
	Accuracy for sequence similarity	DEL	PAV, Dipcall, SVIM-asm	cuteSV, Sniffles, SVIM, Sniffles2	rest of the tools
		INS	PAV, Dipcall, SVIM-asm	cuteSV, Sniffles, SVIM, Sniffles2	rest of the tools
	Speed (tier1: 0-30h; tier2: 30-100h; tier3: >100h)		cuteSV,Sniffles,MAMnet,SVIM, SVIM-asm,Dipcall	INSnet,Sniffles2,SVision,NanoVar	NanoSV,Smartie-sv_aln,DeBreak,PAV

Supplementary Table 1: User recommendation table. For each evaluation scenario, it lists methods by tier 1-3.

SV callers	Hifi_L1		Nano_L1	
	All SV calls	Autosomal DELs and INSs	All SV calls	Autosome DELs and INSs
PBHoney	16,916	16,916	N/A	N/A
NanoSV	42,914	25,085	60,042	42,848
Smartie-sv_aln	20,561	20,561	30,538	30,538
Sniffles	24,947	16,848	26,239	21,724
SVIM	23,990	18,273	25,321	22,692
cuteSV	25,910	18,023	30,715	23,126
NanoVar	34,380	17,798	31,265	15,278
pbsv	30,257	17,851	N/A	N/A
SKSV	24,926	19,883	N/A	N/A
Sniffles2	26,810	20,747	27,854	23,222
MAMnet	18,796	16,843	22,629	19,108
DeBreak	23,544	20,731	23,254	21,331
Dipcall	25,695	24,774	23,741	22,980
Smartie-sv_asm	29,646	29,646	N/A	N/A
SVIM-asm	28,772	23,669	24,517	21,928
PAV	26,242	23,218	26,257	23,310

Supplementary Table 2: Total number of SV calls (>= 50bp) and autosomal DELs and INSs from each SV caller on Hifi_L1 and Nano_L1.

DEL		PBHoney (2014)	NanoSV (2017)	Smartie-sv_aln (2018)	Sniffles (2018)	SVIM (2019)	cuteSV (2020)	NanoVar (2020)	pbsv (2021)	SKSV (2021)	Sniffles2 (2022)	MAMnet (2022)	DeBreak (2022)
Total Benchmark Calls (>50): 4116													
50˜1k	TP	3,528 (0)	2,945 (2,758)	3,531 (0)	3,550 (1,622)	3,560 (3,510)	3,552 (3,506)	3,505 (3,361)	3,493 (3,458)	3,540 (3,451)	3,567 (3,525)	3,527 (2,331)	3,547 (3,407)
	FP	335	790	279	91	110	84	266	136	98	108	68	96
	FN	77	2,872	74	55	45	53	100	112	65	38	78	58
	Recall	97.9% (0%)	50.6% (49.0%)	97.9% (0%)	98.5% (96.7%)	98.8% (98.7%)	98.5% (98.5%)	97.2% (97.1%)	96.9% (96.9%)	98.2% (98.2%)	98.9% (98.9%)	97.8% (96.8%)	98.4% (98.3%)
	Precision	91.3% (0%)	78.8% (73.8%)	92.7% (0%)	97.5% (44.5%)	97.0% (95.6%)	97.7% (96.4%)	92.9% (89.1%)	96.3% (95.3%)	97.3% (94.9%)	97.1% (95.9%)	98.1% (64.8%)	97.4% (93.5%)
	F1	94.5% (NA)	61.7% (58.9%)	95.2% (NA)	98.0% (61.0%)	97.9% (97.2%)	98.1% (97.5%)	95.0% (92.9%)	96.6% (96.1%)	97.7% (96.5%)	98.0% (97.4%)	98.0% (77.6%)	97.9% (95.9%)
1k˜10k	TP	351 (0)	383 (352)	347 (0)	477 (263)	473 (463)	480 (474)	463 (428)	470 (417)	477 (467)	439 (363)	452 (292)	478 (462)
	FP	7	187	9	12	22	5	39	9	5	25	9	4
	FN	130	350	134	4	8	1	18	11	4	42	29	3
	Recall	73.0% (0%)	52.3% (50.1%)	72.1% (0%)	99.2% (98.5%)	98.3% (98.3%)	99.8% (99.8%)	96.3% (96.0%)	97.7% (97.4%)	99.2% (99.2%)	91.3% (89.6%)	94.0% (91.0%)	99.4% (99.4%)
	Precision	98.0% (0%)	67.2% (61.8%)	97.5% (0%)	97.5% (53.8%)	95.6% (93.5%)	99.0% (97.7%)	92.2% (85.3%)	98.1% (87.1%)	99.0% (96.9%)	94.6% (78.2%)	98.0% (63.3%)	99.2% (95.9%)
	F1	83.7% (N/A)	58.8% (55.3%)	82.9% (N/A)	98.4% (69.6%)	96.9% (95.9%)	99.4% (98.8%)	94.2% (90.3%)	97.9% (92.0%)	99.1% (98.0%)	92.9% (83.5%)	96.0% (74.7%)	99.3% (97.6%)
>10k	TP	0 (0)	16 (15)	0 (0)	29 (14)	29 (27)	29 (27)	27 (22)	28 (22)	29 (27)	25 (10)	24 (3)	28 (27)
	FP	0	1	0	4	1	1	2	3	0	3	1	2
	FN	29	26	29	0	0	0	2	1	0	4	5	1
	Recall	0% (0%)	38.1% (36.6%)	0% (0%)	100.0% (100.0%)	100.0% (100.0%)	100.0% (100.0%)	93.1% (91.7%)	96.6% (95.7%)	100.0% (100.0%)	86.2% (71.4%)	82.8% (37.5%)	96.6% (96.4%)
	Precision	0% (0%)	94.1% (88.2%)	0% (0%)	87.9% (42.4%)	96.7% (90.0%)	96.7% (90.0%)	93.1% (75.9%)	90.3% (71.0%)	100.0% (93.1%)	89.3% (35.7%)	96.0% (12.0%)	93.3% (90.0%)
	F1	N/A (N/A)	54.2% (51.7%)	N/A (N/A)	93.5% (59.6%)	98.3% (94.7%)	98.3% (94.7%)	93.1% (83.0%)	93.3% (81.5%)	100.0% (96.4%)	87.7% (47.6%)	88.9% (18.2%)	94.9% (93.1%)
>50	TP	3,880 (0)	3,347 (3,126)	3,879 (0)	4,057 (1,899)	4,063 (4,001)	4,062 (4,008)	3,996 (3,812)	3,995 (3,901)	4,047 (3,946)	4,032 (3,899)	4,007 (2,629)	4,055 (3,897)
	FP	339	974	286	106	131	88	305	143	102	134	74	100
	FN	236	3,258	237	59	53	54	120	121	69	84	109	61
	Recall	94.3% (0%)	50.7% (49.0%)	94.2% (0%)	98.6% (97.0%)	98.7% (98.7%)	98.7% (98.7%)	97.1% (96.9%)	97.1% (97.0%)	98.3% (98.3%)	98.0% (97.9%)	97.4% (96.0%)	98.5% (98.5%)
	Precision	92.0% (0%)	77.5% (72.3%)	93.1% (0%)	97.5% (45.6%)	96.9% (95.4%)	97.9% (96.6%)	92.9% (88.6%)	96.5% (94.3%)	97.5% (95.1%)	96.8% (93.6%)	98.2% (64.4%)	97.6% (93.8%)
	F1	93.1% (N/A)	61.3% (58.4%)	93.7% (N/A)	98.0% (62.0%)	97.8% (97.0%)	98.3% (97.6%)	95.0% (92.6%)	96.8% (95.6%)	97.9% (96.7%)	97.4% (95.7%)	97.8% (77.1%)	98.1% (96.1%)

INS		PBHoney (2014)	NanoSV (2017)	Smartie-sv_aln (2018)	Sniffles (2018)	SVIM (2019)	cuteSV (2020)	NanoVar (2020)	pbsv (2021)	SKSV (2021)	Sniffles2 (2022)	MAMnet (2022)	DeBreak (2022)
Total Benchmark Calls (>50): 5281													
50˜1k	TP	3,563 (0)	4,395 (3,851)	4,171 (0)	4,018 (1,490)	3,889 (3,545)	4,218 (4,170)	2,892 (2,579)	3,468 (3,101)	4,423 (4,324)	4,268 (3,915)	4,216 (2,055)	4,456 (4,050)
	FP	326	2,014	545	199	271	204	618	311	248	558	167	671
	FN	998	1,429	390	543	672	343	1,669	1,093	138	293	345	105
	Recall	78.1% (0%)	75.5% (72.9%)	91.4% (0%)	88.1% (73.3%)	85.3% (84.1%)	92.5% (92.4%)	63.4% (60.7%)	76.0% (73.9%)	97.0% (96.9%)	93.6% (93.0%)	92.4% (85.6%)	97.7% (97.5%)
	Precision	91.6% (0%)	68.6% (60.1%)	88.4% (0%)	95.3% (35.3%)	93.5% (85.2%)	95.4% (94.3%)	82.4% (73.5%)	91.8% (82.1%)	94.7% (92.6%)	88.4% (81.1%)	96.2% (46.9%)	86.9% (79.0%)
	F1	84.3% (N/A)	71.9% (65.9%)	89.9% (N/A)	91.5% (47.7%)	89.2% (84.6%)	93.9% (93.3%)	71.7% (66.5%)	83.2% (77.8%)	95.8% (94.7%)	90.9% (86.7%)	94.3% (60.6%)	92.0% (87.3%)
1k˜10k	TP	29 (0)	361 (235)	187 (0)	230 (51)	163 (108)	548 (544)	219 (163)	664 (520)	642 (636)	300 (200)	528 (320)	582 (490)
	FP	322	110	17	3	4	11	68	98	32	20	19	16
	FN	667	498	509	466	533	148	477	32	54	396	168	114
	Recall	4.2% (0%)	42.0% (32.1%)	26.9% (0%)	33.0% (9.9%)	23.4% (16.8%)	78.7% (78.6%)	31.5% (25.5%)	95.4% (94.2%)	92.2% (92.2%)	43.1% (33.6%)	75.9% (65.6%)	83.6% (81.1%)
	Precision	8.3% (0%)	76.6% (49.9%)	91.7% (0%)	98.7% (21.9%)	97.6% (64.7%)	98.0% (97.3%)	76.3% (56.8%)	87.1% (68.2%)	95.3% (94.4%)	93.8% (62.5%)	96.5% (58.5%)	97.3% (81.9%)
	F1	5.5% (N/A)	54.3% (39.0%)	41.6% (N/A)	49.5% (13.6%)	37.8% (26.7%)	87.3% (87.0%)	44.6% (35.2%)	91.1% (79.1%)	93.7% (93.3%)	59.1% (43.7%)	85.0% (61.8%)	90.0% (81.5%)
>10k	TP	0 (0)	0 (0)	0 (0)	0 (0)	0 (0)	2 (2)	0 (0)	8 (4)	4 (4)	0 (0)	3 (3)	12 (2)
	FP	0	0	0	0	0	1	4	1	0	0	0	1
	FN	24	24	24	24	24	22	24	16	20	24	21	12
	Recall	0% (0%)	0% (0%)	0% (0%)	0% (0%)	0% (0%)	8.3% (8.3%)	0.0% (0%)	33.3% (20.0%)	16.7% (16.7%)	0% (0%)	12.5% (12.5%)	50.0% (14.3%)
	Precision	0% (0%)	0% (0%)	0% (0%)	0% (0%)	0% (0%)	66.7% (66.7%)	0.0% (0%)	88.9% (44.4%)	100.0% (100.0%)	0% (0%)	100.0% (100.0%)	92.3% (15.4%)
	F1	N/A (N/A)	N/A (N/A)	N/A (N/A)	N/A (N/A)	N/A (N/A)	14.8% (14.8%)	N/A (N/A)	48.5% (27.6%)	28.6% (28.6%)	N/A (N/A)	22.2% (22.2%)	64.9% (14.8%)
>50	TP	3,595 (0)	4,757 (4,087)	4,358 (0)	4,249 (1,541)	4,053 (3,653)	4,770 (4,718)	3,113 (2,741)	4,143 (3,628)	5,072 (4,967)	4,570 (4,116)	4,750 (2,380)	5,052 (4,544)
	FP	645	2,105	559	198	272	211	665	402	272	560	179	678
	FN	1,686	1,951	923	1,032	1,228	511	2,168	1,138	209	711	531	229
	Recall	68.1% (0%)	70.9% (67.7%)	82.5% (0%)	80.5% (59.9%)	76.7% (74.8%)	90.3% (90.2%)	58.9% (55.8%)	78.5% (76.1%)	96.0% (96.0%)	86.5% (85.3%)	89.9% (81.8%)	95.7% (95.2%)
	Precision	84.8% (0%)	69.3% (59.6%)	88.6% (0%)	95.5% (34.7%)	93.7% (84.5%)	95.8% (94.7%)	82.4% (72.6%)	91.2% (79.8%)	94.9% (92.9%)	89.1% (80.2%)	96.4% (48.3%)	88.2% (79.3%)
	F1	75.5% (N/A)	70.1% (63.4%)	85.5% (N/A)	87.4% (43.9%)	84.4% (79.4%)	93.0% (92.4%)	68.7% (63.1%)	84.3% (77.9%)	95.5% (94.4%)	87.8% (82.7%)	93.0% (60.7%)	91.8% (86.5%)

Supplementary Table 3: Genome-wide SV evaluation against GIAB benchmark on **Hifi_L1** (p=0, P=0.5, r=500, and O=0 for Truvari). The top and bottom panels are for deletion SVs and insertion SVs, respectively. SVs are categorized into different size ranges for evaluation: 50bp-1kb, 1kb-10kb, >10kb, and >50bp. The evaluation metrics are true positive (TP), false positive (FP), false negative (FN), recall, precision and F1. For precision, recall, and F1, the highest values across all tools are highlighted in green color. The evaluated **read alignment-based** SV callers are PBHoney, NanoSV, Smartie-sv_aln, Sniffles, SVIM, cuteSV, NanoVar, pbsv, SKSV, Sniffles2, MAMnet, and DeBreak shown in the table in chronological order by publication year. Genotyping accuracy results are highlighted in blue. The input reads alignment file for all read alignment-based SV callers is from NGMLR. Source data are provided as a Source Data file.

DEL		NanoSV (2017)	Smartie-sv_aln (2018)	Sniffles (2018)	SVIM (2019)	cuteSV (2020)	NanoVar (2020)	Sniffles2 (2022)	MAMnet (2022)	DeBreak (2022)
Total Benchmark Calls (>50): 4116										
50˜1k	TP	2,376 (2,210)	3,434 (0)	3,502 (1,633)	3,558 (3,483)	3,563 (3,516)	3,152 (2,930)	3,556 (3,520)	3,379 (2,032)	3,558 (3,401)
	FP	1,082	4,014	377	274	258	611	377	174	664
	FN	3,441	171	103	47	42	453	49	226	47
	Recall	40.8% (39.1%)	95.3% (0%)	97.1% (94.1%)	98.7% (98.7%)	98.8% (98.8%)	87.4% (86.6%)	98.6% (98.6%)	93.7% (90.0%)	98.7% (98.6%)
	Precision	68.7% (63.9%)	46.1% (0%)	90.3% (42.1%)	92.8% (90.9%)	93.2% (92.0%)	83.8% (77.9%)	90.4% (89.5%)	95.1% (57.2%)	84.3% (80.6%)
	F1	51.2% (48.5%)	62.1% (N/A)	93.6% (58.2%)	95.7% (94.6%)	96.0% (95.3%)	85.6% (82.0%)	94.3% (93.8%)	94.4% (69.9%)	90.9% (88.7%)
1k˜10k	TP	355 (333)	462 (0)	477 (172)	472 (457)	471 (462)	433 (403)	477 (473)	432 (394)	478 (470)
	FP	105	204	4	9	6	63	5	14	3
	FN	378	19	4	9	10	48	4	49	3
	Recall	48.4% (46.8%)	96.0% (0%)	99.2% (97.7%)	98.1% (98.1%)	97.9% (97.9%)	90.0% (89.4%)	99.2% (99.2%)	89.8% (88.9%)	99.4% (99.4%)
	Precision	77.2% (72.4%)	69.4% (0%)	99.2% (35.8%)	98.1% (95.0%)	98.7% (96.9%)	87.3% (81.2%)	99.0% (98.1%)	96.9% (88.3%)	99.4% (97.7%)
	F1	59.5% (56.9%)	80.6% (N/A)	99.2% (52.4%)	98.1% (96.5%)	98.3% (97.4%)	88.6% (85.1%)	99.1% (98.6%)	93.2% (88.6%)	99.4% (98.5%)
>10k	TP	21 (20)	16 (0)	29 (11)	24 (23)	29 (27)	25 (22)	26 (20)	15 (15)	26 (25)
	FP	1	1	0	0	0	5	0	5	0
	FN	21	13	0	5	0	4	3	14	3
	Recall	50.0% (48.8%)	55.2% (0%)	100.0% (100.0%)	82.8% (82.1%)	100.0% (100.0%)	86.2% (84.6%)	89.7% (87.0%)	51.7% (51.7%)	89.7% (89.3%)
	Precision	95.5% (90.9%)	94.1% (0%)	100.0% (37.9%)	100.0% (95.8%)	100.0% (93.1%)	83.3% (73.3%)	100.0% (76.9%)	75.0% (75.0%)	100.0% (96.2%)
	F1	65.6% (63.5%)	69.6% (N/A)	100.0% (55.0%)	90.6% (88.5%)	100.0% (96.4%)	84.7% (78.6%)	94.5% (81.6%)	61.2% (61.2%)	94.5% (92.6%)
>50	TP	2,754 (2,564)	3,913 (0)	4,010 (1,817)	4,055 (3,964)	4,064 (4,006)	3,622 (3,364)	4,060 (4,014)	3,836 (2,450)	4,063 (3,897)
	FP	1,186	4,215	376	280	261	665	378	183	665
	FN	3,851	203	106	61	52	494	56	280	53
	Recall	41.7% (40.0%)	95.1% (0%)	97.4% (94.5%)	98.5% (98.5%)	98.7% (98.7%)	88.0% (87.2%)	98.6% (98.6%)	93.2% (89.7%)	98.7% (98.7%)
	Precision	69.9% (65.1%)	48.1% (0%)	91.4% (41.4%)	93.5% (91.4%)	94.0% (92.6%)	84.5% (78.5%)	91.5% (90.4%)	95.4% (61.0%)	85.9% (82.4%)
	F1	52.2% (49.5%)	63.9% (N/A)	94.3% (57.6%)	96.0% (94.8%)	96.3% (95.6%)	86.2% (82.6%)	94.9% (94.4%)	94.3% (72.6%)	91.9% (89.8%)

INS		NanoSV (2017)	Smartie-sv_aln (2018)	Sniffles (2018)	SVIM (2019)	cuteSV (2020)	NanoVar (2020)	Sniffles2 (2022)	MAMnet (2022)	DeBreak (2022)
Total Benchmark Calls (>50): 5281										
50˜1k	TP	4,405 (3,785)	2,530 (0)	4,128 (1,439)	4,239 (3,503)	4,394 (4,314)	1,997 (1,782)	4,363 (4,246)	4,317 (2,331)	4,370 (4,010)
	FP	6,274	1,934	620	855	293	338	352	180	268
	FN	1,419	2,031	433	322	167	2,564	198	244	191
	Recall	75.6% (72.7%)	55.5% (0%)	90.5% (76.9%)	92.9% (91.6%)	96.3% (96.3%)	43.8% (41.0%)	95.7% (95.5%)	94.7% (90.5%)	95.8% (95.5%)
	Precision	41.2% (35.4%)	56.7% (0%)	86.9% (30.3%)	83.2% (68.8%)	93.7% (92.0%)	85.5% (76.3%)	92.5% (90.1%)	96.0% (51.8%)	94.2% (86.5%)
	F1	53.4% (47.7%)	56.1% (N/A)	88.7% (43.5%)	87.8% (78.6%)	95.0% (94.1%)	57.9% (53.3%)	94.1% (92.7%)	95.3% (65.9%)	95.0% (90.7%)
1k˜10k	TP	650 (499)	23 (0)	604 (106)	530 (384)	635 (621)	133 (103)	636 (514)	642 (415)	655 (587)
	FP	291	1	39	34	26	82	40	40	32
	FN	209	673	92	166	61	563	60	54	41
	Recall	75.7% (70.5%)	3.3% (0%)	86.8% (53.5%)	76.1% (69.8%)	91.2% (91.1%)	19.1% (15.5%)	91.4% (89.5%)	92.2% (88.5%)	94.1% (93.5%)
	Precision	69.1% (53.0%)	95.8% (0%)	93.9% (16.5%)	94.0% (68.1%)	96.1% (93.9%)	61.9% (47.9%)	94.1% (76.0%)	94.1% (60.9%)	95.3% (85.4%)
	F1	72.2% (60.5%)	6.4% (N/A)	90.2% (25.2%)	84.1% (68.9%)	93.6% (92.5%)	29.2% (23.4%)	92.7% (82.2%)	93.2% (72.1%)	94.7% (89.3%)
>10k	TP	13 (7)	0 (0)	11 (0)	10 (2)	16 (13)	0 (0)	13 (12)	15 (14)	18 (10)
	FP	4	0	4	2	2	5	7	3	2
	FN	11	24	13	14	8	24	11	9	6
	Recall	54.2% (38.9%)	0% (0%)	45.8% (0%)	41.7% (12.5%)	66.7% (61.9%)	0.0% (0%)	54.2% (52.2%)	62.5% (60.9%)	75.0% (62.5%)
	Precision	76.5% (41.2%)	0% (0%)	73.3% (0%)	83.3% (16.7%)	88.9% (72.2%)	0.0% (0%)	65.0% (60.0%)	83.3% (77.8%)	90.0% (50.0%)
	F1	63.4% (40.0%)	N/A (N/A)	56.4% (N/A)	55.6% (14.3%)	76.2% (66.7%)	N/A (N/A)	59.1% (55.8%)	71.4% (68.3%)	81.8% (55.6%)
>50	TP	5,070 (4,288)	2,553 (0)	4,743 (1,545)	4,780 (3,889)	5,046 (4,949)	2,135 (1,884)	5,013 (4,773)	4,976 (2,762)	5,044 (4,608)
	FP	6,516	1,930	628	857	266	402	362	193	268
	FN	1,638	2,728	538	501	235	3,146	268	305	237
	Recall	75.6% (72.4%)	48.3% (0%)	89.8% (74.2%)	90.5% (88.6%)	95.6% (95.5%)	40.4% (37.5%)	94.9% (94.7%)	94.2% (90.1%)	95.5% (95.1%)
	Precision	43.8% (37.0%)	56.9% (0%)	88.3% (28.8%)	84.8% (69.0%)	95.0% (93.2%)	84.2% (74.3%)	93.3% (88.8%)	96.3% (53.4%)	95.0% (86.7%)
	F1	55.4% (49.0%)	52.3% (N/A)	89.1% (41.5%)	87.6% (77.6%)	95.3% (94.3%)	54.6% (49.8%)	94.1% (91.6%)	95.2% (67.1%)	95.2% (90.7%)

Supplementary Table 4: Genome-wide SV evaluation against GIAB benchmark on **Nano_L1** (p=0, P=0.5, r=500, and O=0 for Truvari). The top and bottom panels are for deletion SVs and insertion SVs, respectively. SVs are categorized into different size ranges for evaluation: 50bp-1kb, 1kb-10kb, >10kb, and >50bp. The evaluation metrics are true positive (TP), false positive (FP), false negative (FN), recall, precision, and F1. For precision, recall and F1, the highest values across all tools are highlighted in green color. The evaluated **read alignment-based** SV callers are NanoSV, Smartie-sv_aln, Sniffles, SVIM, cuteSV, NanoVar, Sniffles2, MAMnet and DeBreak shown in the table in chronological order by publication year. Genotyping accuracy results are highlighted in blue. The input reads alignment file for all read alignment-based SV callers is from NGMLR. Source data are provided as a Source Data file.

Hifi_L1

DEL		Dipcall (2018)	Smartie-sv_asm (2018)	SVIM-asm (2020)	PAV (2021)
		Total Benchmark Calls (>50): 4116			
50˜1k	TP	3,437 (3,321)	3,530 (3,384)	3,532 (3,473)	3,554 (3,499)
	FP	241	287	184	163
	FN	168	75	73	51
	Recall	95.3% (95.2%)	97.9% (97.8%)	98.0% (97.9%)	98.6% (98.6%)
	Precision	93.4% (90.3%)	92.5% (88.7%)	95.0% (93.5%)	95.6% (94.1%)
	F1	94.4% (92.7%)	95.1% (93.0%)	96.5% (95.6%)	97.1% (96.3%)
1k˜10k	TP	458 (450)	478 (467)	471 (465)	478 (469)
	FP	8	7	4	3
	FN	23	3	10	3
	Recall	95.2% (95.1%)	99.4% (99.4%)	97.9% (97.9%)	99.4% (99.4%)
	Precision	98.3% (96.6%)	98.6% (96.3%)	99.2% (97.9%)	99.4% (97.5%)
	F1	96.7% (95.8%)	99.0% (97.8%)	98.5% (97.9%)	99.4% (98.4%)
>10k	TP	27 (26)	18 (18)	28 (27)	28 (27)
	FP	1	1	1	1
	FN	2	11	1	1
	Recall	93.1% (92.9%)	62.1% (62.1%)	96.6% (96.4%)	96.6% (96.4%)
	Precision	96.4% (92.9%)	94.7% (94.7%)	96.6% (93.1%)	96.6% (93.1%)
	F1	94.7% (92.9%)	75.0% (75.0%)	96.6% (94.7%)	96.6% (94.7%)
>50	TP	3,923 (3,798)	4,027 (3,870)	4,032 (3,965)	4,061 (3,996)
	FP	248	289	186	161
	FN	193	89	84	55
	Recall	95.3% (95.2%)	97.8% (97.8%)	98.0% (97.9%)	98.7% (98.6%)
	Precision	94.1% (91.1%)	93.3% (89.7%)	95.6% (94.0%)	96.2% (94.6%)
	F1	94.7% (93.1%)	95.5% (93.5%)	96.8% (95.9%)	97.4% (96.6%)

INS		Dipcall (2018)	Smartie-sv_asm (2018)	SVIM-asm (2020)	PAV (2021)
		Total Benchmark Calls (>50): 5281			
50˜1k	TP	4,267 (4,020)	4,437 (4,197)	4,408 (4,333)	4,449 (4,383)
	FP	617	760	540	461
	FN	294	124	153	112
	Recall	93.6% (93.2%)	97.3% (97.1%)	96.6% (96.6%)	97.5% (97.5%)
	Precision	87.4% (82.3%)	85.4% (80.8%)	89.1% (87.6%)	90.6% (89.3%)
	F1	90.4% (87.4%)	90.9% (88.2%)	92.7% (91.9%)	93.9% (93.2%)
1k˜10k	TP	621 (596)	658 (623)	641 (593)	657 (646)
	FP	52	63	66	45
	FN	75	38	55	39
	Recall	89.2% (88.8%)	94.5% (94.3%)	92.1% (91.5%)	94.4% (94.3%)
	Precision	92.3% (88.6%)	91.3% (86.4%)	90.7% (83.9%)	93.6% (92.0%)
	F1	90.7% (88.7%)	92.9% (90.2%)	91.4% (87.5%)	94.0% (93.2%)
>10k	TP	16 (16)	11 (11)	16 (14)	16 (16)
	FP	6	8	10	9
	FN	8	13	8	8
	Recall	66.7% (66.7%)	45.8% (45.8%)	66.7% (63.6%)	66.7% (66.7%)
	Precision	72.7% (72.7%)	57.9% (57.9%)	61.5% (53.8%)	64.0% (64.0%)
	F1	69.6% (69.6%)	51.2% (51.2%)	64.0% (58.3%)	65.3% (65.3%)
>50	TP	4,908 (4,636)	5,109 (4,834)	5,068 (4,942)	5,126 (5,049)
	FP	663	818	605	505
	FN	373	172	213	155
	Recall	92.9% (92.6%)	96.7% (96.6%)	96.0% (95.9%)	97.1% (97.0%)
	Precision	88.1% (83.2%)	86.2% (81.6%)	89.3% (87.1%)	91.0% (89.7%)
	F1	90.5% (87.6%)	91.2% (88.4%)	92.5% (91.3%)	94.0% (93.2%)

Nano_L1

DEL		Dipcall (2018)	Smartie-sv_asm (2018)	SVIM-asm (2020)	PAV (2021)
		Total Benchmark Calls (>50): 4116			
50˜1k	TP	3,211 (2,887)	N/A (N/A)	3,502 (3,433)	3,562 (3,505)
	FP	428	N/A	209	187
	FN	394	N/A	103	43
	Recall	89.1% (88.0%)	N/A (N/A)	97.1% (97.1%)	98.8% (98.8%)
	Precision	88.2% (79.3%)	N/A (N/A)	94.4% (92.5%)	95.0% (93.5%)
	F1	88.7% (83.4%)	N/A (N/A)	95.7% (94.7%)	96.9% (96.1%)
1k˜10k	TP	443 (421)	N/A (N/A)	469 (462)	478 (468)
	FP	29	N/A	6	4
	FN	38	N/A	12	3
	Recall	92.1% (91.7%)	N/A (N/A)	97.5% (97.5%)	99.4% (99.4%)
	Precision	93.9% (89.2%)	N/A (N/A)	98.7% (97.3%)	99.2% (97.1%)
	F1	93.0% (90.4%)	N/A (N/A)	98.1% (97.4%)	99.3% (98.2%)
>10k	TP	21 (16)	N/A (N/A)	23 (19)	24 (20)
	FP	0	N/A	0	0
	FN	8	N/A	6	5
	Recall	72.4% (66.7%)	N/A (N/A)	79.3% (76.0%)	82.8% (80.0%)
	Precision	100.0% (76.2%)	N/A (N/A)	100.0% (82.6%)	100.0% (83.3%)
	F1	84.0% (71.1%)	N/A (N/A)	88.5% (79.2%)	90.6% (81.6%)
>50	TP	3,675 (3,324)	N/A (N/A)	3,995 (3,914)	4,065 (3,994)
	FP	456	N/A	212	187
	FN	441	N/A	121	51
	Recall	89.3% (88.3%)	N/A (N/A)	97.1% (97.0%)	98.8% (98.7%)
	Precision	89.0% (80.5%)	N/A (N/A)	95.0% (93.0%)	95.6% (93.9%)
	F1	89.1% (84.2%)	N/A (N/A)	96.0% (95.0%)	97.2% (96.3%)

INS		Dipcall (2018)	Smartie-sv_asm (2018)	SVIM-asm (2020)	PAV (2021)
		Total Benchmark Calls (>50): 5281			
50˜1k	TP	3,964 (3,251)	N/A (N/A)	4,299 (4,173)	4,436 (4,388)
	FP	958	N/A	582	479
	FN	597	N/A	262	125
	Recall	86.9% (84.5%)	N/A (N/A)	94.3% (94.1%)	97.3% (97.2%)
	Precision	80.5% (66.1%)	N/A (N/A)	88.1% (85.5%)	90.3% (89.3%)
	F1	83.6% (74.1%)	N/A (N/A)	91.1% (89.6%)	93.6% (93.1%)
1k˜10k	TP	590 (511)	N/A (N/A)	627 (585)	650 (644)
	FP	82	N/A	65	48
	FN	106	N/A	69	46
	Recall	84.8% (82.8%)	N/A (N/A)	90.1% (89.4%)	93.4% (93.3%)
	Precision	87.8% (76.0%)	N/A (N/A)	90.6% (84.5%)	93.1% (92.3%)
	F1	86.3% (79.3%)	N/A (N/A)	90.3% (86.9%)	93.3% (92.8%)
>10k	TP	14 (13)	N/A (N/A)	16 (10)	16 (16)
	FP	7	N/A	11	4
	FN	10	N/A	8	8
	Recall	58.3% (56.5%)	N/A (N/A)	66.7% (55.6%)	66.7% (66.7%)
	Precision	66.7% (61.9%)	N/A (N/A)	59.3% (37.0%)	80.0% (80.0%)
	F1	62.2% (59.1%)	N/A (N/A)	62.7% (44.4%)	72.7% (72.7%)
>50	TP	4,570 (3,777)	N/A (N/A)	4,944 (4,770)	5,105 (5,051)
	FP	1,030	N/A	637	512
	FN	711	N/A	337	176
	Recall	86.5% (84.2%)	N/A (N/A)	93.6% (93.4%)	96.7% (96.6%)
	Precision	81.6% (67.4%)	N/A (N/A)	88.6% (85.5%)	90.9% (89.9%)
	F1	84.0% (74.9%)	N/A (N/A)	91.0% (89.3%)	93.7% (93.2%)

Supplementary Table 5: Genome-wide SV evaluation against GIAB benchmark on **Hifi_L1** (top panel) and **Nano_L1** (bottom panel) (p=0, P=0.5, r=500, and O=0 for Truvari). SVs are categorized into different size ranges for evaluation: 50bp-1kb, 1kb-10kb, >10kb, and >50bp. The evaluation metrics are true positive (TP), false positive (FP), false negative (FN), recall, precision and F1. For precision, recall, and F1, the highest values across all tools are highlighted in green color. The evaluated **assembly-based** SV callers are Dipcall, Smartie-sv_asm, SVIM-asm, and PAV shown in the table in chronological order by publication year. Genotyping accuracy results are highlighted in blue. The input assembly contig file for all assembly-based SV callers is from hifiasm (for Hifi_L1) and Flye + HapDup (for Nano_L1). Source data are provided as a Source Data file.

Read read alignment-based								
	**Hifi_L1**				**Nano_L1**			
SV Caller	Run Time	CPU Cores	CPU hours	Memory(Gb)	Run Time	CPU Cores	CPU hours	Memory(Gb)
PBHoney	01:25:19	10	14	33	N/A			
NanoSV	3-14:21:44	10	863	37	20:27:37	10	204	12
Smartie-sv_aln	03:21:36	5	17	5	1-02:12:36	15	393	153
Sniffles	01:43:50	10	17	4	00:59:15	10	10	1
SVIM	00:45:37	10	8	1	01:27:37	10	15	2
cuteSV	00:19:36	10	3	3	00:30:31	10	5	2
NanoVar	06:24:35	10	64	56	04:44:07	20	95	59
pbsv	04:13:52	10	42	23	N/A			
SKSV	05:41:17	10	57	70	N/A			
DeBreak	22:25:34	20	448	55	2-08:53:18	15	853	83
Sniffles2	00:49:57	20	17	4	02:11:32	20	44	7
MAMnet	00:37:29	6	4	28	02:22:54	6	14	31
**Assembly-based**								
	**Hifi_L1**				**Nano_L1**			
SV Caller	Run Time	CPU Cores	CPU hours	Memory(Gb)	Run Time	CPU Cores	CPU hours	Memory(Gb)
Dipcall	00:47:49	20	16	46	01:11:44	20	24	47
Smartie-sv_asm	08:01:21	20	160	48	N/A			
SVIM-asm	01:48:48	20	36	26	01:06:32	20	22	38
PAV	05:47:20	20	116	56	5-17:58:19	20	2760	128

Node Information		
**CPU**	**RAM**	**GPU**
AMD EPYC 7452 32-Core Processor	1007G	NVIDIA GeForce RTX 2080 Ti

Supplementary Table 6: Computing resource consumption for different read alignment-based (top panel) and assembly-based SV callers (bottom panel) on **Hifi_L1** and **Nano_L1**.

Aligner								
	**Hifi_L1**				**Nano_L1**			
Aligner	Run Time	CPU Cores	CPU hours	Memory(Gb)	Run Time	CPU Cores	CPU hours	Memory(Gb)
NGMLR	1-05:17:51	10	293	13	09:42:13	10	97	11
minimap2	10:00:52	30	189	31	19:45:57	30	593	33
Winnowmap	08:41:52	20	174	43	20:01:55	20	400	17
LRA	14:41:23	20	294	24	16:11:24	20	324	23
**Assembler**								
	**Hifi_L1**				**Nano_L1**			
Assembler	Run Time	CPU Cores	CPU hours	Memory(Gb)	Run Time	CPU Cores	CPU hours	Memory(Gb)
Hifiasm	21:44:01	20	440	214	N/A			
HiCanu +	3-20:09:00	32	2949	235	N/A			
purge_dups	07:26:42	32	238	26	N/A			
Peregrine +	4-11:43:25	50	5386	377	N/A			
HapDup	1-02:40:54	64	1708	66	N/A			
Flye +	3-22:44:56	20	1895	263	1-11:02:34	20	701	221
HapDup	1-05:47:53	64	1907	82	15:59:44	64	1024	52
IPA	01-23:00:00	100	4717	88	N/A			
wtdbg2 +	1-13:50:21	20	757	187	3-00:17:45	20	1446	313
HapDup	18:35:32	64	1190	72	1-12:45:11	64	2352	58
Shasta +	N/A				09:57:46	60	598	377
HapDup	N/A				20:08:41	64	1289	58

Node Information		
**CPU**	**RAM**	**GPU**
AMD EPYC 7452 32-Core Processor	1007G	NVIDIA GeForce RTX 2080 Ti

Supplementary Table 7: Computing resource consumption for different aligners (top panel) and assemblers (bottom panel) on **Hifi_L1** and **Nano_L1**.

	DEL	PBHoney (2014)	NanoSV (2017)	Smartie-sv_aln (2018)	Sniffles (2018)	SVIM (2019)	cuteSV (2020)	NanoVar (2020)	pbsv (2021)	SKSV (2021)	Sniffles2 (2022)	MAMnet (2022)	DeBreak (2022)
		Total Benchmark Calls (>50): 4116											
5x	TP	3,631 (0)	1,961 (1,571)	3,049 (0)	55 (1)	54 (54)	59 (59)	3,035 (2,526)	3,780 (3,385)	54 (54)	3,144 (2,792)	3,743 (2,338)	1,803 (1,412)
	FP	150	51	141	1	1	2	203	199	1	94	104	17
	FN	485	4,644	1,067	4,061	4,062	4,057	1,081	336	4,062	972	373	2,313
	Recall	88.2% (0%)	29.7% (25.3%)	74.1% (0%)	1.3% (0.0%)	1.3% (1.3%)	1.4% (1.4%)	73.7% (70.0%)	91.8% (91.0%)	1.3% (1.3%)	76.4% (74.2%)	90.9% (86.2%)	43.8% (37.9%)
	Precision	96.0% (0%)	97.5% (78.1%)	95.6% (0%)	98.2% (1.8%)	98.2% (98.2%)	96.7% (96.7%)	93.7% (78.0%)	95.0% (85.1%)	98.2% (98.2%)	97.1% (86.2%)	97.3% (60.8%)	99.1% (77.6%)
	F1	92.0% (N/A)	45.5% (38.2%)	83.5% (N/A)	2.6% (0.0%)	2.6% (2.6%)	2.8% (2.8%)	82.5% (73.8%)	93.4% (87.9%)	2.6% (2.6%)	85.5% (79.7%)	94.0% (71.3%)	60.7% (50.9%)
10x	TP	3,981 (0)	2,597 (2,339)	3,870 (0)	865 (86)	916 (888)	944 (898)	3,580 (3,263)	3,971 (3,819)	907 (863)	3,848 (3,657)	3,990 (2,849)	3,345 (2,845)
	FP	220	195	395	9	9	13	304	184	9	149	138	52
	FN	135	4,008	246	3,251	3,200	3,172	536	145	3,209	268	126	771
	Recall	96.7% (0%)	39.3% (36.9%)	94.0% (0%)	21.0% (2.6%)	22.3% (21.7%)	22.9% (22.1%)	87.0% (85.9%)	96.5% (96.3%)	22.0% (21.2%)	93.5% (93.2%)	96.9% (95.8%)	81.3% (78.7%)
	Precision	94.8% (0%)	93.0% (83.8%)	90.7% (0%)	99.0% (9.8%)	99.0% (96.0%)	98.6% (93.8%)	92.2% (84.0%)	95.6% (91.9%)	99.0% (94.2%)	96.3% (91.5%)	96.7% (69.0%)	98.5% (83.8%)
	F1	95.7% (N/A)	55.3% (51.2%)	92.4% (N/A)	34.7% (4.1%)	36.3% (35.4%)	37.2% (35.7%)	89.5% (84.9%)	96.0% (94.1%)	36.0% (34.6%)	94.9% (92.3%)	96.8% (80.2%)	89.0% (81.1%)
20x	TP	4,014 (0)	2,956 (2,780)	4,005 (0)	2,781 (1,094)	2,846 (2,800)	2,875 (2,761)	3,835 (3,636)	4,016 (3,949)	2,826 (2,707)	4,041 (3,963)	3,967 (2,413)	4,005 (3,627)
	FP	334	462	380	52	58	52	325	158	45	158	85	94
	FN	102	3,649	111	1,335	1,270	1,241	281	100	1,290	75	149	111
	Recall	97.5% (0%)	44.8% (43.2%)	97.3% (0%)	67.6% (45.0%)	69.1% (68.8%)	69.8% (69.0%)	93.2% (92.8%)	97.6% (97.5%)	68.7% (67.7%)	98.2% (98.1%)	96.4% (94.2%)	97.3% (97.0%)
	Precision	92.3% (0%)	86.5% (81.3%)	91.3% (0%)	98.2% (38.6%)	98.0% (96.4%)	98.2% (94.3%)	92.2% (87.4%)	96.2% (94.6%)	98.4% (94.3%)	96.2% (94.4%)	97.9% (59.6%)	97.7% (88.5%)
	F1	94.8% (N/A)	59.0% (56.5%)	94.2% (N/A)	80.0% (41.6%)	81.1% (80.3%)	81.6% (79.7%)	92.7% (90.0%)	96.9% (96.0%)	80.9% (78.8%)	97.2% (96.2%)	97.1% (73.0%)	97.5% (92.6%)
30x	TP	4,013 (0)	3,121 (2,960)	4,011 (0)	3,770 (1,587)	3,811 (3,757)	3,826 (3,742)	3,946 (3,749)	4,013 (3,961)	3,782 (3,669)	4,059 (4,002)	4,013 (3,895)	4,042 (3,806)
	FP	402	679	292	86	98	74	317	166	79	151	85	90
	FN	103	3,484	105	346	305	290	170	103	334	57	103	74
	Recall	97.5% (0%)	47.3% (45.9%)	97.4% (0%)	91.6% (82.1%)	92.6% (92.5%)	93.0% (92.8%)	95.9% (95.7%)	97.5% (97.5%)	91.9% (91.7%)	98.6% (98.6%)	97.5% (97.4%)	98.2% (98.1%)
	Precision	90.9% (0%)	82.1% (77.9%)	93.2% (0%)	97.8% (41.2%)	97.5% (96.1%)	98.1% (95.9%)	92.6% (87.9%)	96.0% (94.8%)	98.0% (95.0%)	96.4% (95.1%)	97.9% (95.0%)	97.8% (92.1%)
	F1	94.1% (N/A)	60.0% (57.8%)	95.3% (N/A)	94.6% (54.8%)	95.0% (94.3%)	95.5% (94.4%)	94.2% (91.6%)	96.8% (96.1%)	94.8% (93.3%)	97.5% (96.8%)	97.7% (96.2%)	98.0% (95.0%)
40x	TP	4,011 (0)	3,407 (3,248)	4,028 (0)	4,008 (1,690)	4,024 (3,972)	4,029 (3,968)	3,997 (3,808)	4,016 (3,970)	3,998 (3,889)	4,070 (4,015)	3,990 (3,195)	4,052 (3,844)
	FP	465	883	304	115	133	83	330	169	93	149	74	95
	FN	105	3,198	88	108	92	87	119	100	118	46	126	64
	Recall	97.4% (0%)	51.6% (50.4%)	97.9% (0%)	97.4% (94.0%)	97.8% (97.7%)	97.9% (97.9%)	97.1% (97.0%)	97.6% (97.5%)	97.1% (97.1%)	98.9% (98.9%)	96.9% (96.2%)	98.4% (98.4%)
	Precision	89.6% (0%)	79.4% (75.7%)	93.0% (0%)	97.2% (41.0%)	96.8% (95.5%)	98.0% (96.5%)	92.4% (88.0%)	96.0% (94.9%)	97.7% (95.1%)	96.5% (95.2%)	98.2% (78.6%)	97.7% (92.7%)
	F1	93.4% (N/A)	62.5% (60.5%)	95.4% (N/A)	97.3% (57.1%)	97.3% (96.6%)	97.9% (97.2%)	94.7% (92.3%)	96.8% (96.2%)	97.4% (96.0%)	97.7% (97.0%)	97.6% (86.5%)	98.1% (95.4%)
50x	TP	4,006 (0)	3,479 (3,313)	4,034 (0)	4,052 (1,740)	4,059 (3,995)	4,062 (4,005)	4,011 (3,860)	4,018 (3,972)	4,044 (3,946)	4,073 (4,020)	4,019 (3,075)	4,051 (3,895)
	FP	524	945	371	130	158	85	330	161	100	152	74	122
	FN	110	3,126	82	64	57	54	105	98	72	43	97	65
	Recall	97.3% (0%)	52.7% (51.5%)	98.0% (0%)	98.4% (96.5%)	98.6% (98.6%)	98.7% (98.7%)	97.4% (97.4%)	97.6% (97.6%)	98.3% (98.2%)	99.0% (98.9%)	97.6% (96.9%)	98.4% (98.4%)
	Precision	88.4% (0%)	78.6% (74.9%)	91.6% (0%)	96.9% (41.6%)	96.3% (94.7%)	98.0% (96.6%)	92.4% (88.9%)	96.1% (95.0%)	97.6% (95.2%)	96.4% (95.1%)	98.2% (75.1%)	97.1% (93.3%)
	F1	92.7% (N/A)	63.1% (61.0%)	94.7% (N/A)	97.7% (58.1%)	97.4% (96.6%)	98.3% (97.6%)	94.9% (92.9%)	96.9% (96.3%)	97.9% (96.7%)	97.7% (97.0%)	97.9% (84.7%)	97.7% (95.8%)

	INS	PBHoney (2014)	NanoSV (2017)	Smartie-sv_aln (2018)	Sniffles (2018)	SVIM (2019)	cuteSV (2020)	NanoVar (2020)	pbsv (2021)	SKSV (2021)	Sniffles2 (2022)	MAMnet (2022)	DeBreak (2022)
		Total Benchmark Calls (>50): 5281											
5x	TP	2,681 (0)	3,836 (3,305)	3,716 (0)	45 (0)	46 (46)	49 (48)	2,981 (2,512)	3,879 (3,534)	43 (42)	3,990 (3,588)	4,376 (2,432)	2,157 (1,707)
	FP	572	390	384	0	0	1	649	396	2	312	213	71
	FN	2,600	2,872	1,565	5,236	5,235	5,232	2,300	1,402	5,238	1,291	905	3,124
	Recall	50.8% (0%)	57.2% (53.5%)	70.4% (0%)	0.9% (0%)	0.9% (0.9%)	0.9% (0.9%)	56.4% (52.2%)	73.5% (71.6%)	0.8% (0.8%)	75.6% (73.5%)	82.9% (72.9%)	40.8% (35.3%)
	Precision	82.4% (0%)	90.8% (78.2%)	90.6% (0%)	100.0% (0%)	100.0% (100.0%)	98.0% (96.0%)	82.1% (69.2%)	90.7% (82.7%)	95.6% (93.3%)	92.7% (83.4%)	95.4% (53.0%)	96.8% (76.6%)
	F1	62.8% (N/A)	70.2% (63.5%)	79.2% (N/A)	1.7% (N/A)	1.7% (1.7%)	1.8% (1.8%)	66.9% (59.5%)	81.2% (76.7%)	1.6% (1.6%)	83.3% (78.2%)	88.7% (61.4%)	57.5% (48.4%)
10x	TP	3,514 (0)	4,863 (4,414)	4,756 (0)	980 (81)	1,014 (990)	1,073 (1,012)	3,405 (3,055)	4,103 (3,868)	1,057 (1,011)	4,799 (4,513)	4,815 (2,827)	4,090 (3,290)
	FP	869	862	911	26	31	35	853	455	22	444	261	195
	FN	1,767	1,845	525	4,301	4,267	4,208	1,876	1,178	4,224	482	466	1,191
	Recall	66.5% (0%)	72.5% (70.5%)	90.1% (0%)	18.6% (1.8%)	19.2% (18.8%)	20.3% (19.4%)	64.5% (62.0%)	77.7% (76.7%)	20.0% (19.3%)	90.9% (90.4%)	91.2% (85.8%)	77.4% (73.4%)
	Precision	80.2% (0%)	84.9% (77.1%)	83.9% (0%)	97.4% (8.1%)	97.0% (94.7%)	96.8% (91.3%)	80.0% (71.7%)	90.0% (84.9%)	98.0% (93.7%)	91.5% (86.1%)	94.9% (55.7%)	95.4% (76.8%)
	F1	72.7% (N/A)	78.2% (73.7%)	86.9% (N/A)	31.2% (3.0%)	32.1% (31.4%)	33.6% (32.0%)	71.4% (66.5%)	83.4% (80.5%)	33.2% (32.0%)	91.2% (88.2%)	93.0% (67.6%)	85.5% (75.1%)
20x	TP	3,722 (0)	5,150 (4,805)	4,965 (0)	3,360 (990)	3,414 (3,310)	3,521 (3,385)	3,469 (3,214)	4,142 (3,986)	3,498 (3,370)	5,039 (4,853)	4,848 (4,490)	4,985 (4,317)
	FP	1,050	1,478	976	174	209	153	860	412	135	471	203	302
	FN	1,559	1,558	316	1,921	1,867	1,760	1,812	1,139	1,783	242	433	296
	Recall	70.5% (0%)	76.8% (75.5%)	94.0% (0%)	63.6% (34.0%)	64.6% (63.9%)	66.7% (65.8%)	65.7% (63.9%)	78.4% (77.8%)	66.2% (65.4%)	95.4% (95.3%)	91.8% (91.2%)	94.4% (93.6%)
	Precision	78.0% (0%)	77.7% (72.5%)	83.6% (0%)	95.1% (28.0%)	94.2% (91.4%)	95.8% (92.1%)	80.1% (74.2%)	91.0% (87.5%)	96.3% (92.8%)	91.5% (88.1%)	96.0% (88.9%)	94.3% (81.7%)
	F1	74.0% (N/A)	77.2% (74.0%)	88.5% (N/A)	76.2% (30.7%)	76.7% (75.2%)	78.6% (76.8%)	72.2% (68.7%)	84.2% (82.4%)	78.5% (76.7%)	93.4% (91.5%)	93.8% (90.0%)	94.3% (87.2%)
30x	TP	3,731 (0)	5,185 (4,829)	4,958 (0)	4,531 (1,508)	4,550 (4,421)	4,646 (4,544)	3,472 (3,223)	4,124 (3,986)	3,673 (3,579)	5,101 (4,942)	4,936 (3,679)	5,081 (4,563)
	FP	1,116	1,904	771	272	350	219	856	411	173	461	220	325
	FN	1,550	1,523	323	750	731	635	1,809	1,157	1,608	180	345	200
	Recall	70.6% (0%)	77.3% (76.0%)	93.9% (0%)	85.8% (66.8%)	86.2% (85.8%)	88.0% (87.7%)	65.7% (64.1%)	78.1% (77.5%)	69.6% (69.0%)	96.6% (96.5%)	93.5% (91.4%)	96.2% (95.8%)
	Precision	77.0% (0%)	73.1% (68.1%)	86.5% (0%)	94.3% (31.4%)	92.9% (90.2%)	95.5% (93.4%)	80.2% (74.5%)	90.9% (87.9%)	95.5% (93.1%)	91.7% (88.9%)	95.7% (71.4%)	94.0% (84.4%)
	F1	73.7% (N/A)	75.2% (71.9%)	90.1% (N/A)	89.9% (42.7%)	89.4% (88.0%)	91.6% (90.5%)	72.3% (68.9%)	84.0% (82.4%)	80.5% (79.2%)	94.1% (92.5%)	94.6% (80.2%)	95.1% (89.7%)
40x	TP	3,738 (0)	5,198 (4,841)	4,998 (0)	4,890 (1,680)	4,882 (4,750)	4,970 (4,899)	3,399 (3,175)	4,119 (3,985)	3,865 (3,787)	5,104 (4,945)	4,867 (2,876)	5,099 (4,668)
	FP	1,151	2,097	820	351	447	261	850	411	183	462	193	324
	FN	1,543	1,510	283	391	399	311	1,882	1,162	1,416	177	414	182
	Recall	70.8% (0%)	77.5% (76.2%)	94.6% (0%)	92.6% (81.1%)	92.4% (92.3%)	94.1% (94.0%)	64.4% (62.8%)	78.0% (77.4%)	73.2% (72.8%)	96.6% (96.5%)	92.2% (87.4%)	96.6% (96.2%)
	Precision	76.5% (0%)	71.3% (66.4%)	85.9% (0%)	93.3% (32.1%)	91.6% (89.1%)	95.0% (93.7%)	80.0% (74.7%)	90.9% (88.0%)	95.5% (93.6%)	91.7% (88.8%)	96.2% (56.8%)	94.0% (86.1%)
	F1	73.5% (N/A)	74.2% (71.0%)	90.1% (N/A)	92.9% (46.0%)	92.0% (90.7%)	94.6% (93.8%)	71.3% (68.2%)	84.0% (82.4%)	82.9% (81.9%)	94.1% (92.5%)	94.1% (68.9%)	95.3% (90.9%)
50x	TP	3,743 (0)	5,199 (4,838)	4,681 (0)	4,981 (1,742)	4,951 (4,773)	5,050 (4,986)	3,333 (3,139)	4,121 (3,992)	5,061 (4,962)	5,105 (4,960)	4,944 (4,121)	5,084 (4,726)
	FP	1,168	2,268	1,039	384	518	275	830	390	267	454	209	327
	FN	1,538	1,509	600	300	330	231	1,948	1,160	220	176	337	197
	Recall	70.9% (0%)	77.5% (76.2%)	88.6% (0%)	94.3% (85.3%)	93.8% (93.5%)	95.6% (95.6%)	63.1% (61.7%)	78.0% (77.5%)	95.8% (95.8%)	96.7% (96.6%)	93.6% (92.4%)	96.3% (96.0%)
	Precision	76.2% (0%)	69.6% (64.8%)	81.8% (0%)	92.8% (32.5%)	90.5% (87.3%)	94.8% (93.6%)	80.1% (75.4%)	91.4% (88.5%)	95.0% (93.1%)	91.8% (89.2%)	95.9% (80.0%)	94.0% (87.3%)
	F1	73.4% (N/A)	73.4% (70.0%)	85.1% (N/A)	93.6% (47.0%)	92.1% (90.3%)	95.2% (94.6%)	70.6% (67.9%)	84.2% (82.6%)	95.4% (94.4%)	94.2% (92.8%)	94.8% (85.8%)	95.1% (91.5%)

Supplementary Table 8: Genome-wide SV (>= 50bp) evaluation against GIAB benchmark on **subsampled Hifi_L1** datasets which are at 5x, 10x, 20x, 30x, 40x, and 50x (p=0, P=0.5, r=500, and O=0 for Truvari). The top and bottom panels are for deletion SVs and insertion SVs, respectively. The evaluation metrics are true positive (TP), false positive (FP), false negative (FN), recall, precision, and F1. For precision, recall, and F1, the highest values across all tools are highlighted in green color. The evaluated **read alignment-based** SV callers are PBHoney, NanoSV, Smartie-sv_aln, Sniffles, SVIM, cuteSV, NanoVar, pbsv, SKSV, Sniffles2, MAMnet, and DeBreak shown in the table in chronological order by publication year. Genotyping accuracy results are highlighted in blue. Source data are provided as a Source Data file.

	DEL	NanoSV (2017)	Smartie-sv_aln (2018)	Sniffles (2018)	SVIM (2019)	cuteSV (2020)	NanoVar (2020)	Sniffles2 (2022)	MAMnet (2022)	DeBreak (2022)
		Total Benchmark Calls (>50): 4116								
5x	**TP**	1,267 (1,070)	2,316 (0)	33 (1)	33 (33)	38 (36)	2,685 (2,187)	3,081 (2,741)	N/A (N/A)	1,564 (1,257)
	**FP**	121	431	0	0	0	458	395	N/A	38
	**FN**	5,338	1,800	4,083	4,083	4,078	1,431	1,035	N/A	2,552
	**Recall**	19.2% (16.7%)	56.3% (0%)	0.8% (0.0%)	0.8% (0.8%)	0.9% (0.9%)	65.2% (60.4%)	74.9% (72.6%)	N/A (N/A)	38.0% (33.0%)
	**Precision**	91.3% (77.1%)	84.3% (0%)	100.0% (3.0%)	100.0% (100.0%)	100.0% (94.7%)	85.4% (69.6%)	88.6% (78.9%)	N/A (N/A)	97.6% (78.5%)
	**F1**	31.7% (27.4%)	67.5% (N/A)	1.6% (0.0%)	1.6% (1.6%)	1.8% (1.7%)	74.0% (64.7%)	81.2% (75.6%)	N/A (N/A)	54.7% (46.5%)
10x	**TP**	2,195 (1,934)	3,437 (0)	697 (66)	693 (666)	749 (710)	3,313 (2,859)	3,776 (3,544)	N/A (N/A)	3,126 (2,580)
	**FP**	518	1,655	12	10	14	611	501	N/A	165
	**FN**	4,410	679	3,419	3,423	3,367	803	340	N/A	990
	**Recall**	33.2% (30.5%)	83.5% (0%)	16.9% (1.9%)	16.8% (16.3%)	18.2% (17.4%)	80.5% (78.1%)	91.7% (91.2%)	N/A (N/A)	75.9% (72.3%)
	**Precision**	80.9% (71.3%)	67.5% (0%)	98.3% (9.3%)	98.6% (94.7%)	98.2% (93.1%)	84.4% (72.9%)	88.3% (82.9%)	N/A (N/A)	95.0% (78.4%)
	**F1**	47.1% (42.7%)	74.7% (N/A)	28.9% (3.1%)	28.8% (27.8%)	30.7% (29.3%)	82.4% (75.4%)	90.0% (86.9%)	N/A (N/A)	84.4% (75.2%)
20x	**TP**	2,526 (2,322)	3,580 (0)	2,554 (976)	2,536 (2,452)	2,682 (2,584)	3,498 (3,196)	4,001 (3,908)	3,747 (3,560)	3,979 (3,599)
	**FP**	1,043	1,748	103	69	116	580	411	196	581
	**FN**	4,079	536	1,562	1,580	1,434	618	115	369	137
	**Recall**	38.2% (36.3%)	87.0% (0%)	62.1% (38.5%)	61.6% (60.8%)	65.2% (64.3%)	85.0% (83.8%)	97.2% (97.1%)	91.0% (90.6%)	96.7% (96.3%)
	**Precision**	70.8% (65.1%)	67.2% (0%)	96.1% (36.7%)	97.4% (94.1%)	95.9% (92.4%)	85.8% (78.4%)	90.7% (88.6%)	95.0% (90.3%)	87.3% (78.9%)
	**F1**	49.7% (46.6%)	75.8% (N/A)	75.4% (37.6%)	75.5% (73.9%)	77.6% (75.8%)	85.4% (81.0%)	93.8% (92.7%)	93.0% (90.4%)	91.7% (86.8%)
30x	**TP**	2,618 (2,426)	3,601 (0)	3,665 (1,649)	3,658 (3,568)	3,757 (3,676)	3,609 (3,299)	4,052 (3,996)	3,807 (3,662)	4,050 (3,802)
	**FP**	1,203	1,839	197	145	217	617	405	268	639
	**FN**	3,987	515	451	458	359	507	64	309	66
	**Recall**	39.6% (37.8%)	87.5% (0%)	89.0% (78.5%)	88.9% (88.6%)	91.3% (91.1%)	87.7% (86.7%)	98.4% (98.4%)	92.5% (92.2%)	98.4% (98.3%)
	**Precision**	68.5% (63.5%)	66.2% (0%)	94.9% (42.7%)	96.2% (93.8%)	94.5% (92.5%)	85.4% (78.1%)	90.9% (89.7%)	93.4% (89.9%)	86.4% (81.1%)
	**F1**	50.2% (47.4%)	75.4% (N/A)	91.9% (55.3%)	92.4% (91.1%)	92.9% (91.8%)	86.5% (82.1%)	94.5% (93.8%)	93.0% (91.0%)	92.0% (88.9%)
40x	**TP**	2,673 (2,476)	3,815 (0)	3,959 (1,756)	3,989 (3,898)	4,018 (3,956)	3,611 (3,339)	4,057 (4,004)	3,789 (3,599)	4,063 (3,866)
	**FP**	1,243	3,065	276	235	254	650	378	131	670
	**FN**	3,932	301	157	127	98	505	59	327	53
	**Recall**	40.5% (38.6%)	92.7% (0%)	96.2% (91.8%)	96.9% (96.8%)	97.6% (97.6%)	87.7% (86.9%)	98.6% (98.5%)	92.1% (91.7%)	98.7% (98.6%)
	**Precision**	68.3% (63.2%)	55.5% (0%)	93.5% (41.5%)	94.4% (92.3%)	94.1% (92.6%)	84.7% (78.4%)	91.5% (90.3%)	96.7% (91.8%)	85.8% (81.7%)
	**F1**	50.8% (48.0%)	69.4% (N/A)	94.8% (57.1%)	95.7% (94.5%)	95.8% (95.0%)	86.2% (82.4%)	94.9% (94.2%)	94.3% (91.7%)	91.8% (89.4%)

	INS	NanoSV (2017)	Smartie-sv_aln (2018)	Sniffles (2018)	SVIM (2019)	cuteSV (2020)	NanoVar (2020)	Sniffles2 (2022)	MAMnet (2022)	DeBreak (2022)
		Total Benchmark Calls (>50): 5281								
5x	**TP**	2,992 (2,441)	1,477 (0)	32 (0)	27 (27)	37 (36)	2,417 (1,892)	3,825 (3,172)	N/A (N/A)	1,936 (1,528)
	**FP**	764	319	4	0	4	543	390	N/A	56
	**FN**	3,716	3,804	5,249	5,254	5,244	2,864	1,456	N/A	3,345
	**Recall**	44.6% (39.6%)	28.0% (0%)	0.6% (0%)	0.5% (0.5%)	0.7% (0.7%)	45.8% (39.8%)	72.4% (68.5%)	N/A (N/A)	36.7% (31.4%)
	**Precision**	79.7% (65.0%)	82.2% (0%)	88.9% (0%)	100.0% (100.0%)	90.2% (87.8%)	81.7% (63.9%)	90.7% (75.3%)	N/A (N/A)	97.2% (76.7%)
	**F1**	57.2% (49.2%)	41.7% (N/A)	1.2% (N/A)	1.0% (1.0%)	1.4% (1.4%)	58.7% (49.0%)	80.6% (71.7%)	N/A (N/A)	53.2% (44.5%)
10x	**TP**	4,381 (3,621)	2,763 (0)	827 (42)	665 (597)	965 (898)	2,712 (2,201)	4,658 (4,103)	N/A (N/A)	3,804 (2,958)
	**FP**	2,491	1,425	37	11	68	669	489	N/A	147
	**FN**	2,327	2,518	4,454	4,616	4,316	2,569	623	N/A	1,477
	**Recall**	65.3% (60.9%)	52.3% (0%)	15.7% (0.9%)	12.6% (11.5%)	18.3% (17.2%)	51.4% (46.1%)	88.2% (86.8%)	N/A (N/A)	72.0% (66.7%)
	**Precision**	63.8% (52.7%)	66.0% (0%)	95.7% (4.9%)	98.4% (88.3%)	93.4% (86.9%)	80.2% (65.1%)	90.5% (79.7%)	N/A (N/A)	96.3% (74.9%)
	**F1**	64.5% (56.5%)	58.4% (N/A)	26.9% (1.6%)	22.3% (20.3%)	30.6% (28.7%)	62.6% (54.0%)	89.3% (83.1%)	N/A (N/A)	82.4% (70.5%)
20x	**TP**	5,035 (4,190)	2,244 (0)	3,015 (766)	2,821 (2,292)	3,379 (3,208)	2,559 (2,143)	4,944 (4,622)	4,856 (4,430)	4,866 (4,115)
	**FP**	5,463	919	249	135	213	562	445	214	234
	**FN**	1,673	3,037	2,266	2,460	1,902	2,722	337	425	415
	**Recall**	75.1% (71.5%)	42.5% (0%)	57.1% (25.3%)	53.4% (48.2%)	64.0% (62.8%)	48.5% (44.0%)	93.6% (93.2%)	92.0% (91.2%)	92.1% (90.8%)
	**Precision**	48.0% (39.9%)	70.9% (0%)	92.4% (23.5%)	95.4% (77.5%)	94.1% (89.3%)	82.0% (68.7%)	91.7% (85.8%)	95.8% (87.4%)	95.4% (80.7%)
	**F1**	58.5% (51.2%)	53.2% (N/A)	70.6% (24.3%)	68.5% (59.5%)	76.2% (73.7%)	60.9% (53.7%)	92.7% (89.3%)	93.8% (89.3%)	93.7% (85.5%)
30x	**TP**	5,105 (4,246)	1,868 (0)	4,218 (1,378)	4,152 (3,356)	4,597 (4,463)	2,397 (2,018)	5,006 (4,737)	4,990 (4,457)	4,986 (4,403)
	**FP**	6,565	717	407	384	270	493	404	223	262
	**FN**	1,603	3,413	1,063	1,129	684	2,884	275	291	295
	**Recall**	76.1% (72.6%)	35.4% (0%)	79.9% (56.5%)	78.6% (74.8%)	87.0% (86.7%)	45.4% (41.2%)	94.8% (94.5%)	94.5% (93.9%)	94.4% (93.7%)
	**Precision**	43.7% (36.4%)	72.3% (0%)	91.2% (29.8%)	91.5% (74.0%)	94.5% (91.7%)	82.9% (69.8%)	92.5% (87.6%)	95.7% (85.5%)	95.0% (83.9%)
	**F1**	55.6% (48.5%)	47.5% (N/A)	85.2% (39.0%)	84.6% (74.4%)	90.6% (89.1%)	58.7% (51.8%)	93.6% (90.9%)	95.1% (89.5%)	94.7% (88.5%)
40x	**TP**	5,100 (4,287)	2,225 (0)	4,616 (1,503)	4,650 (3,770)	4,958 (4,849)	2,265 (1,966)	5,017 (4,770)	4,919 (4,392)	5,025 (4,531)
	**FP**	6,738	1,293	553	665	277	444	401	182	266
	**FN**	1,608	3,056	665	631	323	3,016	264	362	256
	**Recall**	76.0% (72.7%)	42.1% (0%)	87.4% (69.3%)	88.1% (85.7%)	93.9% (93.8%)	42.9% (39.5%)	95.0% (94.8%)	93.1% (92.4%)	95.2% (94.7%)
	**Precision**	43.1% (36.2%)	63.2% (0%)	89.3% (29.1%)	87.5% (70.9%)	94.7% (92.6%)	83.6% (72.6%)	92.6% (88.0%)	96.4% (86.1%)	95.0% (85.6%)
	**F1**	55.0% (48.4%)	50.6% (N/A)	88.3% (41.0%)	87.8% (77.6%)	94.3% (93.2%)	56.7% (51.1%)	93.8% (91.3%)	94.8% (89.1%)	95.1% (89.9%)

Supplementary Table 9: Genome-wide SV ($>= 50bp$) evaluation against GIAB benchmark on **subsampled Nano_L1** datasets which are at 5x, 10x, 20x, 30x, and 40x (p=0, P=0.5, r=500, and O=0 for Truvari). The top and bottom panels are for deletion SVs and insertion SVs, respectively. The evaluation metrics are true positive (TP), false positive (FP), false negative (FN), recall, precision, and F1. For precision, recall, and F1, the highest values across all tools are highlighted in green color. The evaluated **read alignment-based** SV callers are NanoSV, Smartie-sv_aln, Sniffles, SVIM, cuteSV, NanoVar, Sniffles2, MAMnet, and DeBreak shown in the table in chronological order by publication year. Genotyping accuracy results are highlighted in blue. Source data are provided as a Source Data file.

DEL		Dipcall (2018)	Smartie-sv_asm (2018)	SVIM-asm (2020)	PAV (2021)
Total Benchmark Calls (>50): 4116					
	**TP**	725 (525)	2,428 (1,537)	2,411 (1,536)	2,402 (1,029)
	**FP**	142	552	499	585
5x	**FN**	3,391	1,688	1,705	1,714
	**Recall**	17.6% (13.4%)	59.0% (47.7%)	58.6% (47.4%)	58.4% (37.5%)
	**Precision**	83.6% (60.6%)	81.5% (51.6%)	82.9% (52.8%)	80.4% (34.4%)
	**F1**	29.1% (22.0%)	68.4% (49.5%)	68.6% (49.9%)	67.6% (35.9%)
	**TP**	2,324 (2,174)	3,761 (3,011)	3,759 (3,027)	3,598 (2,934)
	**FP**	235	466	355	379
10x	**FN**	1,792	355	357	518
	**Recall**	56.5% (54.8%)	91.4% (89.5%)	91.3% (89.5%)	87.4% (85.0%)
	**Precision**	90.8% (85.0%)	89.0% (71.2%)	91.4% (73.6%)	90.5% (73.8%)
	**F1**	69.6% (66.6%)	90.2% (79.3%)	91.3% (80.7%)	88.9% (79.0%)
	**TP**	3,854 (3,733)	4,017 (3,818)	4,025 (3,918)	4,050 (3,940)
	**FP**	249	298	184	182
20x	**FN**	262	99	91	66
	**Recall**	93.6% (93.4%)	97.6% (97.5%)	97.8% (97.7%)	98.4% (98.4%)
	**Precision**	93.9% (91.0%)	93.1% (88.5%)	95.6% (93.1%)	95.7% (93.1%)
	**F1**	93.8% (92.2%)	95.3% (92.8%)	96.7% (95.4%)	97.0% (95.7%)
	**TP**	3,923 (3,801)	4,030 (3,867)	4,035 (3,960)	4,062 (3,990)
	**FP**	250	290	191	172
30x	**FN**	193	86	81	54
	**Recall**	95.3% (95.2%)	97.9% (97.8%)	98.0% (98.0%)	98.7% (98.7%)
	**Precision**	94.0% (91.1%)	93.3% (89.5%)	95.5% (93.7%)	95.9% (94.2%)
	**F1**	94.7% (93.1%)	95.5% (93.5%)	96.7% (95.8%)	97.3% (96.4%)
	**TP**	3,930 (3,806)	4,028 (3,869)	4,032 (3,964)	4,059 (3,995)
	**FP**	253	294	191	170
40x	**FN**	186	88	84	57
	**Recall**	95.5% (95.3%)	97.9% (97.8%)	98.0% (97.9%)	98.6% (98.6%)
	**Precision**	94.0% (91.0%)	93.2% (89.5%)	95.5% (93.9%)	96.0% (94.5%)
	**F1**	94.7% (93.1%)	95.5% (93.5%)	96.7% (95.9%)	97.3% (96.5%)
	**TP**	3,917 (3,798)	4,027 (3,868)	4,030 (3,963)	4,063 (4,000)
	**FP**	250	291	190	164
50x	**FN**	199	89	86	53
	**Recall**	95.2% (95.0%)	97.8% (97.8%)	97.9% (97.9%)	98.7% (98.7%)
	**Precision**	94.0% (91.1%)	93.3% (89.6%)	95.5% (93.9%)	96.1% (94.6%)
	**F1**	94.6% (93.0%)	95.5% (93.5%)	96.7% (95.9%)	97.4% (96.6%)

INS		Dipcall (2018)	Smartie-sv_asm (2018)	SVIM-asm (2020)	PAV (2021)
Total Benchmark Calls (>50): 5281					
	**TP**	882 (698)	3,065 (1,826)	3,013 (1,825)	3,022 (1,387)
	**FP**	169	725	638	692
5x	**FN**	4,399	2,216	2,268	2,259
	**Recall**	16.7% (13.7%)	58.0% (45.2%)	57.1% (44.6%)	57.2% (38.0%)
	**Precision**	83.9% (66.4%)	80.9% (48.2%)	82.5% (50.0%)	81.4% (37.3%)
	**F1**	27.9% (22.7%)	67.6% (46.6%)	67.5% (47.1%)	67.2% (37.7%)
	**TP**	2,738 (2,518)	4,716 (3,620)	4,663 (3,600)	4,529 (3,578)
	**FP**	418	879	862	667
10x	**FN**	2,543	565	618	752
	**Recall**	51.8% (49.8%)	89.3% (86.5%)	88.3% (85.3%)	85.8% (82.6%)
	**Precision**	86.8% (79.8%)	84.3% (64.7%)	84.4% (65.2%)	87.2% (68.9%)
	**F1**	64.9% (61.3%)	86.7% (74.0%)	86.3% (73.9%)	86.5% (75.1%)
	**TP**	4,769 (4,496)	5,103 (4,731)	5,064 (4,855)	5,116 (4,944)
	**FP**	650	831	593	529
20x	**FN**	512	178	217	165
	**Recall**	90.3% (89.8%)	96.6% (96.4%)	95.9% (95.7%)	96.9% (96.8%)
	**Precision**	88.0% (83.0%)	86.0% (79.7%)	89.5% (85.8%)	90.6% (87.6%)
	**F1**	89.1% (86.2%)	91.0% (87.3%)	92.6% (90.5%)	93.6% (91.9%)
	**TP**	4,905 (4,633)	5,118 (4,841)	5,076 (4,957)	5,131 (5,052)
	**FP**	666	827	605	511
30x	**FN**	376	163	205	150
	**Recall**	92.9% (92.5%)	96.9% (96.7%)	96.1% (96.0%)	97.2% (97.1%)
	**Precision**	88.0% (83.2%)	86.1% (81.4%)	89.4% (87.3%)	90.9% (89.5%)
	**F1**	90.4% (87.6%)	91.2% (88.4%)	92.6% (91.4%)	93.9% (93.2%)
	**TP**	4,915 (4,632)	5,114 (4,834)	5,078 (4,956)	5,130 (5,057)
	**FP**	673	818	602	505
40x	**FN**	366	167	203	151
	**Recall**	93.1% (92.7%)	96.8% (96.7%)	96.2% (96.1%)	97.1% (97.1%)
	**Precision**	88.0% (82.9%)	86.2% (81.5%)	89.4% (87.3%)	91.0% (89.7%)
	**F1**	90.4% (87.5%)	91.2% (88.4%)	92.7% (91.4%)	94.0% (93.3%)
	**TP**	4,909 (4,632)	5,112 (4,830)	5,073 (4,943)	5,129 (5,058)
	**FP**	664	823	601	508
50x	**FN**	372	169	208	152
	**Recall**	93.0% (92.6%)	96.8% (96.6%)	96.1% (96.0%)	97.1% (97.1%)
	**Precision**	88.1% (83.1%)	86.1% (81.4%)	89.4% (87.1%)	91.0% (89.7%)
	**F1**	90.5% (87.6%)	91.2% (88.3%)	92.6% (91.3%)	94.0% (93.3%)

Supplementary Table 10: Genome-wide SV ($>= 50bp$) evaluation against GIAB benchmark on **subsampled Hifi_L1** datasets which are at 5x, 10x, 20x, 30x, 40x and 50x (p=0, P=0.5, r=500, and O=0 for Truvari). The left and right panels are for deletion SVs and insertion SVs, respectively. The evaluation metrics are true positive (TP), false positive (FP), false negative (FN), recall, precision, and F1. For precision, recall, and F1, the highest values across all tools are highlighted in green color. The evaluated **assembly-based** SV callers are Dipcall, Smartie-sv_asm, SVIM-asm, and PAV shown in the table in chronological order by publication year. Genotyping accuracy results are highlighted in blue. The input assembly file for all assembly-based SV callers is from hifiasm. Source data are provided as a Source Data file.

DEL		Dipcall (2018)	SVIM-asm (2020)	PAV (2021)
Total Benchmark Calls (>50): 4116				
5x	**TP**	65 (37)	1,549 (973)	2,643 (1,623)
	**FP**	9	103	887
	**FN**	4,051	2,567	1,473
	**Recall**	1.6% (0.9%)	37.6% (27.5%)	64.2% (52.4%)
	**Precision**	87.8% (50.0%)	93.8% (58.9%)	74.9% (46.0%)
	**F1**	3.1% (1.8%)	53.7% (37.5%)	69.1% (49.0%)
10x	**TP**	346 (216)	2,807 (1,922)	N/A (N/A)
	**FP**	62	158	N/A
	**FN**	3,770	1,309	N/A
	**Recall**	8.4% (5.4%)	68.2% (59.5%)	N/A (N/A)
	**Precision**	84.8% (52.9%)	94.7% (64.8%)	N/A (N/A)
	**F1**	15.3% (9.8%)	79.3% (62.0%)	N/A (N/A)
20x	**TP**	1,902 (1,577)	3,688 (3,299)	3,915 (3,595)
	**FP**	345	200	220
	**FN**	2,214	428	201
	**Recall**	46.2% (41.6%)	89.6% (88.5%)	95.1% (94.7%)
	**Precision**	84.6% (70.2%)	94.9% (84.9%)	94.7% (86.9%)
	**F1**	59.8% (52.2%)	92.2% (86.6%)	94.9% (90.7%)
30x	**TP**	3,179 (2,799)	3,908 (3,755)	4,025 (3,912)
	**FP**	493	201	208
	**FN**	937	208	91
	**Recall**	77.2% (74.9%)	94.9% (94.8%)	97.8% (97.7%)
	**Precision**	86.6% (76.2%)	95.1% (91.4%)	95.1% (92.4%)
	**F1**	81.6% (75.6%)	95.0% (93.0%)	96.4% (95.0%)
40x	**TP**	3,574 (3,190)	3,974 (3,882)	4,057 (3,978)
	**FP**	500	225	195
	**FN**	542	142	59
	**Recall**	86.8% (85.5%)	96.6% (96.5%)	98.6% (98.5%)
	**Precision**	87.7% (78.3%)	94.6% (92.5%)	95.4% (93.6%)
	**F1**	87.3% (81.7%)	95.6% (94.4%)	97.0% (96.0%)

INS		Dipcall (2018)	SVIM-asm (2020)	PAV (2021)
Total Benchmark Calls (>50): 5281				
5x	**TP**	57 (36)	1,889 (1,160)	3,618 (2,487)
	**FP**	15	286	1,331
	**FN**	5,224	3,392	1,663
	**Recall**	1.1% (0.7%)	35.8% (25.5%)	68.5% (59.9%)
	**Precision**	79.2% (50.0%)	86.9% (53.3%)	73.1% (50.3%)
	**F1**	2.1% (1.4%)	50.7% (34.5%)	70.7% (54.7%)
10x	**TP**	365 (258)	3,296 (2,212)	N/A (N/A)
	**FP**	95	483	N/A
	**FN**	4,916	1,985	N/A
	**Recall**	6.9% (5.0%)	62.4% (52.7%)	N/A (N/A)
	**Precision**	79.3% (56.1%)	87.2% (58.5%)	N/A (N/A)
	**F1**	12.7% (9.2%)	72.8% (55.5%)	N/A (N/A)
20x	**TP**	2,267 (1,736)	4,429 (3,817)	4,842 (4,644)
	**FP**	552	640	578
	**FN**	3,014	852	439
	**Recall**	42.9% (36.5%)	83.9% (81.8%)	91.7% (91.4%)
	**Precision**	80.4% (61.6%)	87.4% (75.3%)	89.3% (85.7%)
	**F1**	56.0% (45.9%)	85.6% (78.4%)	90.5% (88.4%)
30x	**TP**	3,907 (3,113)	4,799 (4,500)	5,034 (4,942)
	**FP**	934	638	549
	**FN**	1,374	482	247
	**Recall**	74.0% (69.4%)	90.9% (90.3%)	95.3% (95.2%)
	**Precision**	80.7% (64.3%)	88.3% (82.8%)	90.2% (88.5%)
	**F1**	77.2% (66.7%)	89.6% (86.4%)	92.7% (91.8%)
40x	**TP**	4,394 (3,609)	4,891 (4,686)	5,083 (5,014)
	**FP**	1,008	611	558
	**FN**	887	390	198
	**Recall**	83.2% (80.3%)	92.6% (92.3%)	96.3% (96.2%)
	**Precision**	81.3% (66.8%)	88.9% (85.2%)	90.1% (88.9%)
	**F1**	82.3% (72.9%)	90.7% (88.6%)	93.1% (92.4%)

Supplementary Table 11: Genome-wide SV ($>= 50bp$) evaluation against GIAB benchmark on **subsampled Nano_L1** datasets which are at 5x, 10x, 20x, 30x and 40x (p=0, P=0.5, r=500, and O=0 for Truvari). The left and right panels are for deletion SVs and insertion SVs, respectively. The evaluation metrics are true positive (TP), false positive (FP), false negative (FN), recall, precision, and F1. For precision, recall, and F1, the highest values across all tools are highlighted in green color. The evaluated **assembly-based** SV callers are Dipcall, SVIM-asm, and PAV shown in the table in chronological order by publication year. Genotyping accuracy results are highlighted in blue. The input assembly file for all assembly-based SV callers is from Flye and HapDup. Source data are provided as a Source Data file.

Hifi_L1

DEL		SVision (2022)	INSnet (2023)	INS		SVision (2022)	INSnet (2023)
Total Benchmark Calls (>50): 4116				Total Benchmark Calls (>50): 5281			
50~1k	TP	3,525 (3,466)	N/A (N/A)	50~1k	TP	4,339 (4,061)	4,309 (0)
	FP	110	N/A		FP	401	207
	FN	80	N/A		FN	222	252
	Recall	97.8% (97.7%)	N/A (N/A)		Recall	95.1% (94.8%)	94.5% (0%)
	Precision	97.0% (95.4%)	N/A (N/A)		Precision	91.5% (85.7%)	95.4% (0%)
	F1	97.4% (96.5%)	N/A (N/A)		F1	93.3% (90.0%)	94.9% (N/A)
1k~10k	TP	479 (469)	N/A (N/A)	1k~10k	TP	402 (354)	583 (0)
	FP	14	N/A		FP	15	25
	FN	2	N/A		FN	294	113
	Recall	99.6% (99.6%)	N/A (N/A)		Recall	57.8% (54.6%)	83.8% (0%)
	Precision	97.2% (95.1%)	N/A (N/A)		Precision	96.4% (84.9%)	95.9% (0%)
	F1	98.4% (97.3%)	N/A (N/A)		F1	72.2% (66.5%)	89.4% (N/A)
>10k	TP	29 (26)	N/A (N/A)	>10k	TP	2 (0)	2 (0)
	FP	4	N/A		FP	0	0
	FN	0	N/A		FN	22	22
	Recall	100.0% (100.0%)	N/A (N/A)		Recall	8.3% (0%)	8.3% (0%)
	Precision	87.9% (78.8%)	N/A (N/A)		Precision	100.0% (0%)	100.0% (0%)
	F1	93.5% (88.1%)	N/A (N/A)		F1	15.4% (N/A)	15.4% (N/A)
>50	TP	4,034 (3,962)	N/A (N/A)	>50	TP	4,747 (4,414)	4,900 (0)
	FP	126	N/A		FP	408	224
	FN	82	N/A		FN	534	381
	Recall	98.0% (98.0%)	N/A (N/A)		Recall	89.9% (89.2%)	92.8% (0%)
	Precision	97.0% (95.2%)	N/A (N/A)		Precision	92.1% (85.6%)	95.6% (0%)
	F1	97.5% (96.6%)	N/A (N/A)		F1	91.0% (87.4%)	94.2% (N/A)

Nano_L1

DEL		SVision (2022)	INSnet (2023)	INS		SVision (2022)	INSnet (2023)
Total Benchmark Calls (>50): 4116				Total Benchmark Calls (>50): 5281			
50~1k	TP	3,529 (3,374)	N/A (N/A)	50~1k	TP	4,094 (3,259)	4,127 (0)
	FP	301	N/A		FP	1,717	275
	FN	76	N/A		FN	467	434
	Recall	97.9% (97.8%)	N/A (N/A)		Recall	89.8% (87.5%)	90.5% (0%)
	Precision	92.1% (88.1%)	N/A (N/A)		Precision	70.5% (56.1%)	93.8% (0%)
	F1	94.9% (92.7%)	N/A (N/A)		F1	78.9% (68.3%)	92.1% (N/A)
1k~10k	TP	477 (462)	N/A (N/A)	1k~10k	TP	574 (468)	604 (0)
	FP	16	N/A		FP	55	52
	FN	4	N/A		FN	122	92
	Recall	99.2% (99.1%)	N/A (N/A)		Recall	82.5% (79.3%)	86.8% (0%)
	Precision	96.8% (93.7%)	N/A (N/A)		Precision	91.3% (74.4%)	92.1% (0%)
	F1	97.9% (96.4%)	N/A (N/A)		F1	86.6% (76.8%)	89.3% (N/A)
>10k	TP	29 (27)	N/A (N/A)	>10k	TP	13 (8)	15 (0)
	FP	0	N/A		FP	2	9
	FN	0	N/A		FN	11	9
	Recall	100.0% (100.0%)	N/A (N/A)		Recall	54.2% (42.1%)	62.5% (0%)
	Precision	100.0% (93.1%)	N/A (N/A)		Precision	86.7% (53.3%)	62.5% (0%)
	F1	100.0% (96.4%)	N/A (N/A)		F1	66.7% (47.1%)	62.5% (N/A)
>50	TP	4,036 (3,862)	N/A (N/A)	>50	TP	4,682 (3,735)	4,747 (0)
	FP	311	N/A		FP	1,735	305
	FN	80	N/A		FN	599	534
	Recall	98.1% (98.0%)	N/A (N/A)		Recall	88.7% (86.2%)	89.9% (0%)
	Precision	92.8% (88.8%)	N/A (N/A)		Precision	73.0% (58.2%)	94.0% (0%)
	F1	95.4% (93.2%)	N/A (N/A)		F1	80.0% (69.5%)	91.9% (N/A)

Supplementary Table 12: Genome-wide SV evaluation against GIAB benchmark on **Hifi_L1** (top panel) and **Nano_L1** (bottom panel) (p=0, P=0.5, r=500, and O=0 for Truvari). SVs are categorized into different size ranges for evaluation: 50bp-1kb, 1kb-10kb, >10kb, and >50bp. The evaluation metrics are true positive (TP), false positive (FP), false negative (FN), recall, precision and F1. For precision, recall, and F1, the highest values across all tools are highlighted in green color. The evaluated **deep learning-based** SV callers are SVision and INSnet shown in the table in chronological order by publication year. Genotyping accuracy results are highlighted in blue. Source data are provided as a Source Data file.

Hifi_L1

DEL		SVision (2022)	INSnet (2023)	INS		SVision (2022)	INSnet (2023)
Total Benchmark Calls (>50): 4116				Total Benchmark Calls (>50): 5281			
5x	**TP**	1,109 (1,027)	0 (0)	5x	**TP**	1,293 (1,212)	1,025 (0)
	**FP**	13	0		**FP**	53	43
	**FN**	3,007	4,116		**FN**	3,988	4,256
	**Recall**	26.9% (25.5%)	0% (0%)		**Recall**	24.5% (23.3%)	19.4% (0%)
	**Precision**	98.8% (91.5%)	0% (0%)		**Precision**	96.1% (90.0%)	96.0% (0%)
	**F1**	42.3% (39.8%)	N/A (N/A)		**F1**	39.0% (37.0%)	32.3% (N/A)
10x	**TP**	2,806 (2,690)	0 (0)	10x	**TP**	3,378 (3,227)	2,826 (0)
	**FP**	55	0		**FP**	213	127
	**FN**	1,310	4,116		**FN**	1,903	2,455
	**Recall**	68.2% (67.2%)	0% (0%)		**Recall**	64.0% (62.9%)	53.5% (0%)
	**Precision**	98.1% (94.0%)	0% (0%)		**Precision**	94.1% (89.9%)	95.7% (0%)
	**F1**	80.4% (78.4%)	N/A (N/A)		**F1**	76.1% (74.0%)	68.6% (N/A)
20x	**TP**	3,927 (3,848)	0 (0)	20x	**TP**	4,785 (4,627)	4,402 (0)
	**FP**	138	0		**FP**	480	209
	**FN**	189	4,116		**FN**	496	879
	**Recall**	95.4% (95.3%)	0% (0%)		**Recall**	90.6% (90.3%)	83.4% (0%)
	**Precision**	96.6% (94.7%)	0% (0%)		**Precision**	90.9% (87.9%)	95.5% (0%)
	**F1**	96.0% (95.0%)	N/A (N/A)		**F1**	90.7% (89.1%)	89.0% (N/A)
30x	**TP**	4,038 (3,957)	0 (0)	30x	**TP**	5,000 (4,839)	4,856 (0)
	**FP**	174	0		**FP**	600	257
	**FN**	78	4,116		**FN**	281	425
	**Recall**	98.1% (98.1%)	0% (0%)		**Recall**	94.7% (94.5%)	92.0% (0%)
	**Precision**	95.9% (93.9%)	0% (0%)		**Precision**	89.3% (86.4%)	95.0% (0%)
	**F1**	97.0% (96.0%)	N/A (N/A)		**F1**	91.9% (90.3%)	93.4% (N/A)
40x	**TP**	3,994 (3,926)	0 (0)	40x	**TP**	4,870 (4,733)	4,965 (0)
	**FP**	134	0		**FP**	467	274
	**FN**	122	4,116		**FN**	411	316
	**Recall**	97.0% (97.0%)	0% (0%)		**Recall**	92.2% (92.0%)	94.0% (0%)
	**Precision**	96.8% (95.1%)	0% (0%)		**Precision**	91.2% (88.7%)	94.8% (0%)
	**F1**	96.9% (96.0%)	N/A (N/A)		**F1**	91.7% (90.3%)	94.4% (N/A)
50x	**TP**	4,038 (3,966)	0 (0)	50x	**TP**	4,968 (4,828)	4,998 (0)
	**FP**	158	0		**FP**	538	301
	**FN**	78	4,116		**FN**	313	283
	**Recall**	98.1% (98.1%)	0% (0%)		**Recall**	94.1% (93.9%)	94.6% (0%)
	**Precision**	96.2% (94.5%)	0% (0%)		**Precision**	90.2% (87.7%)	94.3% (0%)
	**F1**	97.2% (96.3%)	N/A (N/A)		**F1**	92.1% (90.7%)	94.5% (N/A)

Nano_L1

DEL		SVision (2022)	INSnet (2023)	INS		SVision (2022)	INSnet (2023)
Total Benchmark Calls (>50): 4116				Total Benchmark Calls (>50): 5281			
5x	**TP**	885 (810)	0 (0)	5x	**TP**	883 (749)	849 (0)
	**FP**	13	0		**FP**	129	88
	**FN**	3,231	4,116		**FN**	4,398	4,432
	**Recall**	21.5% (20.0%)	0% (0%)		**Recall**	16.7% (14.6%)	16.1% (0%)
	**Precision**	98.6% (90.2%)	0% (0%)		**Precision**	87.3% (74.0%)	90.6% (0%)
	**F1**	35.3% (32.8%)	N/A (N/A)		**F1**	28.1% (24.3%)	27.3% (N/A)
10x	**TP**	2,522 (2,322)	0 (0)	10x	**TP**	2,839 (2,249)	2,448 (0)
	**FP**	81	0		**FP**	468	172
	**FN**	1,594	4,116		**FN**	2,442	2,833
	**Recall**	61.3% (59.3%)	0% (0%)		**Recall**	53.8% (47.9%)	46.4% (0%)
	**Precision**	96.9% (89.2%)	0% (0%)		**Precision**	85.8% (68.0%)	93.4% (0%)
	**F1**	75.1% (71.2%)	N/A (N/A)		**F1**	66.1% (56.2%)	62.0% (N/A)
20x	**TP**	3,863 (3,667)	0 (0)	20x	**TP**	4,417 (3,519)	4,009 (0)
	**FP**	309	0		**FP**	1,468	213
	**FN**	253	4,116		**FN**	864	1,272
	**Recall**	93.9% (93.5%)	0% (0%)		**Recall**	83.6% (80.3%)	75.9% (0%)
	**Precision**	92.6% (87.9%)	0% (0%)		**Precision**	75.1% (59.8%)	95.0% (0%)
	**F1**	93.2% (90.6%)	N/A (N/A)		**F1**	79.1% (68.5%)	84.4% (N/A)
30x	**TP**	4,051 (3,833)	0 (0)	30x	**TP**	4,804 (3,756)	4,502 (0)
	**FP**	652	0		**FP**	2,557	259
	**FN**	65	4,116		**FN**	477	779
	**Recall**	98.4% (98.3%)	0% (0%)		**Recall**	91.0% (88.7%)	85.2% (0%)
	**Precision**	86.1% (81.5%)	0% (0%)		**Precision**	65.3% (51.0%)	94.6% (0%)
	**F1**	91.9% (89.1%)	N/A (N/A)		**F1**	76.0% (64.8%)	89.7% (N/A)
40x	**TP**	3,959 (3,791)	0 (0)	40x	**TP**	4,492 (3,599)	4,670 (0)
	**FP**	225	0		**FP**	1,259	283
	**FN**	157	4,116		**FN**	789	611
	**Recall**	96.2% (96.0%)	0% (0%)		**Recall**	85.1% (82.0%)	88.4% (0%)
	**Precision**	94.6% (90.6%)	0% (0%)		**Precision**	78.1% (62.6%)	94.3% (0%)
	**F1**	95.4% (93.2%)	N/A (N/A)		**F1**	81.4% (71.0%)	91.3% (N/A)

Supplementary Table 13: Genome-wide SV ($>= 50bp$) evaluation against GIAB benchmark on **subsampled Hifi_L1** and **subsampled Nano_L1** datasets which are at 5x, 10x, 20x, 30x, 40x and 50x (p=0, P=0.5, r=500, and O=0 for Truvari). The left and right panels are for deletion SVs and insertion SVs, respectively. The evaluation metrics are true positive (TP), false positive (FP), false negative (FN), recall, precision, and F1. For precision, recall, and F1, the highest values across all tools are highlighted in green color. The evaluated **deep learning-based** SV callers are SVision and INSnet shown in the table in chronological order by publication year. Genotyping accuracy results are highlighted in blue. Source data are provided as a Source Data file.

4 Supplementary Figures

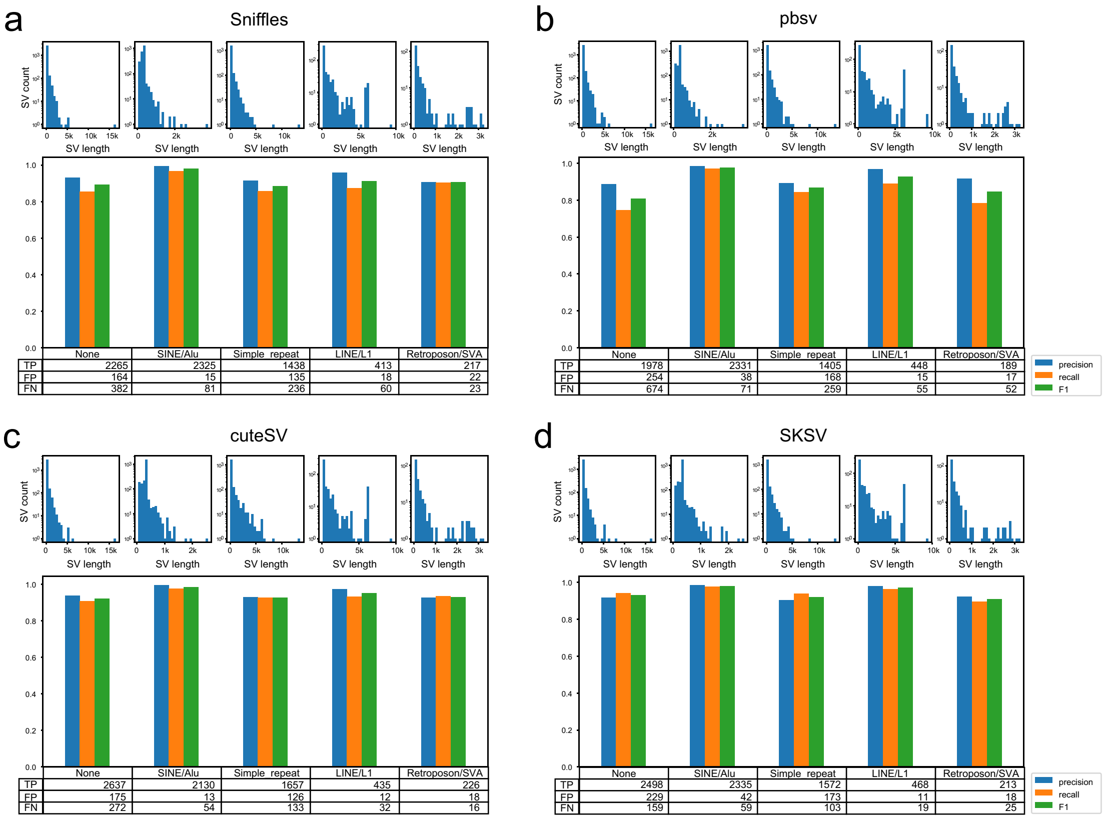

Supplementary Figure 1: RepeatMasker annotation for SV calls of Sniffles, pbsv, cuteSV and SKSV on **Hifi_L1** (**a-d**). The top panel is the SV length distribution by different repeat annotation categories. The bar plot in the middle panel is the precision, recall, and F1 score of SVs belonging to each repeat annotation category. The table in the bottom panel is the number of true positives, false positives, and false negatives in each repeat annotation category. Source data are provided as a Source Data file.

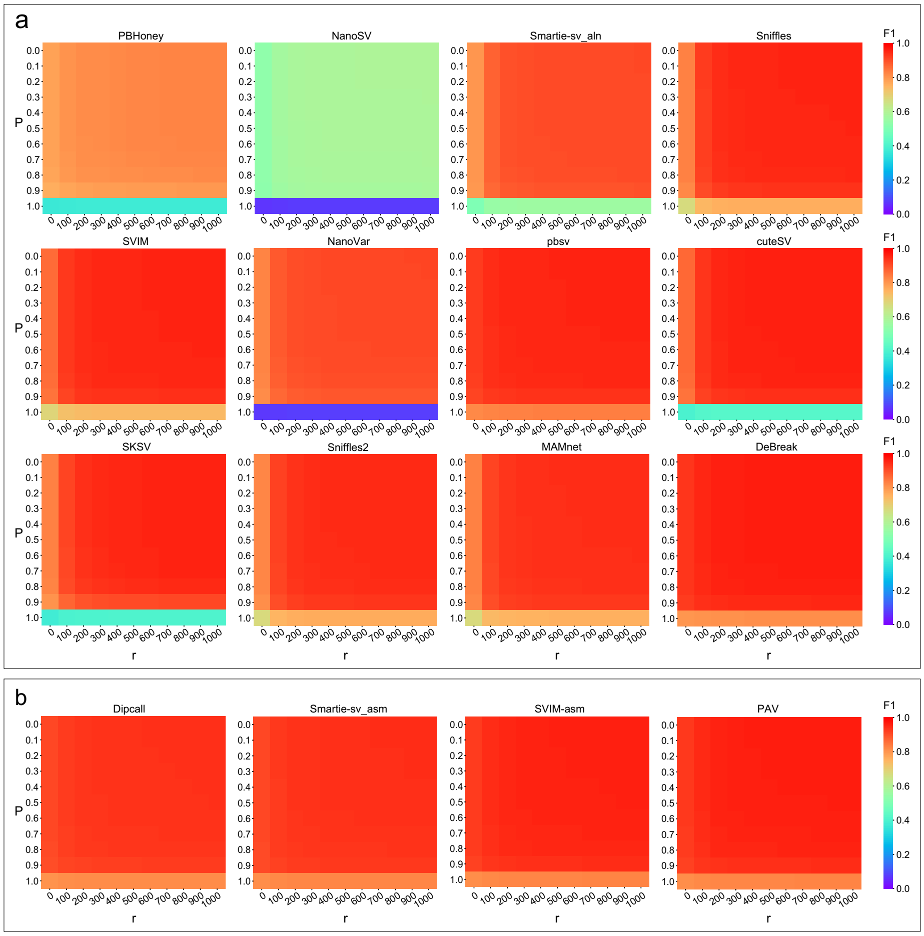

Supplementary Figure 2: F1 accuracy for detection of **deletion SVs** by tuning different evaluation parameters P and r on **Hifi_L1**. **a.** Grid search heatmap of F1 values for deletion SVs by different read alignment-based tools. **b.** Grid search F1 heatmap for deletion SVs by different assembly-based tools. P is the minimum percentage of allele size similarity between SV call and gold standard SV, which varies from 0-1 with a 0.1 interval. r is the maximum reference location distance between SV call and gold standard SV, which varies from 0-1000bp with a 100bp interval. Source data are provided as a Source Data file.

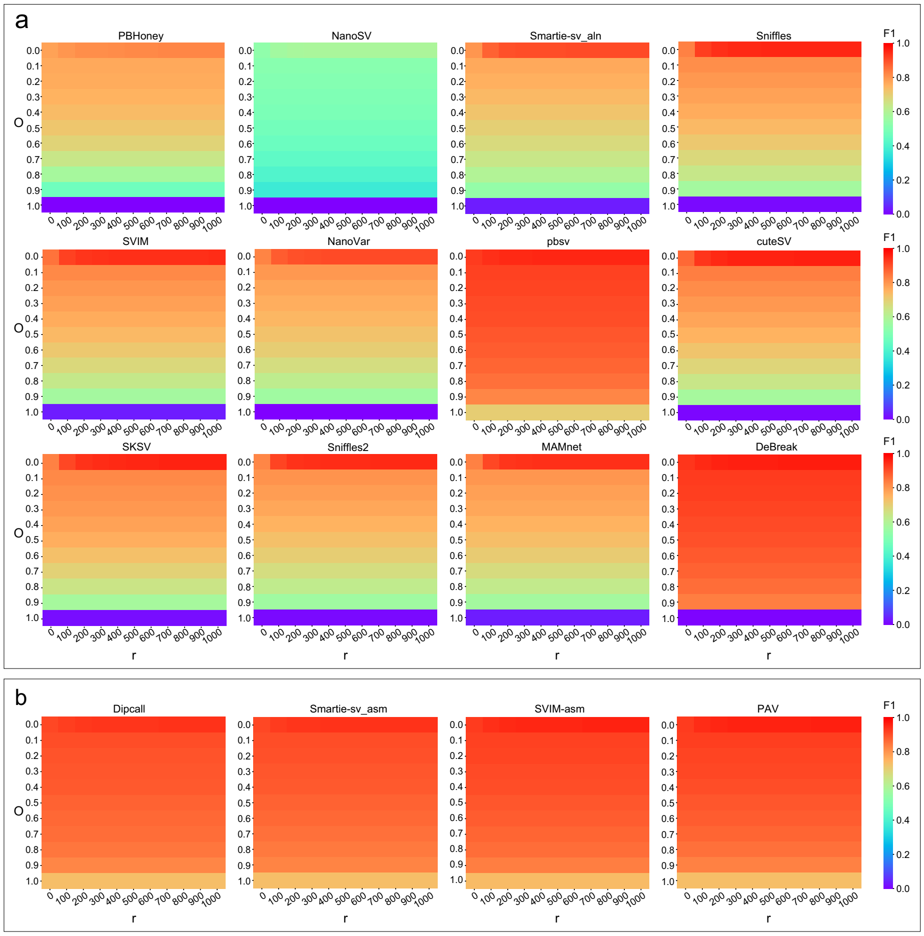

Supplementary Figure 3: F1 accuracy for detection of **deletion SVs** by tuning different evaluation parameters O and r on **Hifi_L1**. **a.** Grid search heatmap of F1 values for deletion SVs by different read alignment-based tools. **b.** Grid search F1 heatmap for deletion SVs by different assembly-based tools. O is the minimum reciprocal overlap between SV call and gold standard SV, which varies from 0-1 with a 0.1 interval. r is the maximum reference location distance between SV call and gold standard SV, which varies from 0-1000bp with a 100bp interval. Source data are provided as a Source Data file.

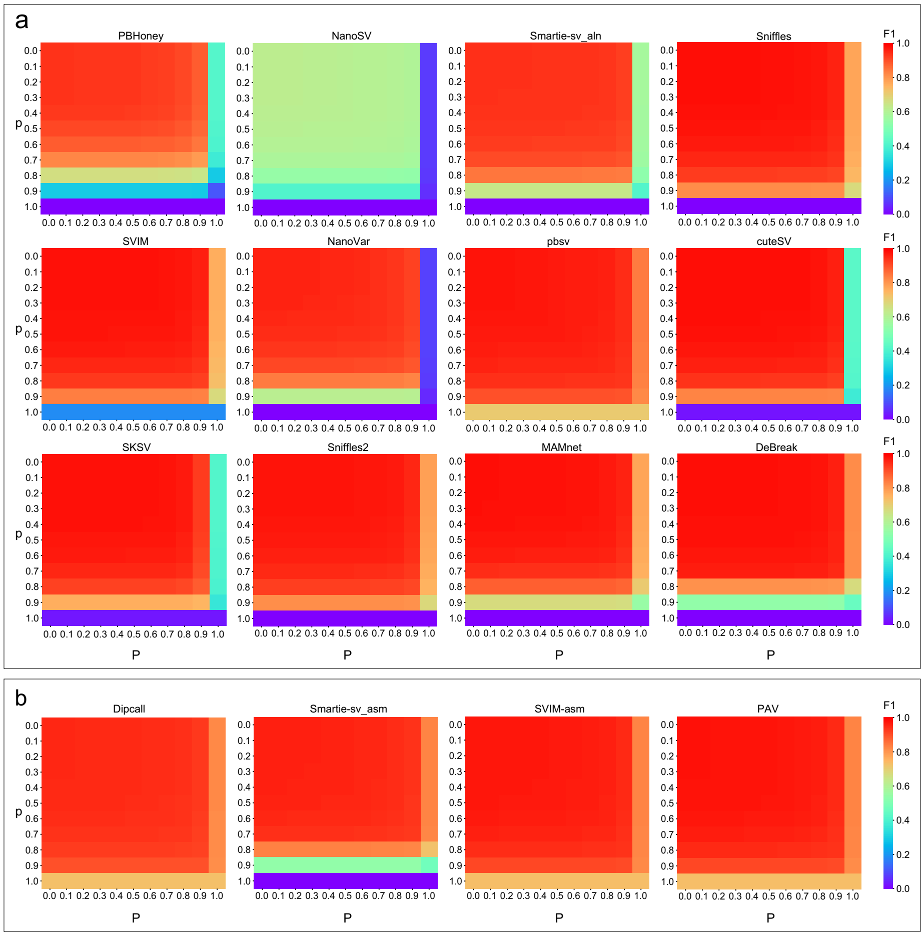

Supplementary Figure 4: F1 accuracy for detection of **deletion SVs** by tuning different evaluation parameters p and P on **Hifi_L1**. **a.** Grid search heatmap of F1 values for deletion SVs by different read alignment-based tools. **b.** Grid search F1 heatmap for deletion SVs by different assembly-based tools. p is the minimum percentage of allele sequence similarity between SV call and gold standard SV, which varies from 0-1 with a 0.1 interval. P is the minimum percentage of allele size similarity between SV call and gold standard SV, which varies from 0-1 with a 0.1 interval. Source data are provided as a Source Data file.

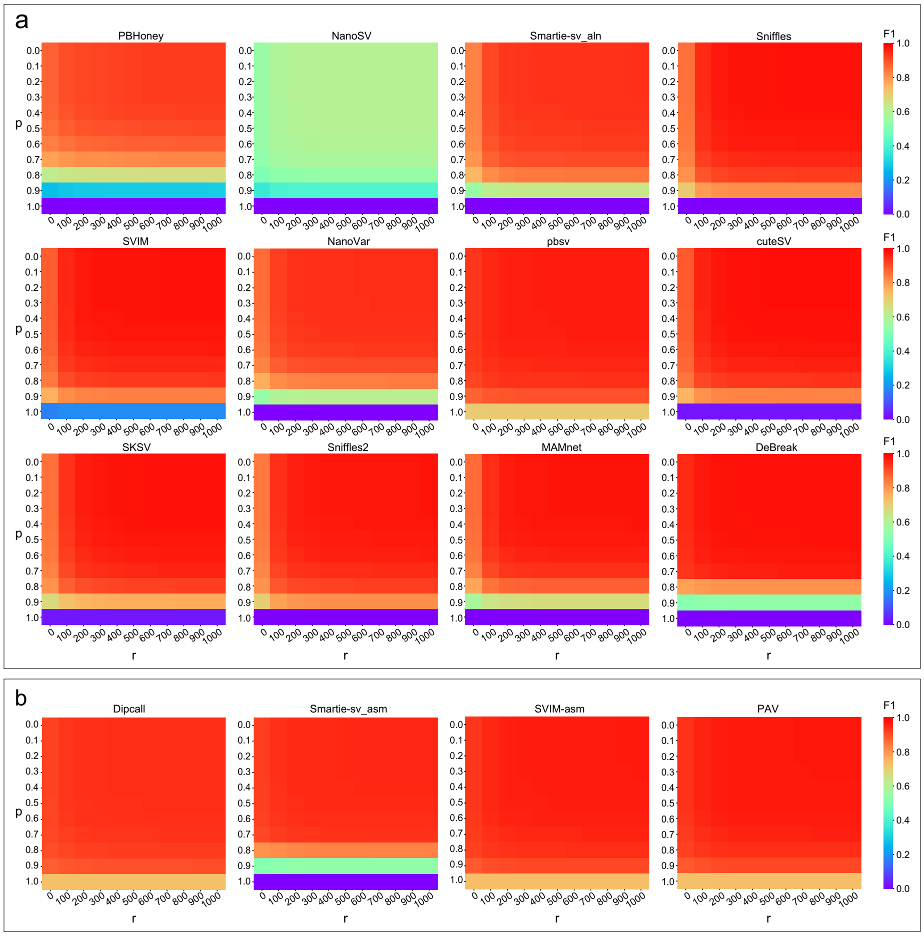

Supplementary Figure 5: F1 accuracy for detection of **deletion SVs** by tuning different evaluation parameters p and r on **Hifi_L1**. **a.** Grid search heatmap of F1 values for deletion SVs by different read alignment-based tools. **b.** Grid search F1 heatmap for deletion SVs by different assembly-based tools. p is the minimum percentage of allele sequence similarity between SV call and gold standard SV, which varies from 0-1 with a 0.1 interval. r is the maximum reference location distance between SV call and gold standard SV, which varies from 0-1000bp with a 100bp interval. Source data are provided as a Source Data file.

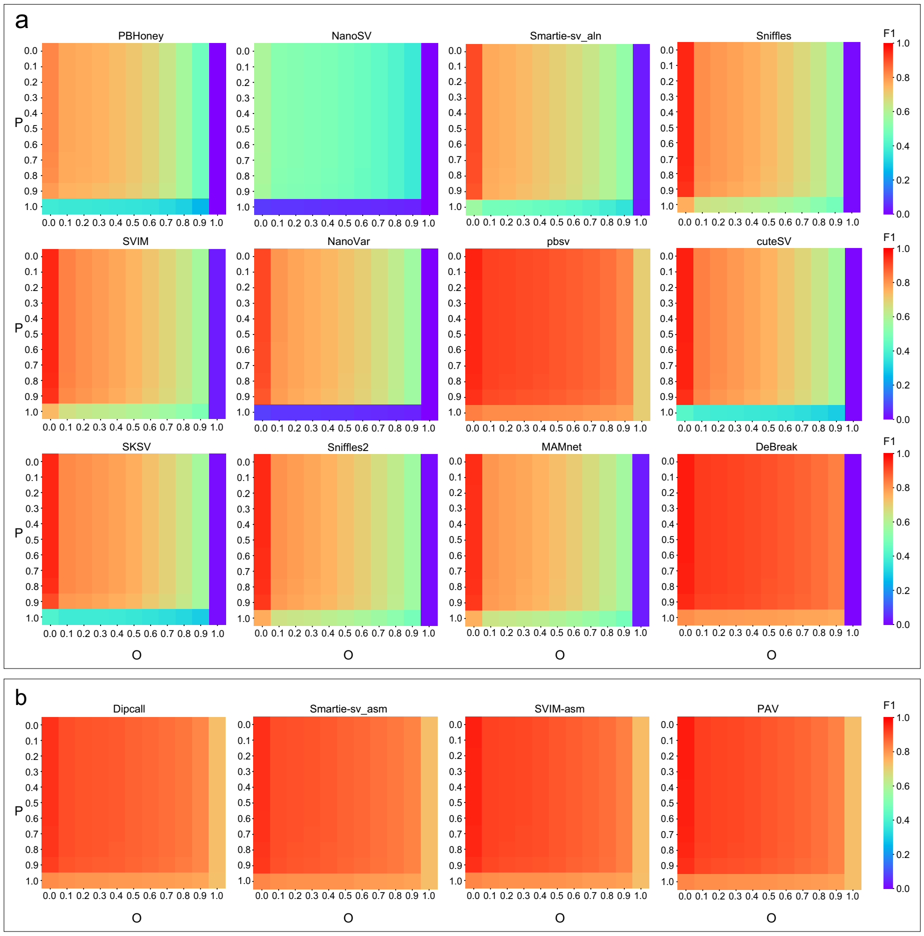

Supplementary Figure 6: F1 accuracy for detection of **deletion SVs** by tuning different evaluation parameters P and O on **Hifi_L1**. **a.** Grid search heatmap of F1 values for deletion SVs by different read alignment-based tools. **b.** Grid search F1 heatmap for deletion SVs by different assembly-based tools. P is the minimum percentage of allele size similarity between SV call and gold standard SV, which varies from 0-1 with a 0.1 interval. O is the minimum reciprocal overlap between SV call and gold standard SV, which varies from 0-1 with a 0.1 interval. Source data are provided as a Source Data file.

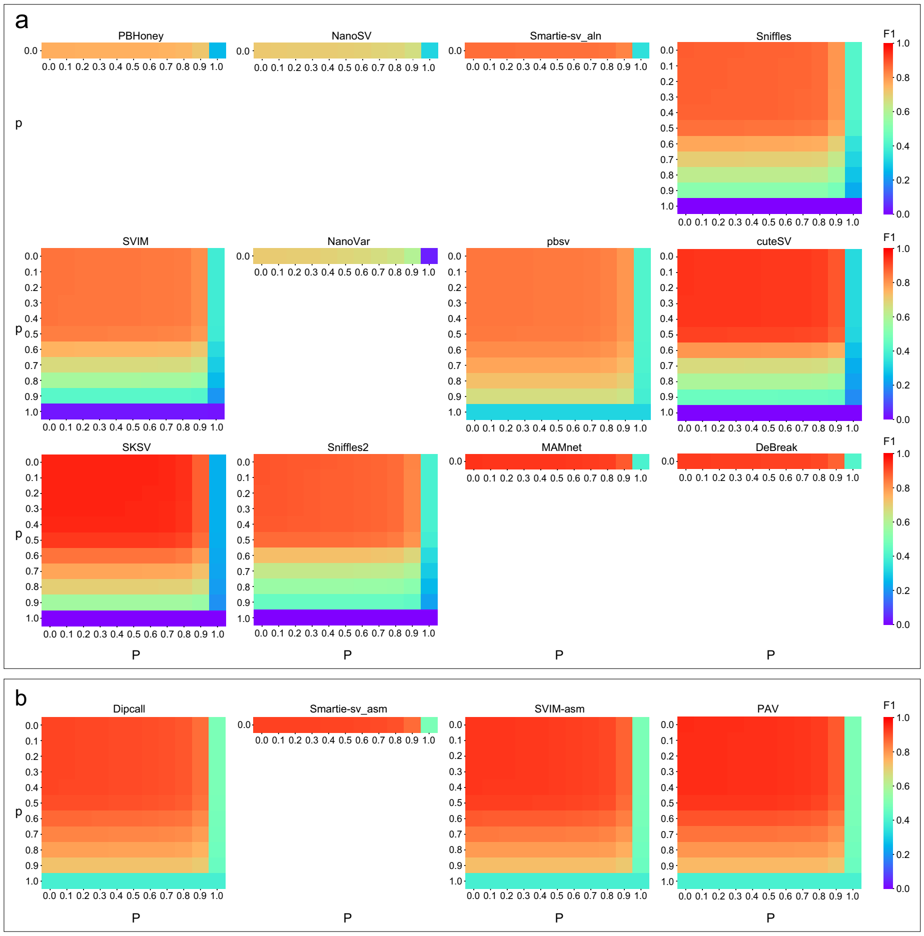

Supplementary Figure 7: F1 accuracy for detection of **insertion SVs** by tuning different evaluation parameters p and P on **Hifi_L1**. **a.** Grid search heatmap of F1 values for insertion SVs by different read alignment-based tools. **b.** Grid search F1 heatmap for insertion SVs by different assembly-based tools. p is the minimum percentage of allele sequence similarity between SV call and gold standard SV, which varies from 0-1 with 0.1 interval. P is the minimum percentage of allele size similarity between SV call and gold standard SV, which varies from 0-1 with 0.1 interval. Source data are provided as a Source Data file.

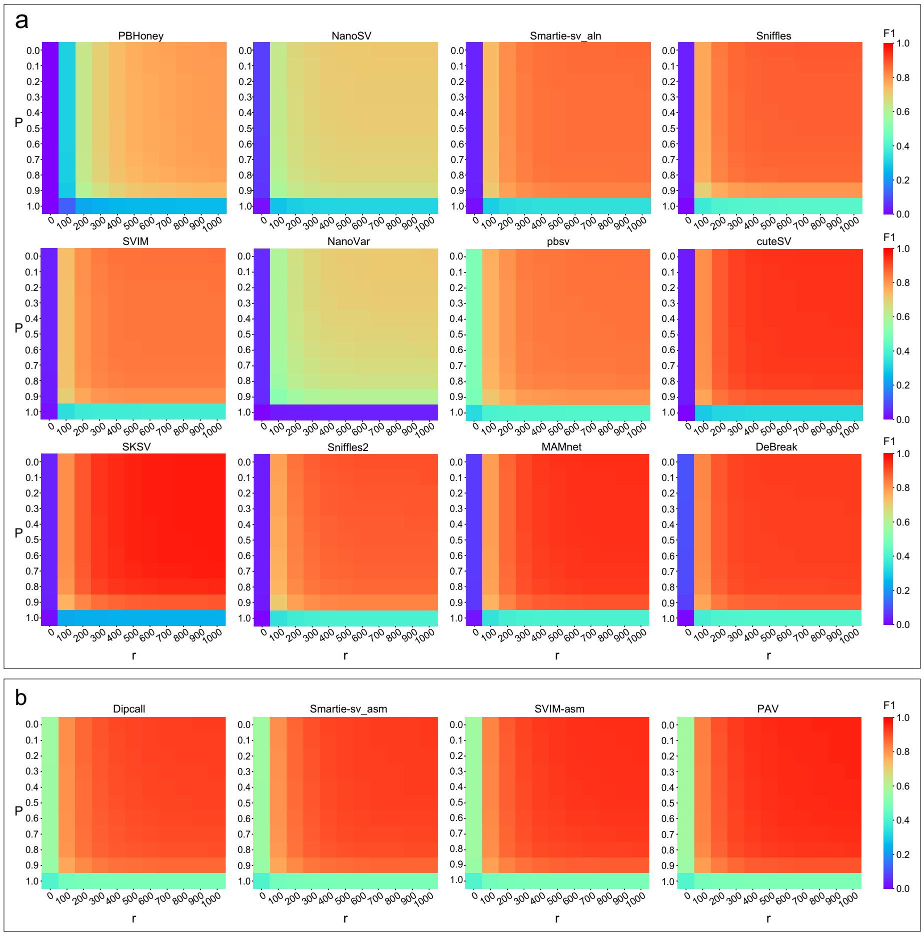

Supplementary Figure 8: F1 accuracy for detection of **insertion SVs** by tuning different evaluation parameters P and r on **Hifi_L1**. **a.** Grid search heatmap of F1 values for insertion SVs by different read alignment-based tools. **b.** Grid search F1 heatmap for insertion SVs by different assembly-based tools. P is the minimum percentage of allele size similarity between SV call and gold standard SV, which varies from 0-1 with a 0.1 interval. r is the maximum reference location distance between SV call and gold standard SV, which varies from 0-1000bp with a 100bp interval. Source data are provided as a Source Data file.

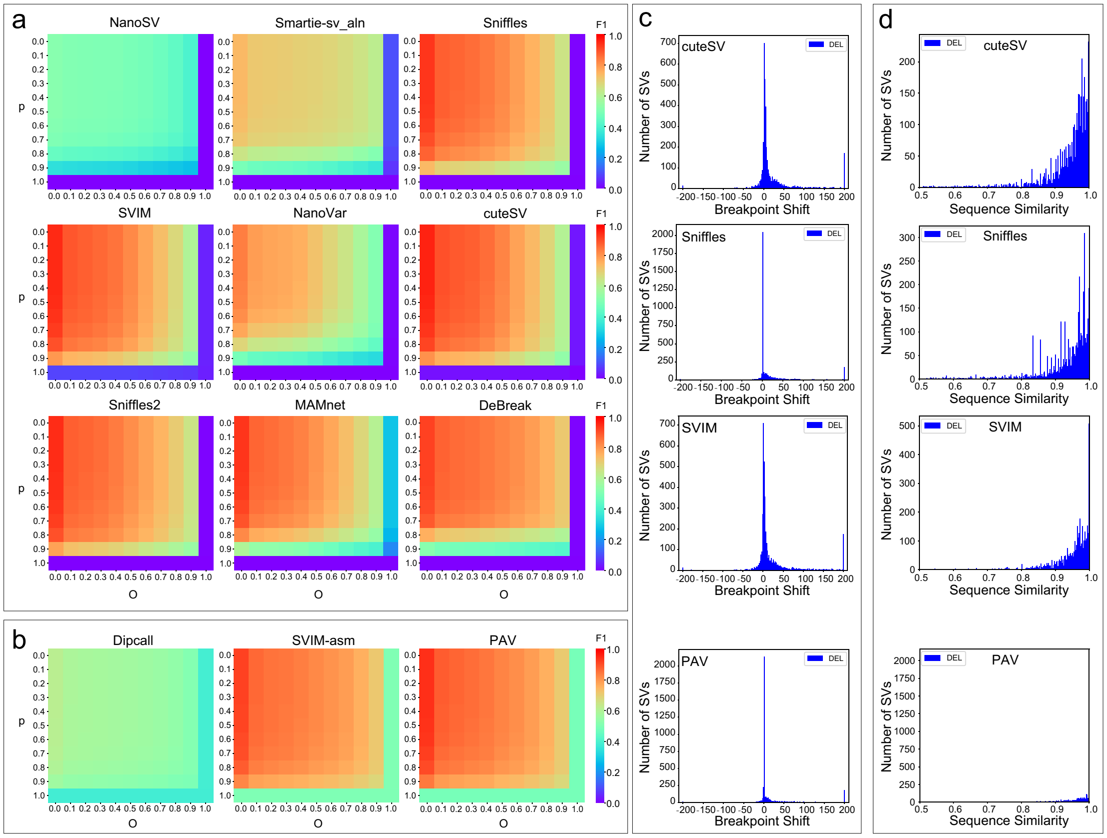

Supplementary Figure 9: F1 accuracy for detection of **deletion SVs** by tuning different evaluation parameters O and p and distribution of breakpoints shift for **deletion SVs** on **Nano_L1**. **a.** Grid search heatmap of F1 values for deletion SVs by different read alignment-based tools. **b.** Grid search F1 heatmap for deletion SVs by different assembly-based tools. O is the minimum reciprocal overlap between SV call and gold standard SV. p is the minimum percentage of allele sequence similarity between SV call and gold standard SV. O and p vary from 0-1 with a 0.1 interval. **c.** Distribution of breakpoints shift for deletion SVs by cuteSV, Sniffles, and PAV. **d.** Distribution of alternate sequence similarity for deletion SVs by cuteSV, Sniffles, and PAV. Source data are provided as a Source Data file.

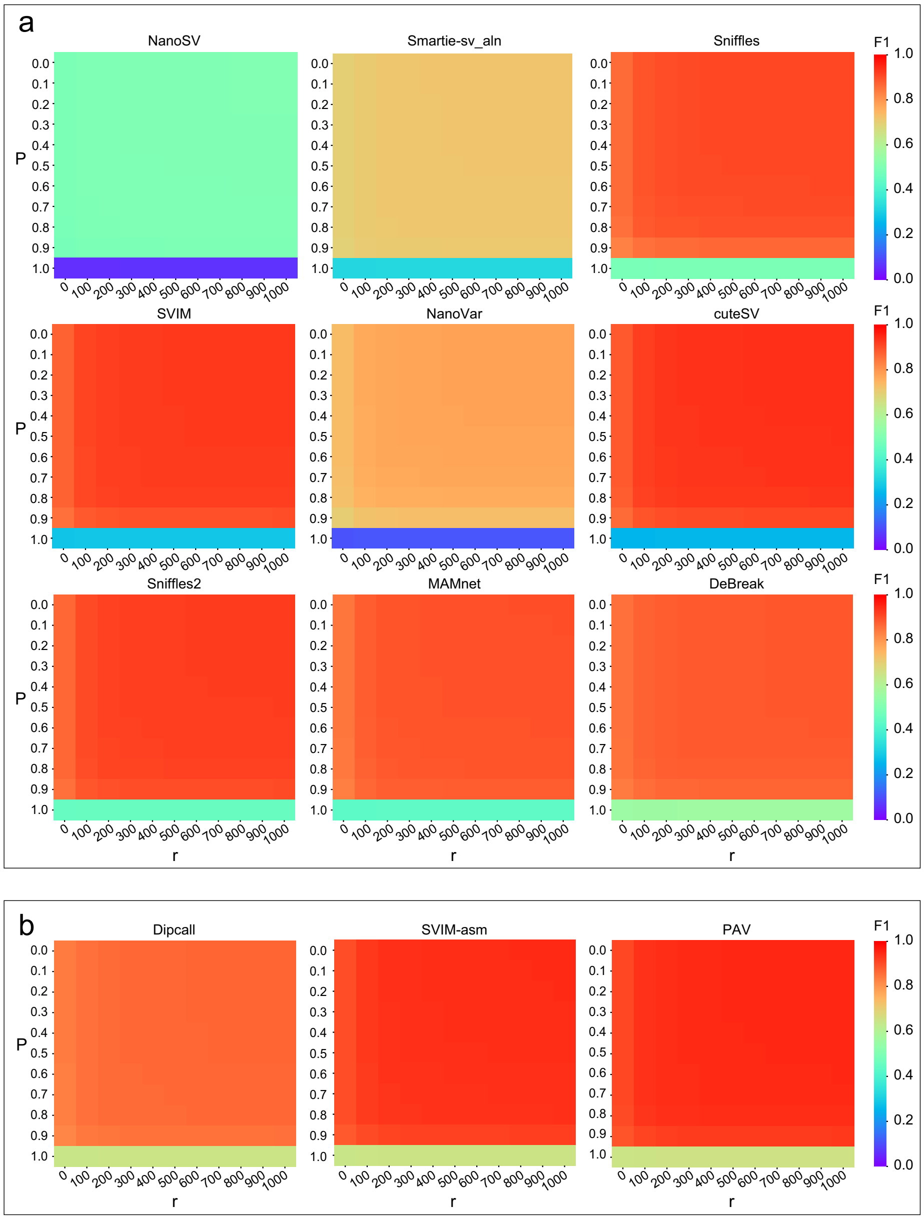

Supplementary Figure 10: F1 accuracy for detection of **deletion SVs** by tuning different evaluation parameters P and r on **Nano_L1**. **a.** Grid search heatmap of F1 values for deletion SVs by different read alignment-based tools. **b.** Grid search F1 heatmap for deletion SVs by different assembly-based tools. P is the minimum percentage of allele size similarity between SV call and gold standard SV, which varies from 0-1 with a 0.1 interval. r is the maximum reference location distance between SV call and gold standard SV, which varies from 0-1000bp with a 100bp interval. Source data are provided as a Source Data file.

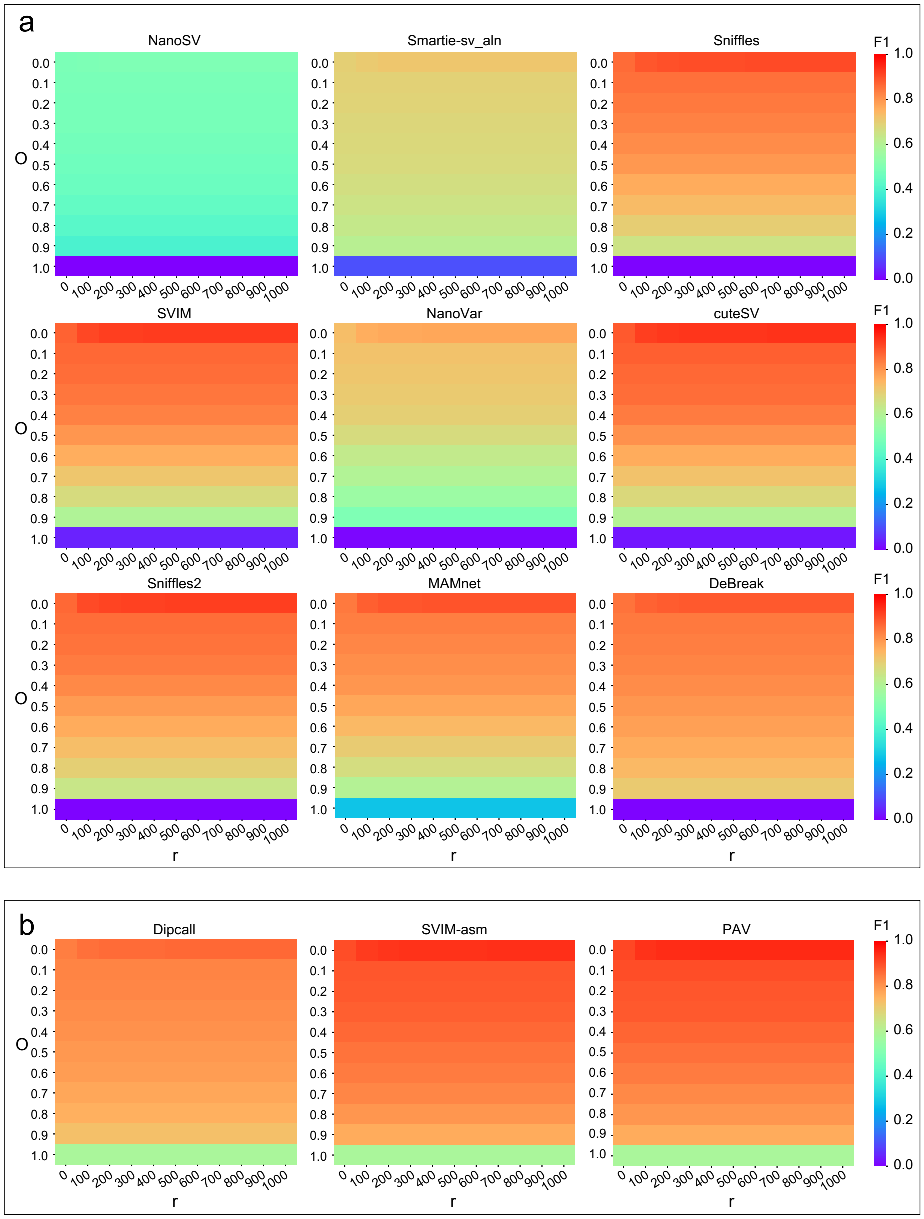

Supplementary Figure 11: F1 accuracy for detection of **deletion SVs** by tuning different evaluation parameters O and r on **Nano_L1**. **a.** Grid search heatmap of F1 values for deletion SVs by different read alignment-based tools. **b.** Grid search F1 heatmap for deletion SVs by different assembly-based tools. O is the minimum reciprocal overlap between SV call and gold standard SV, which varies from 0-1 with a 0.1 interval. r is the maximum reference location distance between SV call and gold standard SV, which varies from 0-1000bp with a 100bp interval. Source data are provided as a Source Data file.

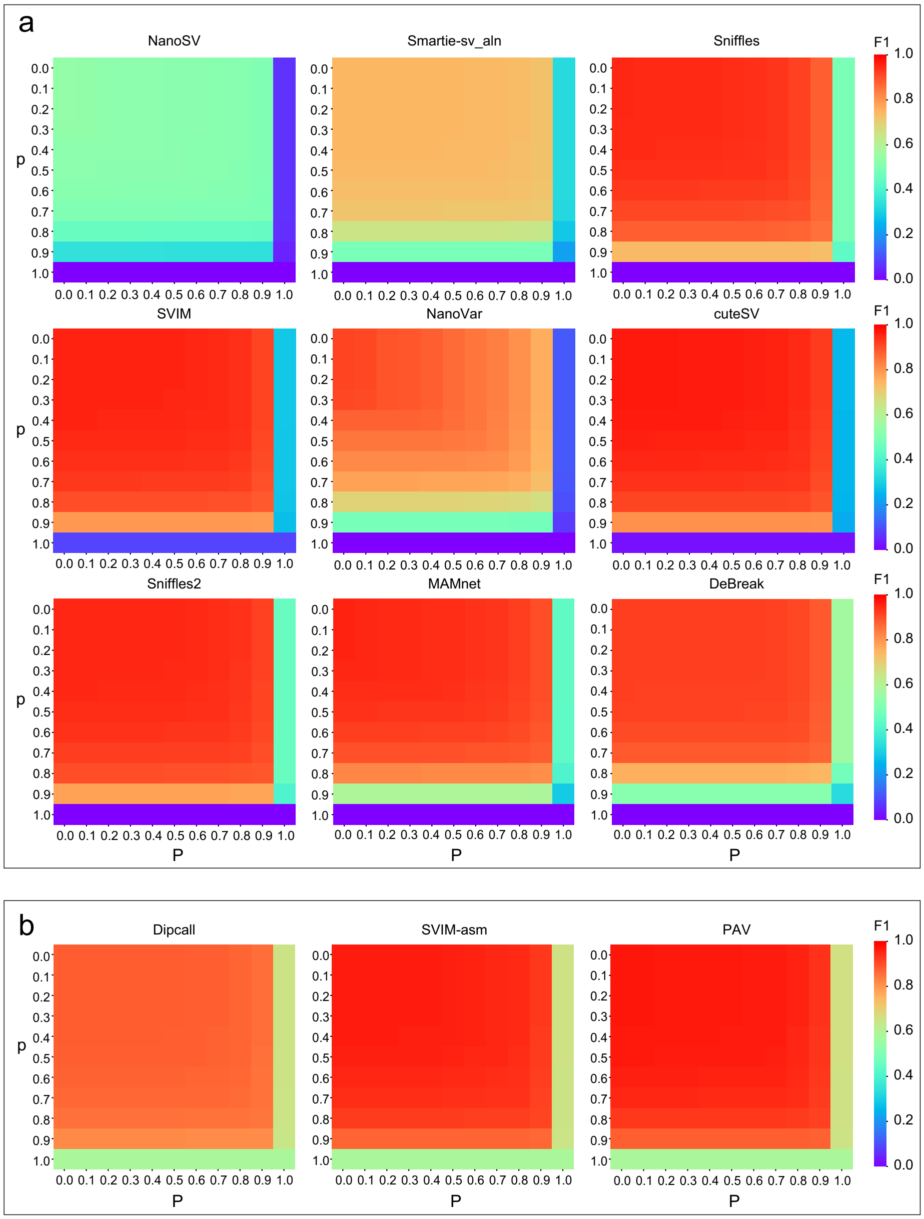

Supplementary Figure 12: F1 accuracy for detection of **deletion SVs** by tuning different evaluation parameters p and P on **Nano_L1**. **a.** Grid search heatmap of F1 values for deletion SVs by different read alignment-based tools. **b.** Grid search F1 heatmap for deletion SVs by different assembly-based tools. p is the minimum percentage of allele sequence similarity between SV call and gold standard SV, which varies from 0-1 with a 0.1 interval. P is the minimum percentage of allele size similarity between SV call and gold standard SV, which varies from 0-1 with a 0.1 interval. Source data are provided as a Source Data file.

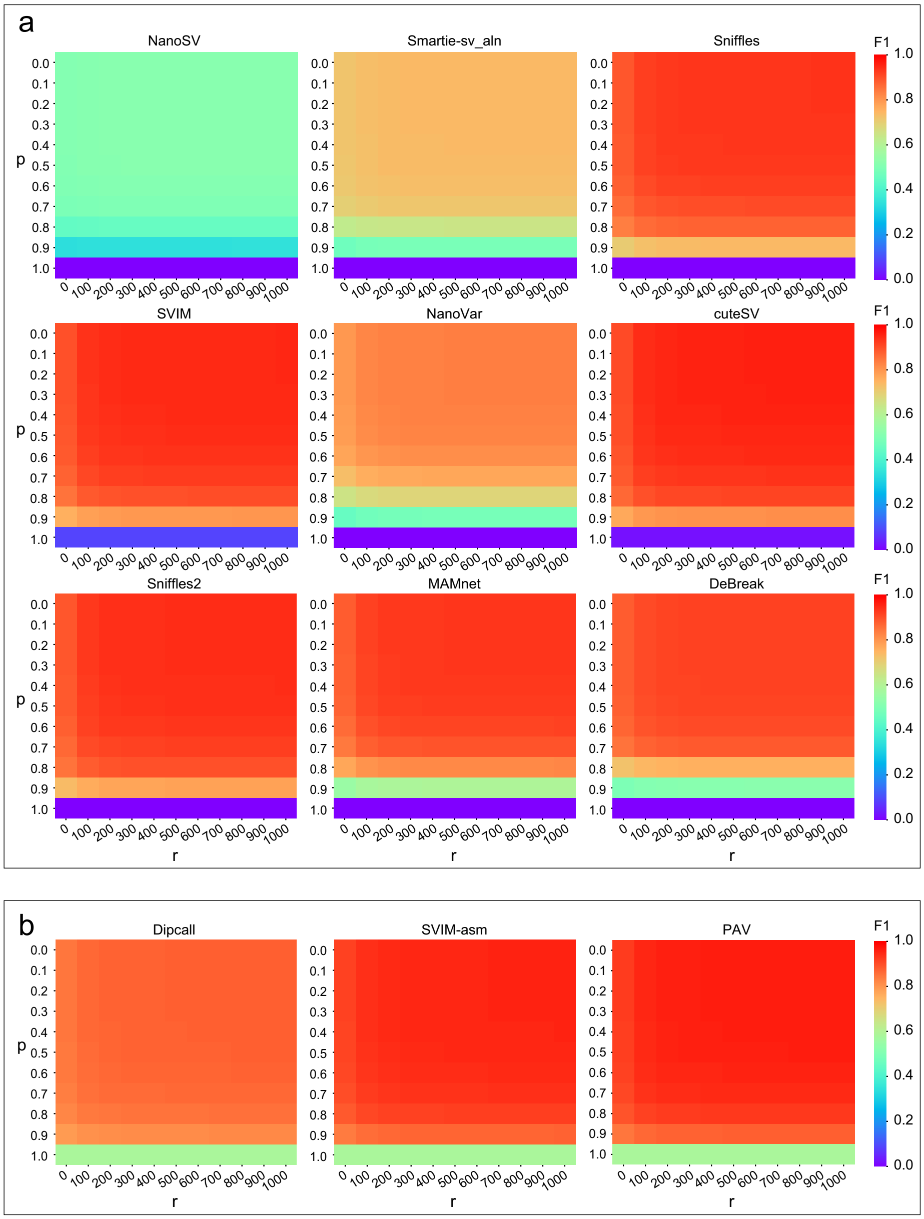

Supplementary Figure 13: F1 accuracy for detection of **deletion SVs** by tuning different evaluation parameters p and r on **Nano_L1**. **a.** Grid search heatmap of F1 values for deletion SVs by different read alignment-based tools. **b.** Grid search F1 heatmap for deletion SVs by different assembly-based tools. p is the minimum percentage of allele sequence similarity between SV call and gold standard SV, which varies from 0-1 with a 0.1 interval. r is the maximum reference location distance between SV call and gold standard SV, which varies from 0-1000bp with a 100bp interval. Source data are provided as a Source Data file.

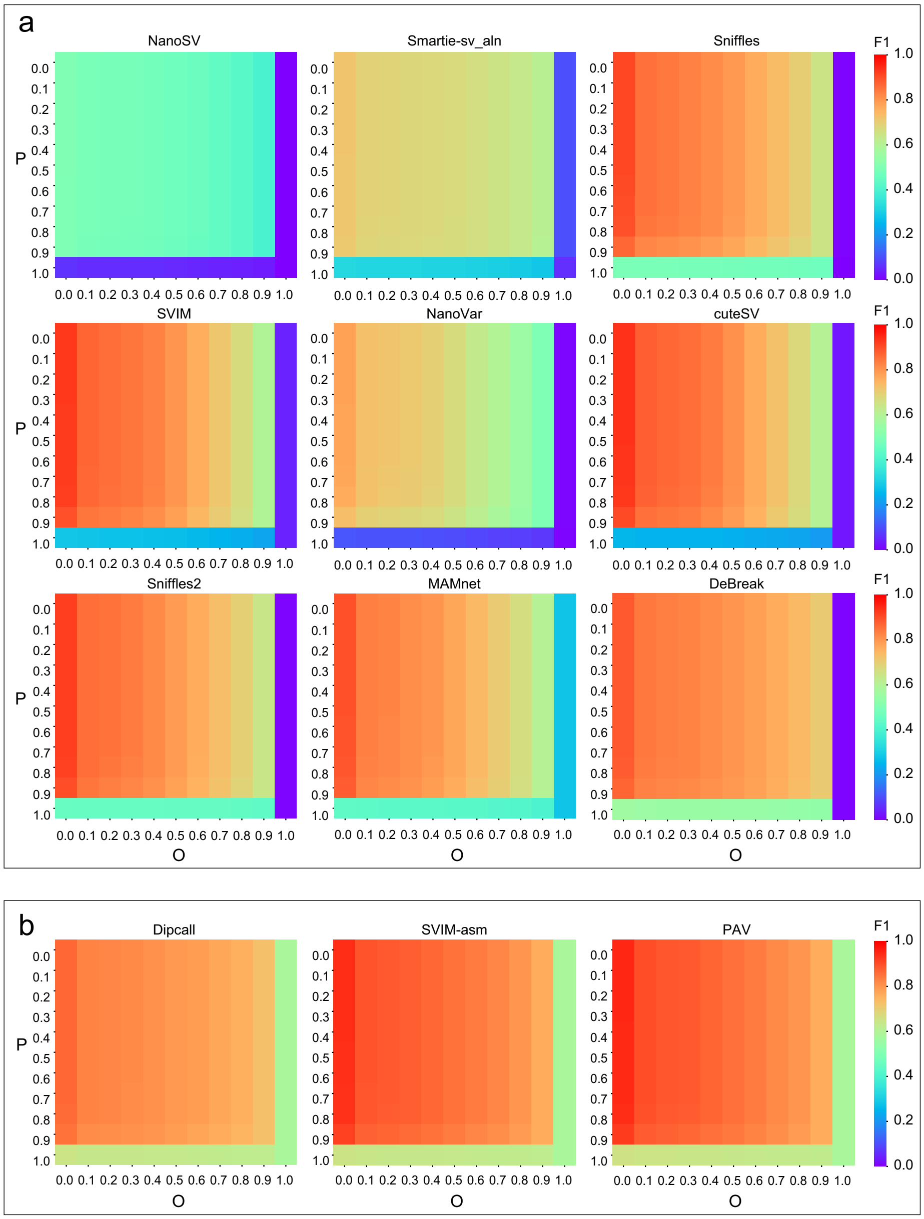

Supplementary Figure 14: F1 accuracy for detection of **deletion SVs** by tuning different evaluation parameters P and O on **Nano_L1**. **a.** Grid search heatmap of F1 values for deletion SVs by different read alignment-based tools. **b.** Grid search F1 heatmap for deletion SVs by different assembly-based tools. P is the minimum percentage of allele size similarity between SV call and gold standard SV, which varies from 0-1 with a 0.1 interval. O is the minimum reciprocal overlap between SV call and gold standard SV, which varies from 0-1 with a 0.1 interval. Source data are provided as a Source Data file.

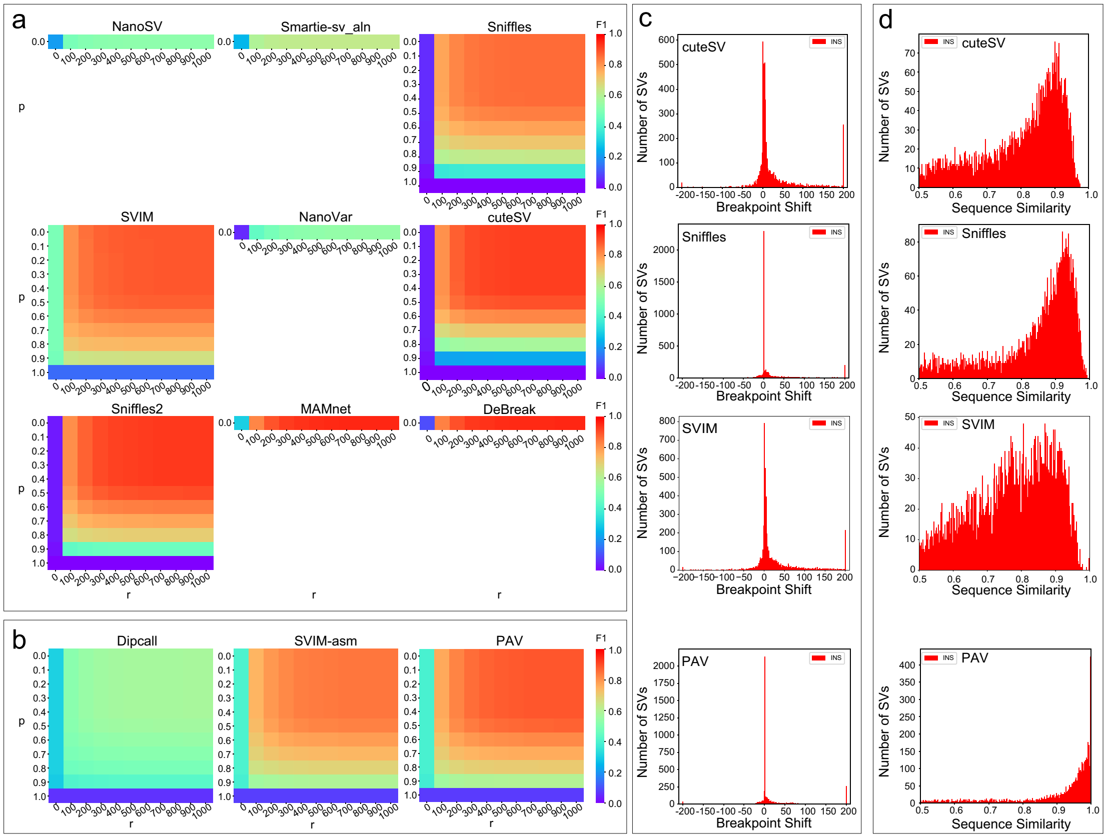

Supplementary Figure 15: F1 accuracy for detection of **insertion SVs** by tuning different evaluation parameters p and r and distribution of breakpoints shift for **insertion SVs** on **Nano_L1**. **a.** Grid search heatmap of F1 values for insertion SVs by different read alignment-based tools. **b.** Grid search F1 heatmap for insertion SVs by different assembly-based tools. p is the minimum percentage of allele sequence similarity between SV call and gold standard SV, which varies from 0-1 with a 0.1 interval. r is the maximum reference location distance between SV call and gold standard SV, which varies from 0-1000bp with a 100bp interval. **c.** Distribution of breakpoints shift for deletion SVs by cuteSV, Sniffles and PAV. **d.** Distribution of alternate sequence similarity for deletion SVs by cuteSV, Sniffles and PAV. Source data are provided as a Source Data file.

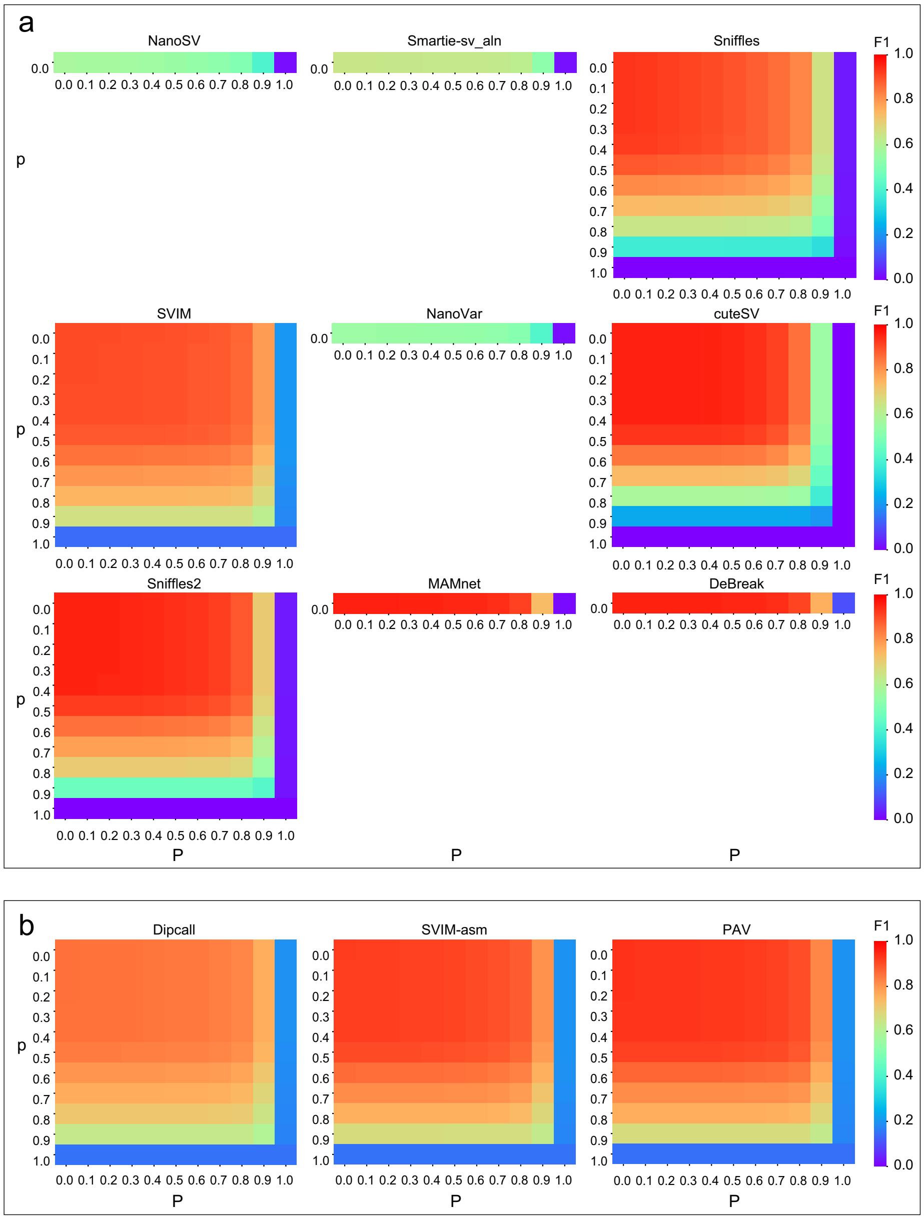

Supplementary Figure 16: F1 accuracy for detection of **insertion SVs** by tuning different evaluation parameters P and p on **Nano_L1**. **a.** Grid search heatmap of F1 values for insertion SVs by different read alignment-based tools. **b.** Grid search F1 heatmap for insertion SVs by different assembly-based tools. P is the minimum percentage of allele size similarity between SV call and gold standard SV, which varies from 0-1 with a 0.1 interval. p is the minimum percentage of allele sequence similarity between SV call and gold standard SV, which varies from 0-1 with a 0.1 interval. Source data are provided as a Source Data file.

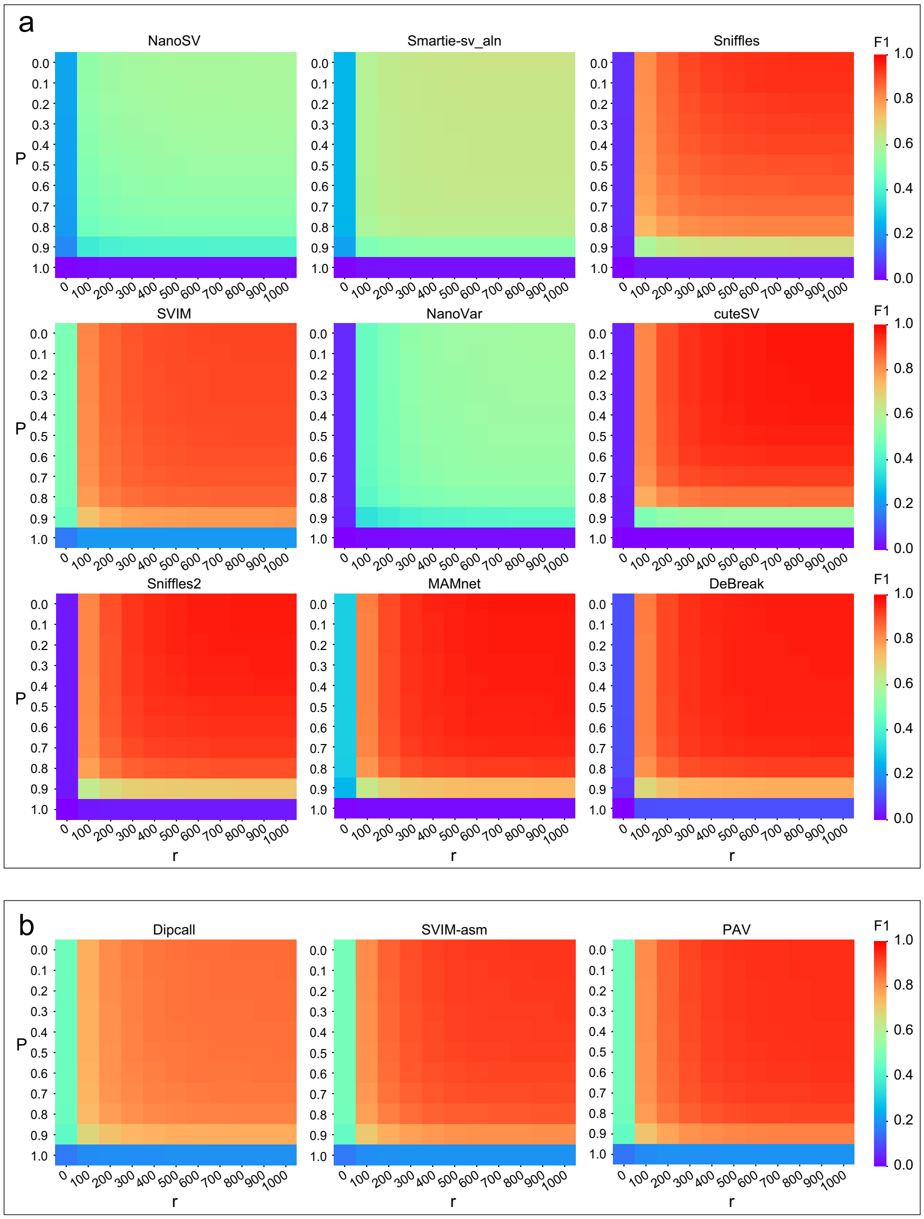

Supplementary Figure 17: F1 accuracy for detection of **insertion SVs** by tuning different evaluation parameters P and r on **Nano_L1**. **a.** Grid search heatmap of F1 values for insertion SVs by different read alignment-based tools. **b.** Grid search F1 heatmap for insertion SVs by different assembly-based tools. P is the minimum percentage of allele size similarity between SV call and gold standard SV, which varies from 0-1 with a 0.1 interval. r is the maximum reference location distance between SV call and gold standard SV, which varies from 0-1000bp with a 100bp interval. Source data are provided as a Source Data file.

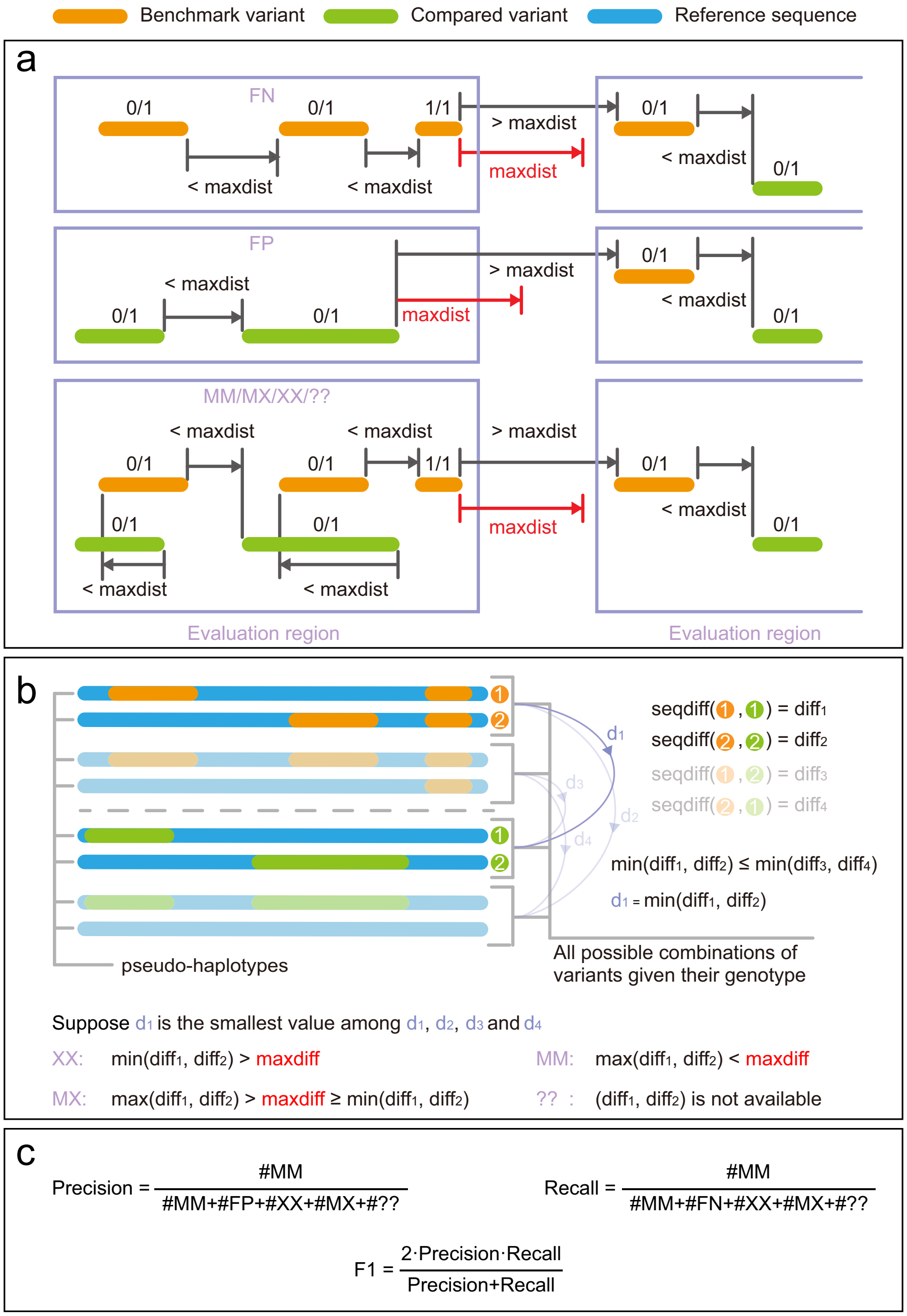

Supplementary Figure 18: The SV evaluation pipeline of hap-eval and defination of *maxdist* and *maxdiff*. **a.** Hap-eval sorts all SVs in benchmark and VCFs being validated by their start coordinates on reference and then calculates the distance between consecutive SVs ($start_{i+1} - end_i$). SVs with distances lower than *maxdist* are clustered to a whole set for validation. **b.** Hap-eval inserts benchmark SVs and SVs being validated back to the corresponding reference sequence separately, and constructs all possible pseudo-haplotype combinations for each VCF based on genotypes of SVs. Pseudo-haplotype combinations from the benchmark are compared to those from VCF being validated to get the combination pair with the lowest sequence difference (default $1-$*Levenshtein ratio*), which is determined by *maxdiff*. **c.** Calculation of F1, precision and recall.

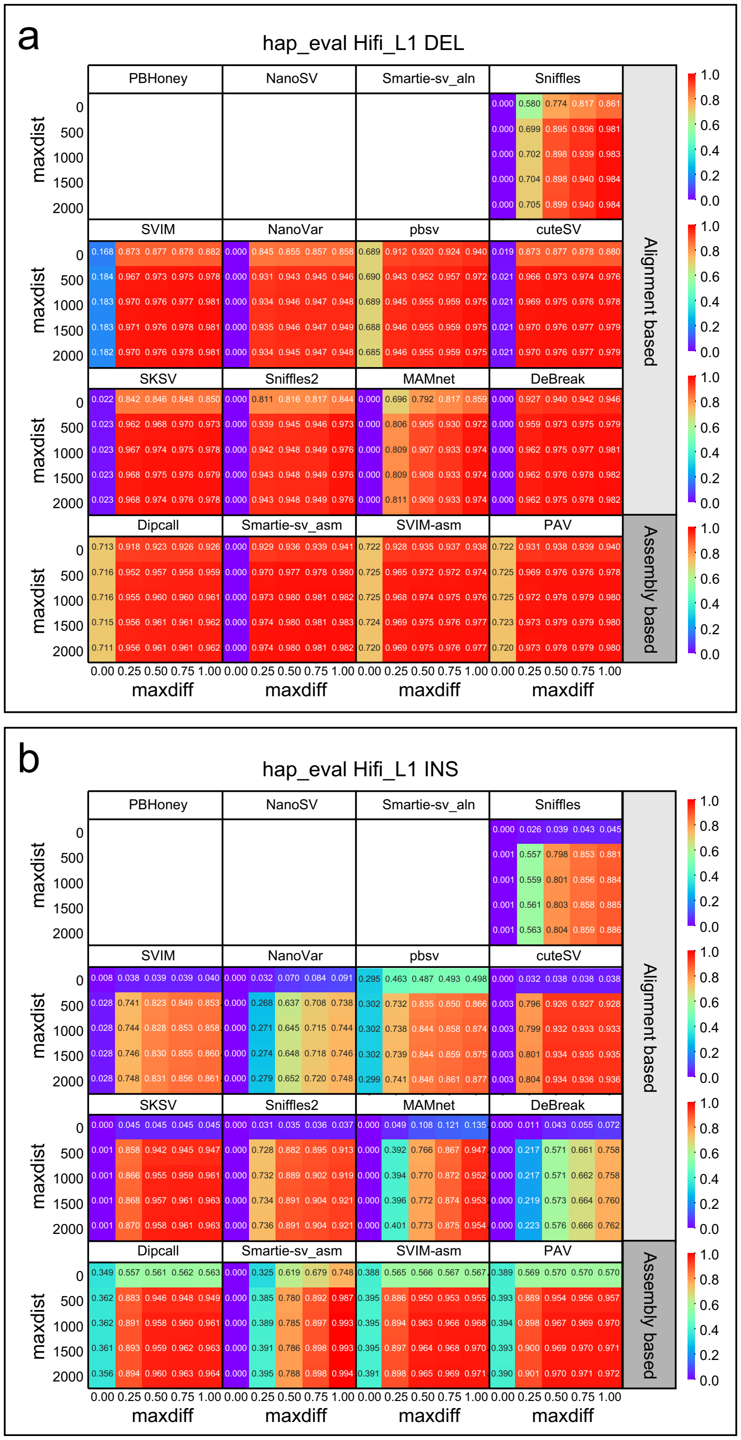

Supplementary Figure 19: F1 accuracy by tuning different evaluation parameters of hap-eval for SVs on Hifi_L1. **a.** Grid search heatmap of F1 values for deletion SVs by different read alignment-based and assembly-based tools. **b.** Grid search F1 heatmap for insertion SVs by different read alignment-based and assembly-based tools. *maxdist* is the maximum reference location distance among multiple evaluated SV calls or gold standard SVs which are validated as a whole set. *maxdiff* is the maximum percentage of sequence similarity between compared pseudo-haplotype and gold standard pseudo-haplotype. *maxdist* varies from 0-2000bp with a 500bp interval. *maxdiff* varies from 0-1 with a 0.25 interval. Source data are provided as a Source Data file.

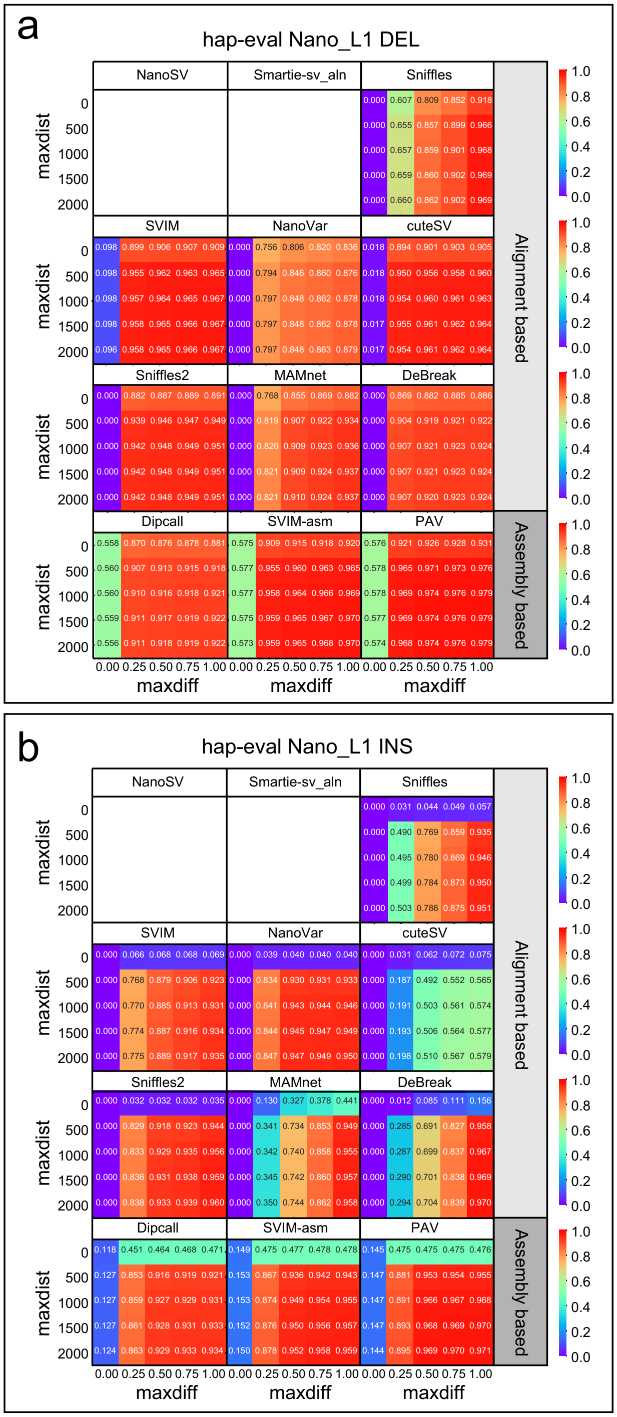

Supplementary Figure 20: F1 accuracy by tuning different evaluation parameters of hap-eval for SVs on Nano_L1. **a.** Grid search heatmap of F1 values for deletion SVs by different read alignment-based and assembly-based tools. **b.** Grid search F1 heatmap for insertion SVs by different read alignment-based and assembly-based tools. *maxdist* is the maximum reference location distance among multiple evaluated SV calls or gold standard SVs which are validated as a whole set. *maxdiff* is the maximum percentage of sequence similarity between compared pseudo-haplotype and gold standard pseudo-haplotype. *maxdist* varies from 0-2000bp with a 500bp interval. *maxdiff* varies from 0-1 with a 0.25 interval. Source data are provided as a Source Data file.

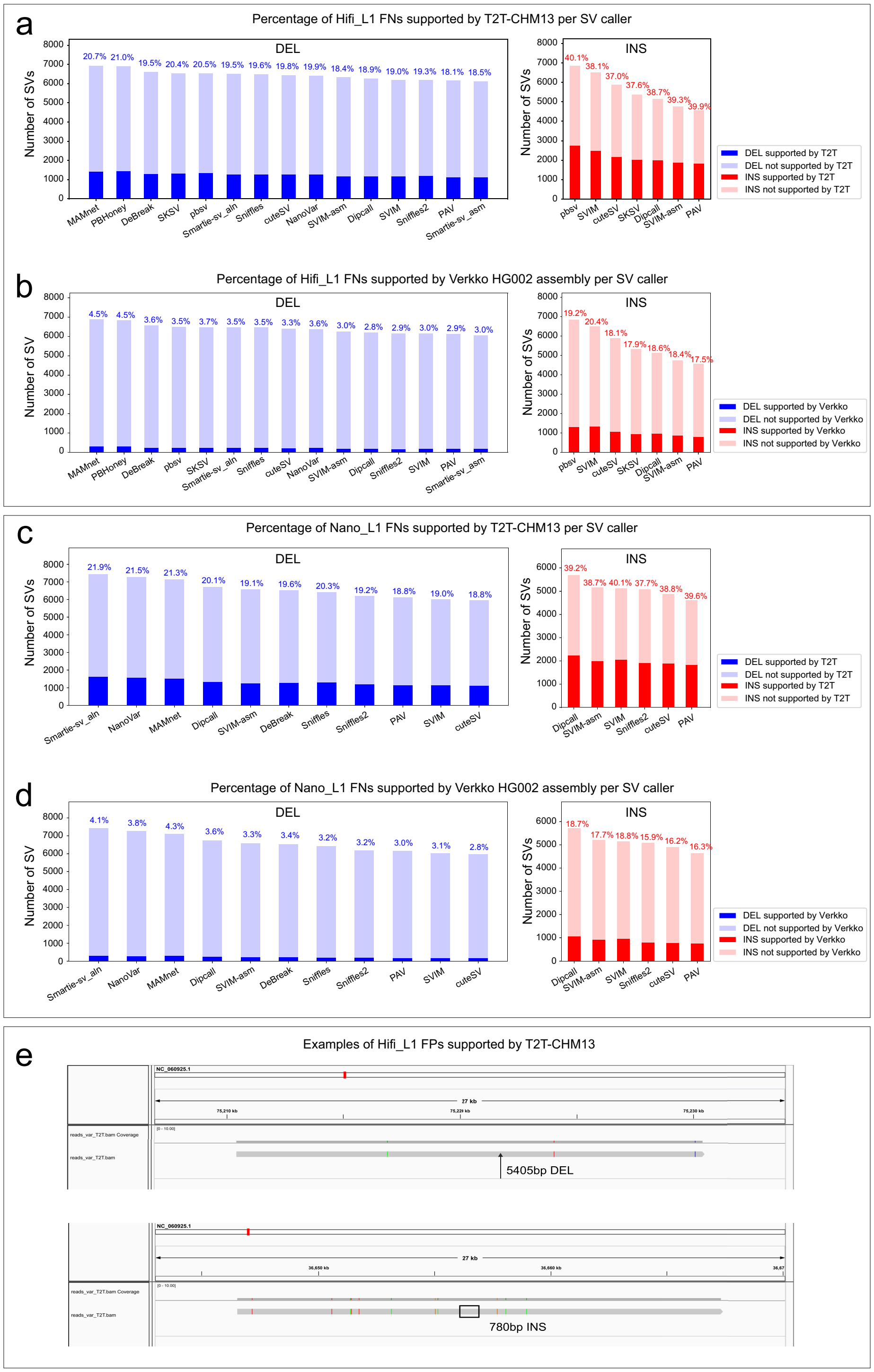

Supplementary Figure 21: Orthogonal SV validation with T2T-CHM13 and Verkko assembly. **a.** Percentage of FN deletions and insertions supported by CHM13-T2T for each SV caller on Hifi_L1. **b.** Percentage of FN deletions and insertions supported by Verkko assembly for each SV caller on Hifi_L1. **c.** Percentage of FN deletions and insertions supported by CHM13-T2T for each SV caller on Nano_L1. **d.** Percentage of FN deletions and insertions supported by Verkko assembly for each SV caller on Nano_L1. **e.** Example of one FP deletion and one FP insertion supported by T2T-CHM13. Source data are provided as a Source Data file.

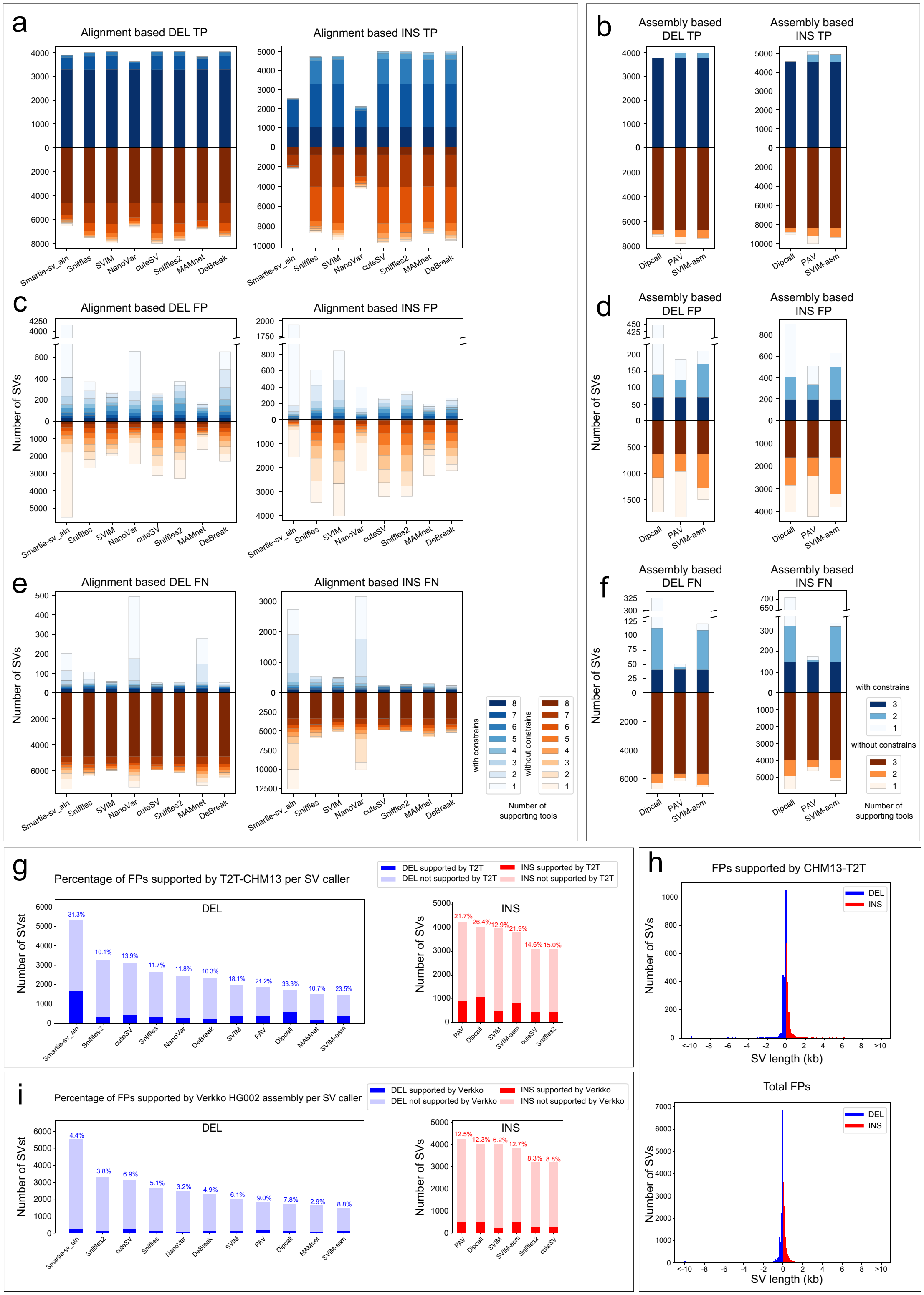
a
Alignment based DEL TP
Alignment based INS TP
b
Assembly based DEL TP
Assembly based INS TP
c
Alignment based DEL FP
Alignment based INS FP
d
Assembly based DEL FP
Assembly based INS FP
e
Alignment based DEL FN
Alignment based INS FN
f
Assembly based DEL FN
Assembly based INS FN
Number of SVs
Smartie-sv_aln
Sniffles
SVIM
NanoVar
cuteSV
Sniffles2
MAMnet
DeBreak
Dipcall
PAV
SVIM-asm
with constrains
without constrains
Number of supporting tools
g
Percentage of FPs supported by T2T-CHM13 per SV caller
DEL supported by T2T
DEL not supported by T2T
INS supported by T2T
INS not supported by T2T
DEL
INS
Number of SVst
h
FPs supported by CHM13-T2T
Total FPs
SV length (kb)
i
Percentage of FPs supported by Verkko HG002 assembly per SV caller
DEL supported by Verkko
DEL not supported by Verkko
INS supported by Verkko
INS not supported by Verkko

Supplementary Figure 22: Orthogonal SV validation with overlapping calls among different tools and with the new complete human genome reference T2T-CHM13 and Verkko assembly on **Nano_L1**. **a-b.** Overlapping TP deletion and insertion SVs supported by read alignment-based and assembly-based tools. **c-d.** Overlapping FP deletion and insertion SVs supported by read alignment-based and assembly-based tools. **e-f.** Overlapping FN deletion and insertion SVs supported by read alignment-based and assembly-based tools. The top panels show results by using high-confidence benchmark SVs from GIAB (with constraints), and the bottom panels show results by using all benchmark SVs (without constraints). The height of each bar represents the total SVs discovered by a tool. Color gradient in the bar specifies a range of supporting tools for SVs. Dark colors represent a high number of supporting tools, while light colors represent a low number of supporting tools. **g.** Percentage of FP deletions and insertions supported by CHM13-T2T for each SV caller. **h.** Size distribution of total FPs (without constraints) and FPs supported by T2T-CHM13. **i.** Percentage of FP deletions and insertions supported by Verkko assembly for each SV caller. Source data are provided as a Source Data file.

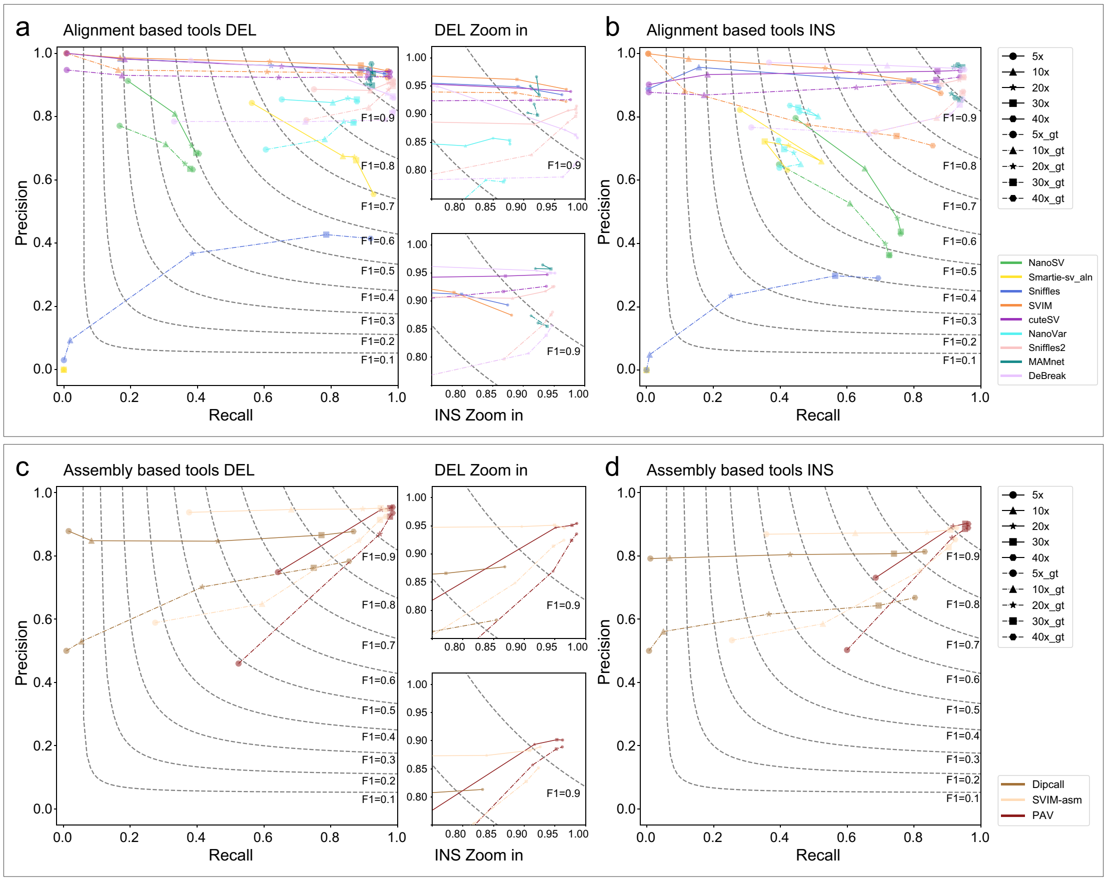

Supplementary Figure 23: Subsampling effect of different SV callers on Nano_L1. **a-b.** Recall-precision-F1 curves show the subsampling effect on deletion and insertion SVs by read alignment-based tools on Nano_L1. **c-d.** Recall-precision-F1 curves show the subsampling effect on deletion and insertion SVs by assembly-based tools on Nano_L1. The coverage depth varies from 5x, 10x, 20x, 30x, to 40x. Solid lines with markers are for different coverage depths, and corresponding dashed lines are for genotyping accuracy. For deletion SVs, we zoom in on the top right part of the plot to demonstrate the curves more clearly. Source data are provided as a Source Data file.

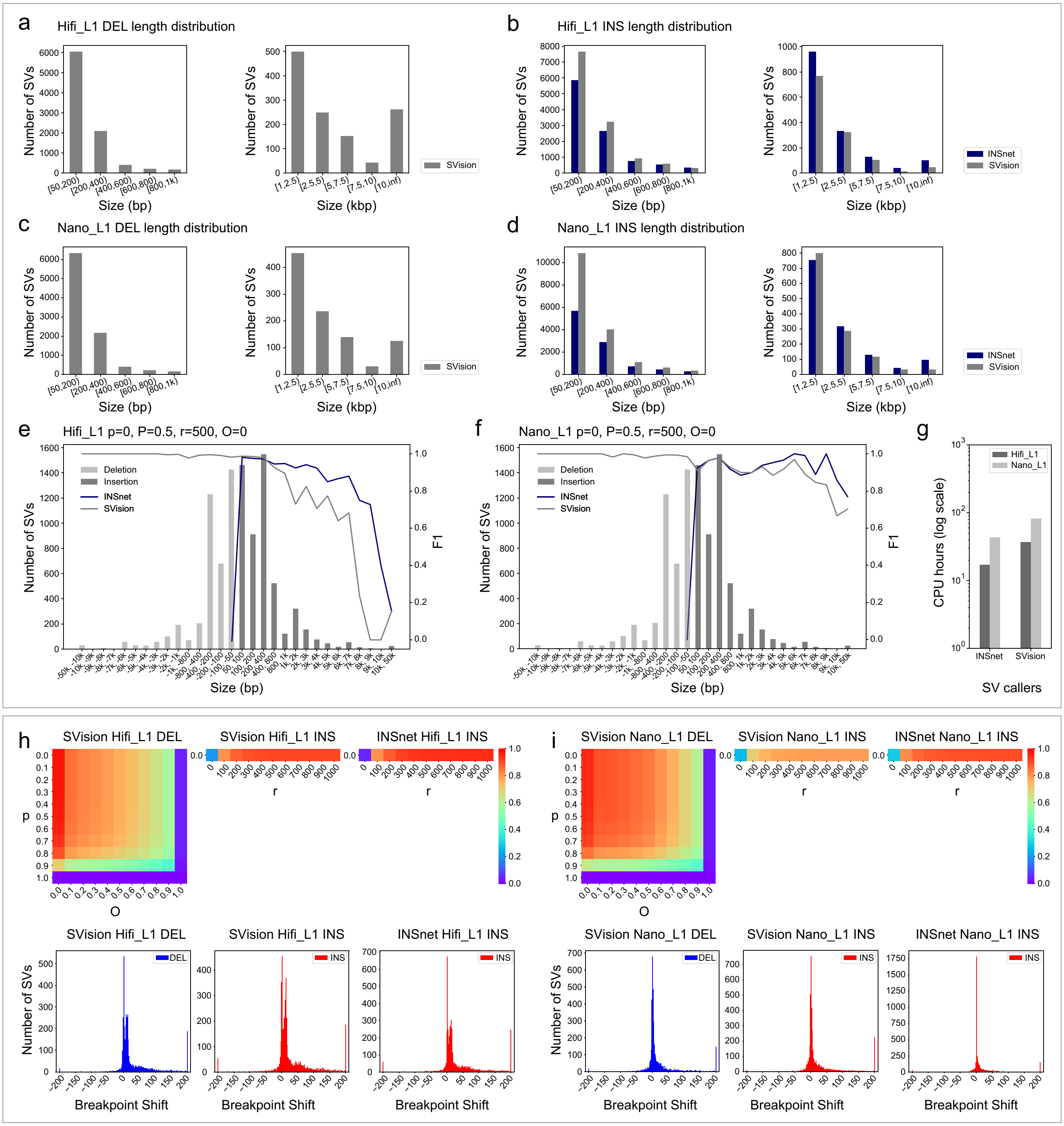

Supplementary Figure 24: Benchmarking I on SVision and INSnet in Hifi_L1 and Nano_L1. **a-f.** Size distribution and accuracy for SV discovery. **g.** CPU time consumption. **h-i.** F1 accuracy by tuning different evaluation parameters and distribution of breakpoint shift for SVs. Detailed legend can be referred to Figures 2 and 3. Source data are provided as a Source Data file.

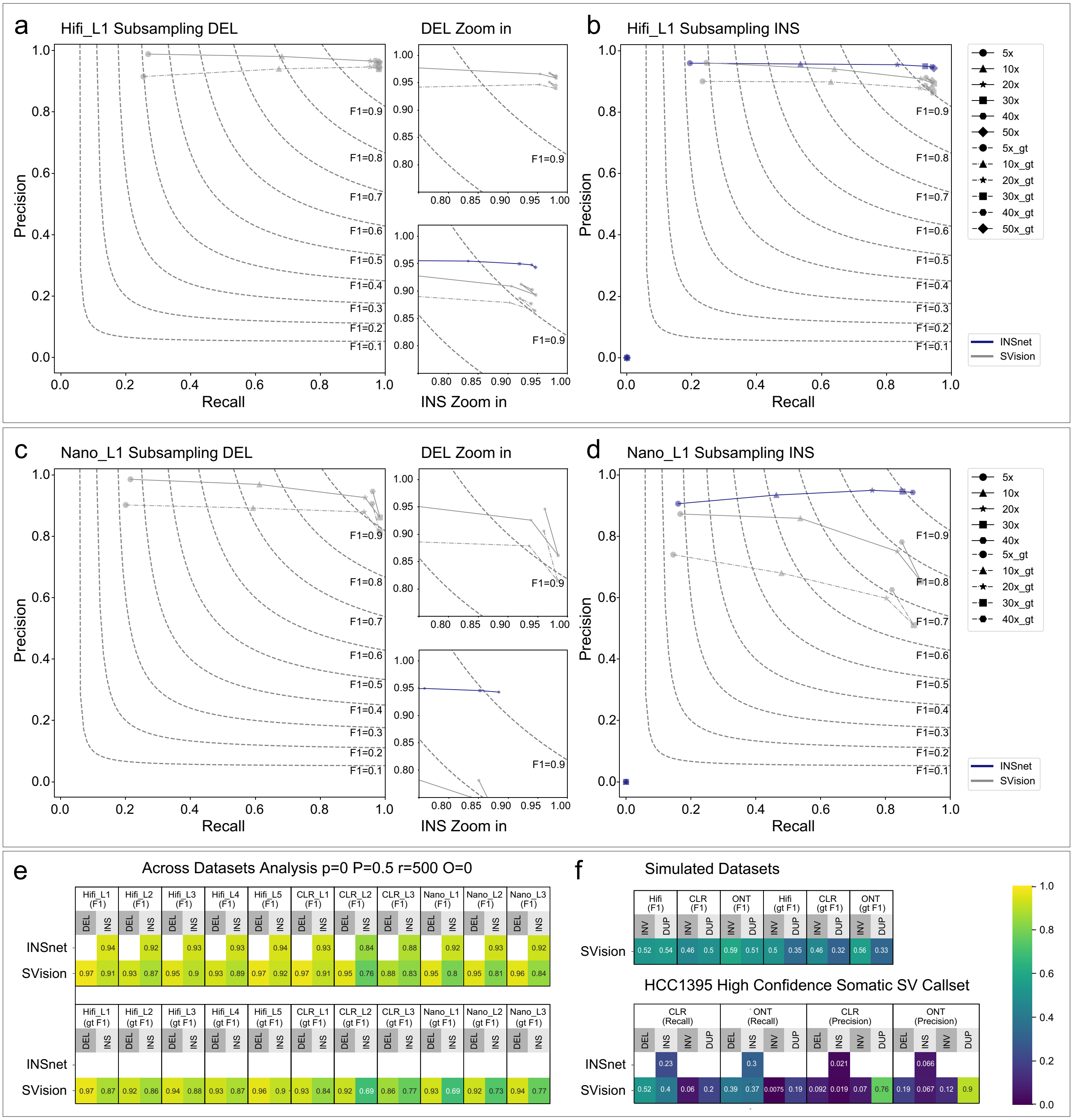

Supplementary Figure 25: Benchmarking II on SVision and INSnet. **a-d.** Subsampling effect analysis. **e.** Across-datasets analysis. **f.** Complex SV detection in simulated and real cancer datasets. Detailed legend can be referred to Figures 5 and 7. Source data are provided as a Source Data file.

Supplementary References

[1] Chaisson, M.J., Tesler, G.: Mapping single molecule sequencing reads using basic local alignment with successive refinement (blasr): application and theory. BMC bioinformatics **13**(1), 1–18 (2012)

[2] English, A.C., Menon, V.K., Gibbs, R., Metcalf, G.A., Sedlazeck, F.J.: Truvari: Refined structural variant comparison preserves allelic diversity. bioRxiv (2022)

[3] RepeatMasker (Open-4.0). `http://www.repeatmasker.org`

[4] Jain, C., Rhie, A., Zhang, H., Chu, C., Walenz, B.P., Koren, S., Phillippy, A.M.: Weighted minimizer sampling improves long read mapping. Bioinformatics **36**(Supplement_1), 111–118 (2020)

[5] Li, H.: Minimap2: pairwise alignment for nucleotide sequences. Bioinformatics **34**(18), 3094–3100 (2018)

[6] Sedlazeck, F.J., Rescheneder, P., Smolka, M., Fang, H., Nattestad, M., Von Haeseler, A., Schatz, M.C.: Accurate detection of complex structural variations using single-molecule sequencing. Nature methods **15**(6), 461–468 (2018)

[7] Ren, J., Chaisson, M.J.: lra: A long read aligner for sequences and contigs. PLOS Computational Biology **17**(6), 1009078 (2021)

[8] A VCF Comparison Engine for Structual Variant Benchmarking. `https://github.com/Sentieon/hap-eval`
