# Supplementary material for: Tradeoffs in alignment and assembly-based methods for structural variant detection with long-read sequencing data
Source: Nat Commun. 2024 Mar 19;15:2447. doi: 10.1038/s41467-024-46614-z (PMC10951360; doi:10.1038/s41467-024-46614-z)
Supplement: Supplementary file 4 — Source Data [file 41467_2024_46614_MOESM4_ESM.zip › Source Data/Source Data.docx]

Zenodo refers to “https://zenodo.org/record/8287836”

**Figure2**

Figure2/SVLEN_distribution/vcf_list_file_example.txt

Figure2/SVLEN_distribution/bin/SVLEN_distribution_analysis.py

Figure2/F1_in_different_SV_size_range/readme

Zenodo/Hifi_L1

Zenodo/Nano_L1

**Figure3**

Figure3/Truvari_Heatmap/Template_Truvari_grid_search.sh

Figure3/Truvari_Heatmap/submit_single_Truvari.sbatch

Figure3/Truvari_Heatmap/tools_truvari_list_example.config

Figure3/Truvari_Heatmap/Truvari_results_heatmap.py

Figure3/BKShift_SeqSim/readme

Zenodo/Hifi_L1

Zenodo/Nano_L1

**Figure4**

Figure4/Overlap/SV_overlap_analysis_twoplots.py

Figure4/Overlap/SV_overlap_analysis_breakaxes.py

Figure4/Overlap/vlf_example.config

Figure4/Overlap/tcf_example.config

Figure4/HG002_analysis/readme

Figure4/T2T_analysis/readme

Zenodo/Hifi_L1

Zenodo/Nano_L1

**Figure5**

Figure5/Subsample/Recall_Precision_F1_subsampling.py

Figure5/Subsample/Input_folder_structure_example.txt

Zenodo/subsample_Nano_L1

Zenodo/subsample_Hifi_L1

Figure5/Across_datasets/Across_datasets_heatmap.py

Figure5/Across_datasets/truvaris

Zenodo/Hifi_L1~5

Zenodo/Nano_L1~3

Zenodo/CLR_L1~3

**Figure6**

Figure6/radar_plot.py

Figure6/perf_list_example.config

Zenodo/Across_aligners

Zenodo/Across_assemblers

**Figure7**

Read simulation:

Figure7/PBSIM3_simulation

Evaluation of SVs called on simulated reads:

Figure7/Simulation_eval/reademe

Zenodo/Simulation

Somatic SV:

Figure7/Somatic/readme

Zenodo/Somatic

**Supplementary** **Table 2-4, 11-12**

General_Utils/Truvari_eval.sh

Zenodo/Hifi_L1

Zenodo/Nano_L1

**Supplementary** **Table 7-10**

General_Utils/Truvari_eval.sh

Zenodo/subsample_Nano_L1

Zenodo/subsample_Hifi_L1

**Supplementary** **Figure 1**

FigureS1/Construct_fasta_for_RepeatMasker.py

FigureS1/Repeat_percent_RepeatMasker.py

FigureS1/extract_unique_annotation.py

FigureS1/Performance_on_different_repeat_annotation.py

FigureS1/Performance_on_different_repeat_percentage.py

Zenodo/Hifi_L1

**Supplementary** **Figure 2-17**

Same pipeline and data as used for Figure3

**Supplementary** **Figure 19-20**

FigureS19/readme

Zenodo/Hifi_L1

Zenodo/Nano_L1

**Supplementary** **Figure 21-22**

Same pipeline as used for Figure4

Zenodo/Hifi_L1

Zenodo/Nano_L1

**Supplementary** **Figure 23**

Same pipeline as used for Figure5, subsampling

Zenodo/Nano_L1

**Supplementary** **Figure 24**

Same pipeline as used for Figure2 and 3

**Supplementary** **Figure 25**

Same pipeline as used for Figure 5 and Figure7

**NOTES:**1. For evaluations that requires Truvari results, please refer to: General_Utils/Truvari_eval.sh
